# Exploration of effects of galvanic vestibular stimulation on circadian rhythms and its associations with sleep and spatial memory in patients with breast cancer: The ICANSLEEP-2 protocol

**DOI:** 10.1371/journal.pone.0306462

**Published:** 2024-07-31

**Authors:** Melvin Galin, Laura de Girolamo, Bénédicte Clarisse, Carine Segura-Djezzar, Franka Glöckner, Clara Elia, Stéphane Réhel, Patrice Clochon, Franck Doidy, Julien Chavant, Olivier Etard, Fausto Viader, Jean-Michel Grellard, Justine Lequesne, Florence Joly, Francis Eustache, Tristan Martin, Bénédicte Giffard, Gaëlle Quarck, Joy Perrier

**Affiliations:** 1 Neuropsychology and Imaging of Human Memory Research Unit, GIP Cyceron-Normandy University-PSL-EPHE-INSERM-Caen University Hospital, Caen, France; 2 COMETE Research Unit, GIP Cyceron-Normandy University-PSL-EPHE-INSERM-Caen University Hospital, Caen, France; 3 Clinical Research Department, François Baclesse Center, Caen, France; 4 Medical Oncology Department, François Baclesse Center, Caen, France; 5 Faculty of Psychology, Chair of Lifespan Developmental Neuroscience, TU Dresden, Dresden, Germany; 6 Cancer and Cognition Platform, French League Against Cancer, Caen, France; 7 Cancer Prevention and Treatment (ANTICIPE) Research Unit, INSERM, Normandy University, Caen, France; 8 Movement – Interactions, Performance (MIP) Team, Faculty of Sciences and Technologies, Le Mans University, Le Mans, France; Public Library of Science, UNITED STATES

## Abstract

**Background:**

Patients with breast cancer (BC) exhibit circadian rhythm disruptions, mainly of rest-activity rhythm (RAR), of which sleep is an essential component, and cortisol rhythm. Sleep complaints such as insomnia and cognitive impairments are prevalent in BC. In general population, sleep is known to contribute greatly to cognition. Thus, improving RAR (and particularly sleep) could help limiting cognitive impairments in BC patients. It has recently been suggested that, in addition to its essential role in spatial memory, the vestibular system contributes to RAR synchronization. Its stimulation could therefore limit both sleep disturbances and spatial memory deficits in BC.

**Objectives:**

The main aim of the ICANSLEEP-2 study is to assess the effects of galvanic vestibular stimulation (GVS) on circadian rhythms. The secondary aim is to assess whether GVS improves sleep and spatial memory in BC patients.

**Methods:**

Two groups with insomnia complaints (Insomnia Severity Index > 7) will be included: a patients’ group with BC (*n* = 50) and a healthy control group without history of cancer (*n* = 25). There will be two assessment sessions, before and after 2 weeks of GVS. Patients will be randomly assigned to either a GVS group or a sham group (noneffective stimulation). Controls will receive GVS. GVS effects will be quantified and compared between groups. Assessments will include actigraphy, salivary cortisol, polysomnography, a cognitive test battery (including a computer-based task for spatial memory) and validated questionnaires (for psychological functioning and sleep complaints).

**Discussion:**

Current methods for improving sleep in BC have had controversial outcomes regarding sleep structure. We expect GVS to offer a new mean of directly targeting RAR disruptions in BC patients, with beneficial effects on sleep structure. Given the crucial impact of sleep on cognitive functioning, notably spatial memory, improving sleep of BC patients should enhance their cognitive functioning.

**Ethics and dissemination:**

This study received ethical approval from the Ile de France IV institutional review board on 19 April 2022 (no. ID-RCB: 2022-A00437-36). The findings yielded by this protocol will be presented at various conferences and in peer-reviewed journals.

**Clinicaltrials.gov registration number:**

NCT05414357.

## 1. Introduction

Breast cancer (BC) is a noncentral nervous system (CNS) cancer that affected more than 2 million women worldwide in 2020, making it the most frequently diagnosed cancer in women. BC is also a cancer with a high survival rate (exceeding 90% in the wealthiest countries) [1]. This survival is associated with negative effects such as fatigue and depressive symptoms that have already been documented [2, 3]. Other negative effects include sleep complaints, which can be linked to circadian disruptions.

These disruptions are frequent [4], and have mainly been studied using actigraphy [5, 6], which measures the rest-activity rhythm (RAR; i.e., alternation between periods of sleeping and waking). RAR disruptions in BC are notably reflected by a decrease in amplitude (difference between lowest and highest activity levels) or mesor (activity-adjusted mean) [7–10]. RAR disruptions can be observed in patients with BC whether or not they have undergone chemotherapy [3, 11, 12]. These disruptions have been associated with cognitive impairment in diverse populations (aging, cancer, dementia, etc.) [12–15].

Circadian disruptions can also take the form of cortisol rhythm disruptions, with decreased variance in cortisol secretion or a flattened diurnal cortisol slope [16–18]. The cortisol awakening response may also be affected [19]. These disruptions are believed to stem from altered hypothalamic-pituitary-adrenal axis responsiveness. In addition to the later peaks and diminished amplitudes, some studies have shown that, compared with controls (e.g., nonfatigued survivors of BC), cortisol rates remain relatively high at bedtime [17, 20–22].

Sleep is an essential component of RAR, and about 20-70% of patients with BC complain about disturbed sleep, mostly insomnia (i.e., “difficulty initiating or maintaining sleep, or nonrestorative sleep”) [23]. This prevalence is more elevated than in the general population and in other nonCNS cancers [24]. These sleep complaints can start prior to receiving the diagnosis and persist for months and even years after treatment, peaking during chemotherapy [25]. They have been partly confirmed after adjuvant chemotherapy using objective measures of sleep, namely actigraphy and polysomnography (PSG) [26–29]. These studies highlighted greater sleep fragmentation and lower sleep efficiency in patients than in healthy controls.

Sleep disruptions (whether or not they are related to wider RAR disruptions) are known to be related to memory deficits, notably affecting spatial memory [30–32], in the general population and other pathologies [33–35]. This link continues to be neglected in patients with BC [2, 36–39], despite the prevalence of persistent cognitive deficits after chemotherapy in this population [40, 41]. Given the negative effects of both disturbed sleep and cognitive impairments on the quality of life of patients with BC [42, 43], it is important to look for ways of alleviating them.

Several methods are currently available for treating these sleep disruptions, including medication [44, 45], cognitive-behavioral therapy (CBT) [46, 47], and adapted physical activity (APA) [48, 49]. Sleep medication may have several side effects, such as headaches, back pain, and digestive issues [50]. They can also create addiction in the general population [51]. Both CBT [47, 52] and APA [49, 53] have been shown to have positive effects on sleep, as measured by two different means. When assessed with questionnaires, better sleep efficiency or shorter sleep onset latency for CBT, and improved sleep quality for APA, have been reported by patients with BC during and after disease. When assessed with actigraphy, CBT has been shown to have a small to medium effect in decreasing sleep onset latency or reducing the frequency of sleep interruption. Some of the effects on sleep onset latency may last for up to 6 months after the intervention [54]. While CBT is the standard treatment for insomnia-related difficulties [47], there is a need for a new approach that directly targets RAR and improves sleep structure. An innovative approach tested in several studies involves the vestibular system, on account of its inherent properties and the effects of its stimulation on RAR [55].

The vestibular system is located in the inner ear. Its properties include the detection of head movement and gravity for the purpose of balance control. It therefore sends various types of information to the brain to help individuals locate themselves (e.g., angular and linear accelerations, gravity), thus playing an essential role in spatial memory [56]. Vestibular stimulation through galvanic vestibular stimulation (GVS) can enhance spatial memory performances [57]. Plus, it has been shown that the hippocampus and episodic memory (which has a spatial component) are affected in the context of breast cancer [58].

Various types of vestibular system stimulation have also been found to reduce sleep onset time and the number of nocturnal awakenings among healthy volunteers [59–62]. Indirect links have been demonstrated between the vestibular system and the sleep timing system [63], suggesting a relationship between the vestibular system and circadian rhythms. This system may, in fact, be a nonluminous RAR synchronizer [64, 65], as previous reports have suggested that vestibular stimulation can improve RAR, notably by reducing mean activity the evening following stimulation [66]. This could be very useful in the context of BC, in that it could allow patients to sleep better at night, and thus function better during the day.

The ICANSLEEP-2 study will follow on from ICANSLEEP-1, a study [67] designed to precisely characterize sleep, circadian rhythms, and their association with cognitive impairments in patients with BC.

The main objective of ICANSLEEP-2 will thus be to assess the effects of GVS on circadian rhythms (here, RAR and cortisol) in patients with BC who have already completed their treatment (either chemotherapy, radiotherapy, or both). We hypothesize that GVS will lead to greater rhythm amplitude during the day and lower nighttime activity, compared with noneffective stimulation. A group of participants without cancer will also receive GVS, in order to ensure that the beneficial effects of GVS are not specifically related to cancer.

As a secondary objective, we will assess the effects of circadian rhythm regulation on sleep and cognition, testing the hypothesis that it can lead to fewer awakenings at night and smaller cognitive deficits, compared with noneffective stimulation. Finally, we will assess the direct effects of GVS on both sleep and cognition.

## 2. Methods and analysis

### 2.1. Participants

Two groups of participants will be recruited: a group of women who have been treated for BC with chemotherapy and/or radiotherapy (*n* = 50) and a group of healthy female volunteers without any history of cancer (HC; *n* = 25).

Each group will be composed of participants aged 45-65 years, and controls will be matched with patients for age and education level. All participants will have sleep complaints (Insomnia Severity Index score ≥ 8).

Patients will be invited to take part in the study during their appointments with their oncologists at the François Baclesse cancer treatment center in Caen, Normandy (France). The medical practitioners and clinical research associates will identify potential eligible patients during their follow-up. Patients will be given some information on the study, and if they are interested in taking part, they will have to sign an informed consent with a medical doctor form before being enrolled in the study. For detailed information about the eligibility criteria, see Table 1.

**Table 1:** ICANSLEEP-2 inclusion and exclusion criteria.

Patients with breast cancer	Healthy individuals
*Inclusion criteria*	
Age 45–65 years	
French native speaker	
At least Level 3 (end of primary school) on Barbizet scale	
Normal cognitive function (Montreal Cognitive Assessment score ≥ 26)	
Insomnia Sleep Index (> 7)	
Operated for local breast cancer	
Treated with adjuvant chemotherapy	
	Women with no history of cancer
*Noninclusion criteria*	
Neurological sequelae	
Personality disorders and progressive psychiatric disorder	
Night shifts work	
Drug use and/or heavy drinking	
Central nervous system tumor	
Cognitive disorders prior to cancer diagnosis	
Primary cancer different from breast cancer	
Metastatic cancer	

The recruitment period will range from 19^th^ of April 2022 to the 19^th^ of August 2026.

Control participants will be recruited by various means, ranging from flyers to social media posts. They will then contact us and we will have a pre-inclusion phone appointment. They will then have to come to our laboratory for the inclusion appointment.

### 2.2. Study design

The ICANSLEEP-2 is a two-center longitudinal follow-up study of ICANSLEEP-1, that aims to describe sleep alterations in patients with BC, some treated with chemotherapy. Those two projects are part of a bigger one named “ICANSLEEP” (Impact of SLEEP disruption on Cognition and quality of life in breast cANcer).

ICANSLEEP-2 will have two sessions. The first session (T1) will take place 2 weeks after the completion of treatment or an equivalent timing for those not undergoing any treatment. The second session (T2) will take place 2 weeks after T1. During this 2-week interval, participants will perform GVS sessions at home in the presence of an experimenter. Each session will last 2 days, interspersed by a night of sleep.

**Fig 1:**
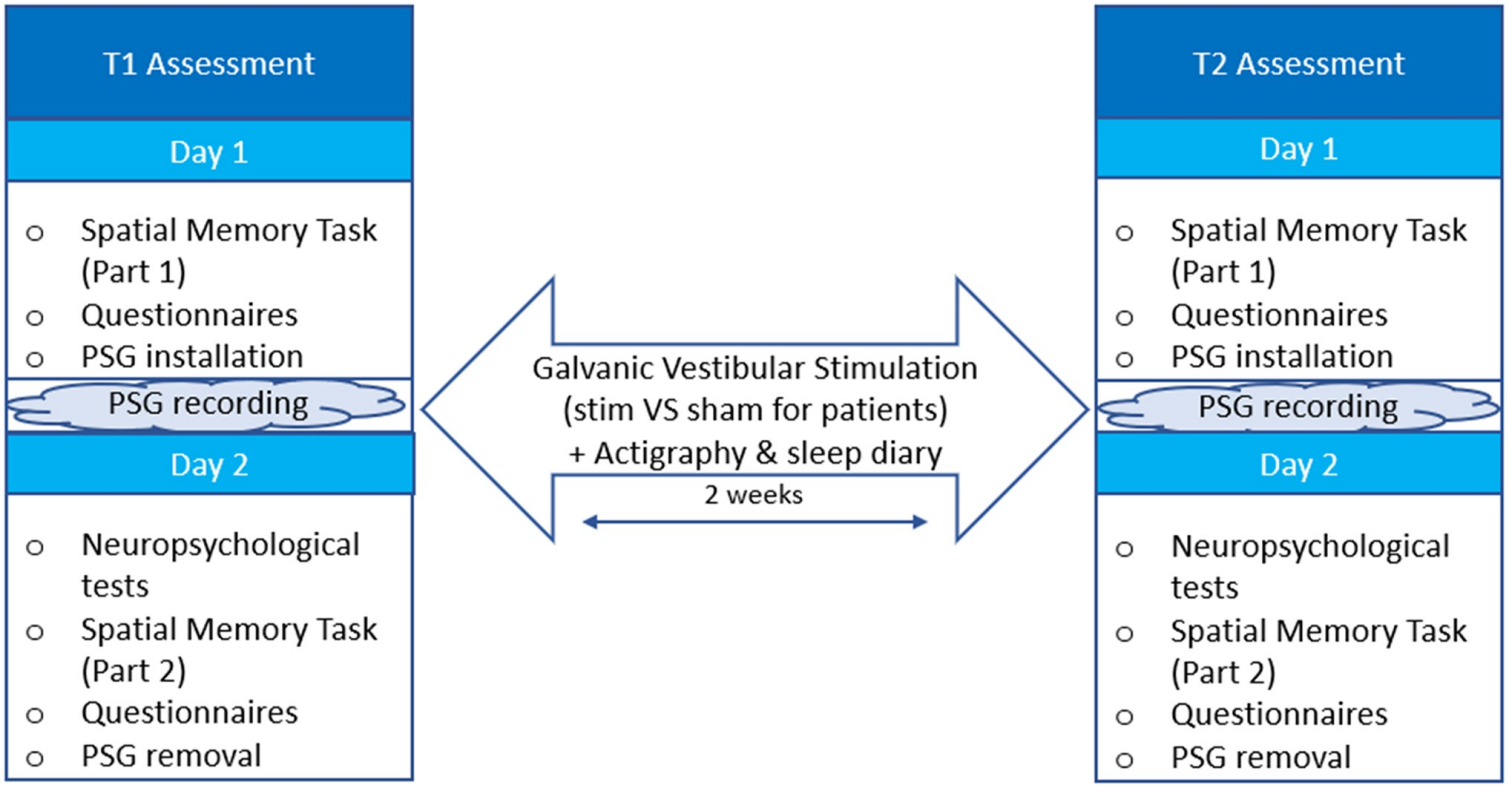
ICANSLEEP-2 trial design.

The study design is illustrated in Fig 1. Cognitive tests (with their outcome measures and score ranges) are listed in Table 2, and questionnaires (including score ranges) in Table 2.

**Table 2:** Cognitive domains assessed in ICANSLEEP-2 study, assessments tests and their outcome measures and ranges.

Cognitive domains	Assessment tests	Outcome measures	Ranges
Episodic memory	HVLT	Total free recall score	0–36
		Delayed recall score	0–12
Working memory	WMS-III spatial memory subtest	Total number of correct trials forward	0–16
		Total number of correct trials backward	0–16
Attention (divided attention)	Baddeley dual task	Digit span: number of correct sequences	
		Motor Task: number of boxes checked in 2 minutes	
		Dual task: number of correct sequences; number of boxes checked	
Sustained attention	D2-R	Number of target characters processed	
		Concentration abilities: CCT—errors	
		% errors: (errors/CCT) x 100	
Executive functions	*n*-back	Number of correct responses	0–48
	TMT (A–B)	Processing speed Part A	
		Processing speed Part B—A	≥0 s
	Stroop	Naming time	
		Processing speed	≥0 s
		Interference—naming time	
	Lexical fluency (*P* & *R*)	Number of words produced	0—x

*Note*. HVLT: Hopkins Verbal Learning Test; WMS-III: Wechsler Memory Scale– 3^rd^ edition; D2-R: d2 Test of Attention-Revised; TMT: Trail Making Test; CCT: processing speed.

Two weeks before T1, participants will receive a MotionWatch8 actigraph. In the afternoon of Day 1, participants will hand in self-report questionnaires about their quality of sleep and quality of life that they have filled out at home. They will be asked to respond to other questionnaires assessing psychological factors in our laboratory, in the presence of a neuropsychologist belonging to our team. They will then undergo the first part of the spatial memory task (see task description below). After these assessments, PSG equipment will be fitted to objectively measure their sleep.

In the morning of Day 2, participants will complete the first half of the cognitive tests, respond to other questionnaires, and perform the second half of the spatial memory task. The PSG equipment will then be removed, and participants will complete the remaining cognitive tests. Next, our team will show participants how the GVS device works, and give them another MotionWatch8 actigraph to place on their nondominant wrist to assess activity and the said GVS device.

T2 will be identical to T1, except for the introduction to the GVS device. Participants will also return the actigraph.

### 2.3. Circadian rhythms and sleep assessments

Participants’ chronotype and subjective sleep complaints will be assessed via the self-report questionnaires. Two circadian rhythms will be measured: RAR (actigraphy), and diurnal cortisol rhythm (saliva tests). PSG will be used to measure objective sleep quantity and quality, as this is the gold standard method for sleep architecture quantification [68].

#### 2.3.1. Circadian rhythm assessments

a) Chronotype questionnaire

Scores on the Morningness-Eveningness Questionnaire [69] will be used to determine each participant’s chronotype (i.e., morning person, evening person, or neither):

- > 58 points = morning person
- < 42 points = evening person
- 58 ≥ score ≥ 42 points = neither

These cut-off scores, adapted to the French population, were defined by Taillard and colleagues [70].

b) Actigraphy

An actigraph (MotionWatch8; CamNtech, UK) placed on the nondominant wrist will be used to assess participants’ RAR throughout the 2 weeks of GVS at home. This actigraph contains a tri-axial accelerometer (range: 0.01–8 g) that will provide information about participants’ diurnal and nocturnal activity levels and indirectly assess their sleep characteristics. The epoch (interval between each measurement) will be set at 15 seconds. Finally, to ensure the accuracy of the sleep data yielded by the actigraph, they will be coupled with a sleep diary.

c) Salivary cortisol

Participants will be asked to give samples of saliva using the Salivette^®^ saliva collection system, in order to measure cortisol levels at different timepoints. They will perform the saliva sampling themselves at home / in a hotel at 10 p.m. on Day 1 and then at their awakening, and 30 and 45 minutes after awakening, on Day 2. On Day 2, they will also have to do a cortisol test before and after a 11-minutes rest period wearing the PSG equipment (at around 10 a.m.).

#### 2.3.2. Sleep assessments

a) Self-report questionnaires

At both timepoints, we will use the Insomnia Severity Index (Morin et al., 2011) and the Pittsburgh Sleep Quality Index (Buysse et al., 1989) to assess sleep efficiency and quality of sleep, along with insomnia symptoms. The insomnia score ranges from 0 to 28 (cut-off score: 8). The sleep quality score ranges from 0 (perfect sleep) to 21 (very disturbed sleep).

b) Polysomnography

The sleep assessment with the PSG equipment (Siesta, Compumedics) will be conducted either in the participant’s home or at the hotel. Brain activity (electroencephalography), eye movements, heart rate, respiratory rate, and oxygen saturation will be recorded simultaneously, yielding quantitative data on sleep onset latency, sleep efficiency, number of nocturnal awakenings after sleep onset, total sleep time, and relative percentages of sleep stages. Electrodes will be placed on the scalp, over prefrontal (FP1/FP2), frontal (F3/F4/F7/F8/Fz), central (C3/C4/Cz), temporal (T3/T4), parietal (P3/P4/Pz), and occipital (O1/O2) sites, according to the international 10–20 system, using Ag/Au electrodes with a ground and a bi-mastoid reference. The impedance for all electrodes will be kept below 6 kΩ. The bandpass filter will be 0.15–210 Hz, and the sample rate will be 256 Hz. An electrode will be placed above and below each eye to record eye movements, and two electrodes will be placed on the chin to measure muscle tone. An electrocardiogram will also be recorded via an electrode placed below each clavicle. To detect potential sleep apneas or hypopneas, participants will also wear thoracic and abdominal belts to record respiratory movements, a microphone to detect snoring, oral and nasal thermistors to measure airflow, and a finger pulse oximeter to record oxygen saturation parameters.

### 2.4. Cognitive assessments

#### 2.4.1. Spatial memory task

**Fig 2:**
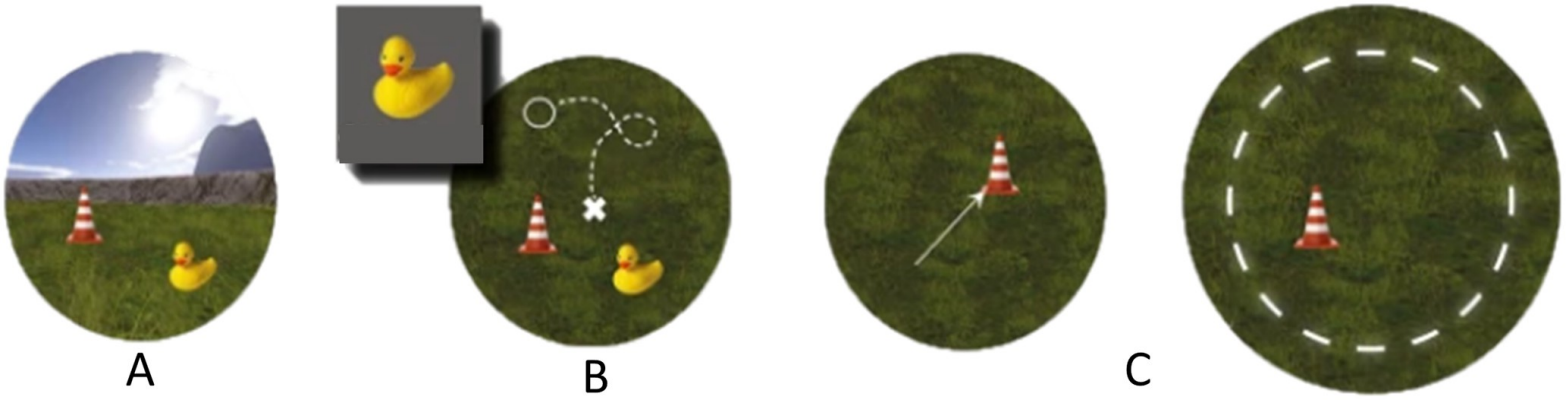
General design of spatial memory task (adapted from Hilliard et al., 2019). Computer-based spatial memory task. Task performed on two successive days and featuring three distinct phases. On Day 1, all three phases will take place. On Day 2, only Phases B and C will take place. A: first phase; B: second phase; C: third phase. A: Participant’s point of view. Participants have to collect objects in the arena. B: Top view. Participants have to put back the objects they collected. C: Top view. Participants have to put back the objects they collected in a modified environment. This modification will consist of either the movement of an intra-labyrinthic cue (plot) or an enlargement of the perimeter wall.

Spatial memory is crucial for orientation, and is often assessed according to the strategy (allocentric or egocentric) used to navigate an environment [57, 71]. With the egocentric strategy, individuals use themselves as a landmark and orient themselves accordingly (e.g., “From my starting point, I go straight ahead for 30 meters, then turn left”). With the allocentric strategy, individuals use external landmarks and a cognitive map to reach their destination [72, 73]. We will use a spatial memory task that relies on proxies of both allocentric and egocentric strategies (boundary-dependent vs. cue-dependent spatial navigation), adapted from [74, 75]. The task will be performed on a computer, in a circular desktop virtual reality environment surrounded by a stone wall (boundary) comprising an intramaze cue (landmark) and extramaze cues (mountains, sun, stone wall, and clouds). This task will take place in two sessions (on Day 1 and Day 2) divided into several phases. The general design of the task is illustrated in Fig 2.

On Day 1, participants will learn how the controls of the task work in a training environment, and will perform the first task session comprising three phases in the task’s environment. The first phase (encoding) will require participants to find four objects in the environment and memorize their position. In the second phase (feedback-based learning and retrieval), participants will have to put the objects back in their original position. During this phase, they will receive feedback from the computer, telling them if they have correctly placed the object. If they cannot recall the exact position, they will be asked to recollect the object and reencode its position. In the third phase (transfer after change in the environment), the environment will be changed, with modifications to either the landmark’s position or the boundary’s diameter. During this phase, participants will have to put the objects back without feedback. They will not be informed about the exact changes in the environment.

On Day 2, participants will repeat Phases 2 and 3, with the same four objects and object positions as on Day 1.

We will compare Phases 1 and 2 to assess memory consolidation during sleep. Participants will perform three trials per object (even if they are successful the first time) in Phase 2, and two trials per object in Phase 3 (one trial per environment modification).

On the second phase, the information retrieved will be the distance between the placed object and its real position, measured in virtual meters (vm). The vm parameter will allow to have an indication of spatial memory performance. The more vm a participant has for an object, the least her performance will be good. Vm values will be the averaged between the 3 trials for each object.

During third phase, a deviation angle will be calculated. This angle will take in account the deviation between where the object is placed and its real position. The angles ThetaLM and ThetaB will be calculated respectively when the boundary enlarges and when the location cue moves. Theses angles will be used to infer a strategy to the participant (either based on intramaze cue or based on the boundary). The more the value of the angle is elevated, the least the participant used the strategy based on this change of environment. For example, if the participant uses a boundary-based strategy, she will place the objects wrong when the boundary enlarges, thus leading to a strong deviation angle and therefore an elevated ThetaLM.

#### 2.4.2. Neuropsychological test battery

At each timepoint, cognitive performances will be assessed on Day 2 using a cognitive test battery (see Table 2). Episodic memory will be assessed using the Hopkins Verbal Learning Test [76], standardized and adapted for French populations. Working memory will be assessed using the Spatial Memory forward and backward subtest (Wechsler Adult Intelligence Scale-III). Attention will be assessed with Baddeley’s dual task [77] and the D2-R [78]. Executive functioning will be assessed with the *n*-back task developed for the AGING protocol [79], Trail Making Test [80], Stroop test [81], and lexical fluency (letters P & R; [82]).

### 2.5. Standardized validated self-report questionnaires related to quality of life

At each timepoint, participants will complete self-report questionnaires on Day 1 or at home the day before to assess their subjective vision of their cognitive functioning as well as psychological factors and quality of life (see Table 3). The Functional Assessment of Cancer Therapy Cognitive Scale (FACT-Cog) [83] and the Functional Assessment of Cancer Therapy–General (FACT-G) [84] will be used to assess their cognitive complaints and general cognitive functioning. Pain will be assessed with the Brief Pain Inventory (BPI) [85]. Feeling of fatigue will be assessed with the Functional Assessment of Chronic Illness Therapy (FACIT-F) [86] and Multidimensional Fatigue Inventory (MFI-20) [87]. The International Physical Activity Questionnaire (IPAQ) [88] will be used to assess physical activity practice. The State-Trait Anxiety Inventory for Adults (STAI-Y) [89] and Beck Depression Inventory (BDI-II) [90] will be administered to obtain covariates and strengthen the statistical analysis.

**Table 3:** Self-report administered in the ICANSLEEP-2 study and their outcome measures and ranges.

Self-reports	Questionnaires	Outcome measures	Ranges
*Chronotype & sleep complaints*			
Chronotype	Morningness-Eveningness (Horne & Ostberg)	Total score	16–86
Insomnia complaints	ISI	Total score	0–28 (≥ 8)
Sleep disturbances	PSQI	Total score	0–21
*Cognitive complaints and functioning*			
Cognitive complaints	Fact-Cog	PCI	0–72
		PCA	0–28
		QoL	0–16
		Oth	0–16
General cognitive functioning	FACT-G (patients)	Total score	0–108
*Quality of life*			
Pain	BPI	Severity of pain	0–10
		Interference of pain	0–10
Fatigue	FACIT-F (patients)	Total score	0–52
	MFI-20	General and physical fatigue	9–45
		Reduced activity	3–15
		Reduced motivation	2–10
		Mental fatigue	6–30
Physical Activity	IPAQ	Continuous score	MET-minutes
		Categorical score	Low/Moderate/High
*Psychological factors*			
Anxiety	STAI-Y	State score	20–80
		Trait score	20–80
Depression	BDI-II	Total score	0–39

*Note*. ISI: Insomnia Severity Index; PSQI: Pittsburgh Sleep quality Index; FACT-COG: Functional Assessment of Cancer Therapy–Cognitive Function; PCI: perceived cognitive impairment; PCA: perceived cognitive abilities; QOL: impact on quality of life; Oth: comments from others; FACT-G: Functional Assessment of Cancer Therapy–General; BPI: Brief Pain Inventory; FACIT-F: Functional Assessment of Chronic Illness Therapy–Fatigue; MFI-20: Multidimensional Fatigue Inventory; IPAQ: International Physical Activity Questionnaire; STAI-Y: State-Trait Anxiety Inventory; BDI-II: Beck Depression Inventory-2^nd^ edition.

### 2.6. Galvanic vestibular stimulation

**Fig 3:**
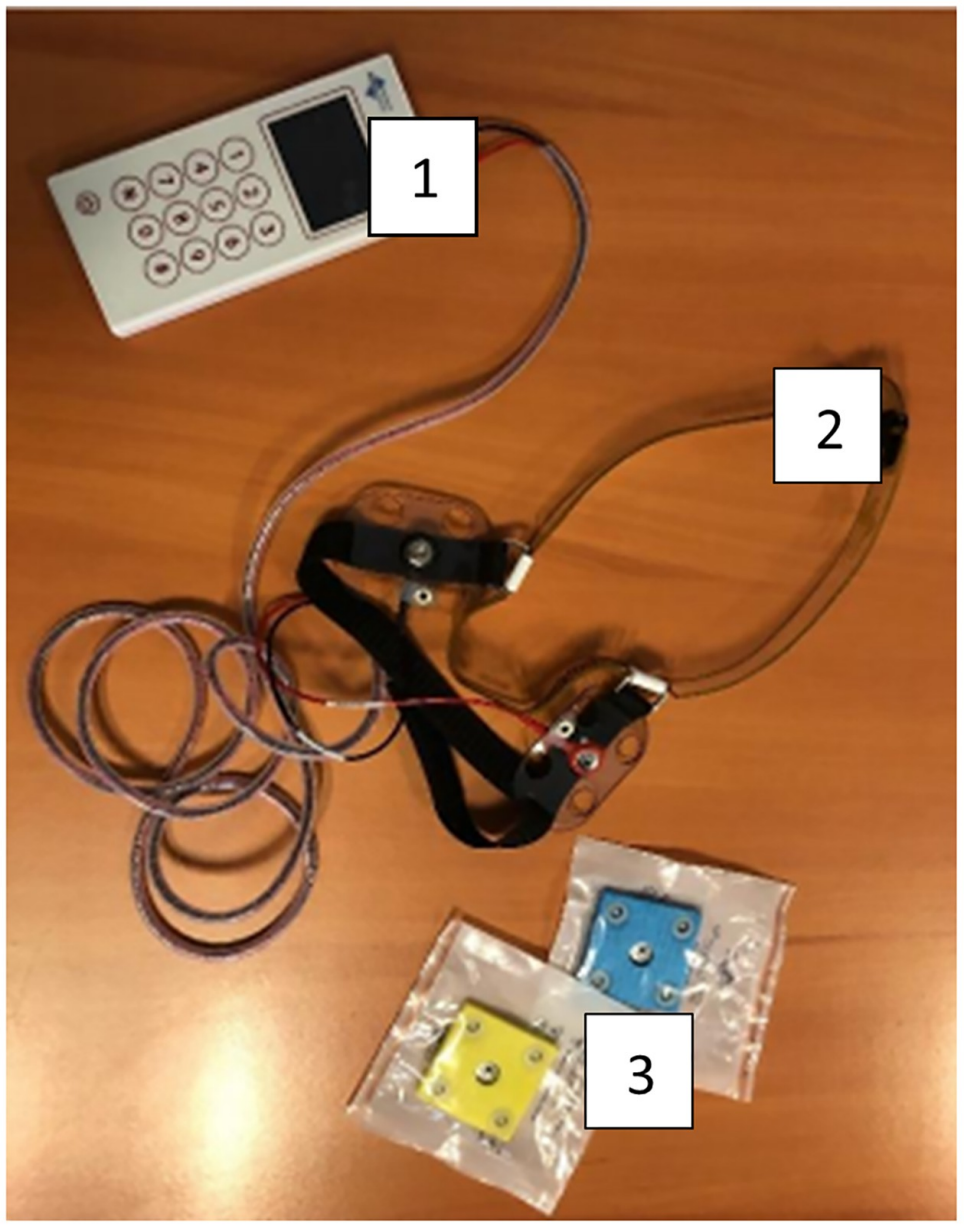
GVS device. 1. GVS generator. The device delivers the electrical current based on the parameters entered beforehand by the experimenter. 2. Headband. This plastic strip is put on the participant’s head with an arrow pointing to the forehead. 3: Sponges. Sponges containing electrodes. These sponges have to be clipped to the bottom of the headband. They allow the current to be delivered behind the ears.

The battery-powered GVS device (Soterix Medical) (presented in Fig 3) will be preprogrammed by the experimenter to deliver codes for stimulation sessions (one code for each session and two backup codes; 12 codes in total). Before they receive the device, we will explain to participants how to put it on correctly and show them how it works.

For each session, participants will enter the dedicated code and the device will deliver an electric current for 20 minutes, initiated by a 60-s ramp-up and terminated by a 60-s ramp-down. In the sham group, patients will feel the ramp-up and the ramp-down, but no current will be delivered in between.

Before entering the code, participants will put on a plastic headband to which they will clip two sponges containing electrodes. The electrodes will be plugged into the device and placed behind the ears, on the mastoid bone. Once this is done, participants will launch the session with the code we have given them.

After each session, three scales (rated 1–7) will be evaluated to know the participant’s feelings about the session. We will have a fatigue scale, a well-being scale and a nausea scale. 1 is the lowest intensity and 7 the highest.

These sessions will take place in the participant’s home every morning (45 minutes after they got out of bed), from Monday to Friday, while video-conferencing with an experimenter from the team. Participants will thus undergo a total of 10 GVS sessions.

### 2.7. Statistical considerations

The number of participants in this descriptive and exploratory study is not subject to power calculation. For more details see [67]. In order to assign the sham or stim group, a 1:1 randomization will be done by the Centre François Baclesse statisctics department.

Data analyses will provide frequencies with their exact 95% confidence interval for qualitative variables, and the mean, standard deviation, median, and quartiles for quantitative variables.

Repeated measures analyses of variance (ANOVA) will be applied to longitudinally compare circadian rhythms, sleep and cognitive variables between stim and sham conditions in BC patients and stim condition in healthy controls. The relationship between circadian variables and both sleep parameters and cognitive scores as well as quality of life assessments will be assessed at any time using a linear mixed model (taking into account the correlation between repeated measures of the same subject). An alpha risk level of 5% (p < 0.05) will be retained for each analysis.

### 2.8. Data management

A web-based data capture system will be used for data collection and query handling. The investigator will ensure that data are recorded on an electronic case report form, as specified in the study protocol and in accordance with the instructions provided.

The investigator will ensure the accuracy, completeness, and timeliness of the data recorded and of the provision of answers to data queries according to the clinical study agreement. The investigator will sign the completed case report form. A copy of the completed form will be archived at the study site.

### 2.9. Withdrawal from study

The reasons for a participant’s withdrawal from the study will include the following circumstances:

- Intercurrent event not compatible with the pursuit of the study (e.g., start of treatment for sleep apnea or development of a neurological pathology);
- Participant’s decision (data already collected during the protocol can be kept and used unless participant opposes it);
- Loss of contact with participant;
- Investigator’s decision to exclude a participant.

### 2.10. Ethics and dissemination

The sponsor of this trial is the comprehensive Cancer Centre François Baclesse (Caen, France, b.clarisse@baclesse.unicancer.fr). This study received ethical approval from the Ile de France IV institutional review board on 19 April 2022 (no. ID-RCB: 2022-A00437-36). Eligible patients will be invited to participate by their medical and/or radiation oncologists, and eligible women free of disease will be invited to participate by the physicians from the U1077 research team. All eligible participants (patients and healthy women) will receive an information file. All participants will give their informed consent prior to undergoing any study-related assessment.

Data will be monitored by competent people. The findings resulting from this protocol will be presented at various conferences and published in peer-reviewed journals.

## 3. Discussion

A large proportion of patients with BC have circadian rhythm disruptions (particularly cortisol rhythm and RAR) that can last from the beginning of the disease to after the completion of treatment, and worsen with cancer stage [3, 10, 12, 17]. Alongside the circadian disruptions, patients frequently complain about both sleep and cognitive difficulties, from the point of their diagnosis to some years after they are cured of the disease [3, 12, 23, 91]. These feelings can heavily impact patients’ adherence to treatment and substantially reduce their quality of life [92–94]. Previous reports have demonstrated reductions in sleep quantity and quality among patients treated for BC [29, 95], mostly using actigraphy but also PSG [96, 97]. There is thus a need to tackle these difficulties using innovative approaches that are specifically designed to reduce RAR alterations [55].

These difficulties can be treated indirectly by targeting sleep disruption, either with medical drugs that may have negative effects or with APA and/or CBT, which may have a small effect on RAR or other circadian rhythm parameters, but not on sleep structure as measured with PSG, although CBT remains the first-line treatment for insomnia [45, 46, 49].

ICANSLEEP-2 is intended to provide proof of concept for the use of GVS to reduce circadian disruption among patients with BC. GVS could then be used in combination with other existing approaches (e.g., CBT and APA) to help limit circadian, sleep and cognitive impairments and enhance patients’ quality of life. Daily vestibular stimulation has been shown to accelerate the resynchronization of temperature circadian rhythms in rats after application of a 6-hr advance in the light/dark cycle [98]. Moreover, in humans, vestibular stimulation has already been proven to bring about a significant decrease in the mean activity level in the evening [66].

ICANSLEEP-2 has several potential limitations. First, there could be some minor side effects similar to motion sickness (e.g., nausea or dizziness) during stimulation. Nevertheless, the GVS device has already been tested in our laboratory in both young and older people, to collect preliminary data, and very few acute side effects were observed. Plus, any undesirable event will be reported to the medical investigator. Second, the sample size could be considered small. However, the aim of the present study is to provide proof of concept, so that GVS testing can then be extended to larger samples. Although it has been proven to be effective in improving RAR and sleep depth in healthy volunteers, vestibular stimulation has not yet been tested among patients with BC. GVS can be started immediately after treatment, and requires less input from healthcare professionals than CBT or APA, making it an easy approach to implement along the care pathway.

## 4. Conclusion

With ICANSLEEP-2, we expect to help patients with BC re-synchronize their circadian rhythms in order to alleviate their sleep complaints. We also expect to improve cognition, notably spatial memory.

If GVS does indeed help to improve these parameters, we expect this approach to be used more widely, complementing other methods that are already available, such as APA and CBT. It will be an efficient means of improving patients’ quality of life and course of care.

## Supporting information

S1 Checklist SPIRIT 2013 checklist: Recommended items to address in a clinical trial protocol and related documents*. (PDF)

S2 Checklist Human participants research checklist. (PDF)

S1 File (PDF)

S2 File (PDF)

## Abbreviations

APA: adapted physical activity
BC: breast cancer
CBT: cognitive-behavioral therapy
GVS: galvanic vestibular stimulation
PSG: polysomnography
RAR: rest-activity rhythm
