# Supplementary material for: Exploration of effects of galvanic vestibular stimulation on circadian rhythms and its associations with sleep and spatial memory in patients with breast cancer: The ICANSLEEP-2 protocol
Source: PLoS One. 2024 Jul 31;19(7):e0306462. doi: 10.1371/journal.pone.0306462 (PMC11290633; doi:10.1371/journal.pone.0306462)
Supplement: S1 File — (PDF) [file pone.0306462.s003.pdf]

ICANSLEEP PROTOCOL

IMPACT OF SLEEP DISTURBANCE ON COGNITION AND QUALITY OF LIFE IN BREAST CANCER FOLLOWING CHEMOTHERAPY

ICANSLEEP

ID-RCB N°: 2022-A00437-36

Version n° 2.1 dated from 05-SEPT-2023

This trial is supported by the NORMANDIE Region, as part of a "RIN [Recherche et Innovation " Recherche 2020 " Chaire d'Excellence" and by doctoral funding from doctoral school N°556 - HSRT (HOMME, SOCIÉTÉS, RISQUES, TERRITOIRE).

**TEST CLASSIFICATION: RIPH category 2**

**SPONSOR**	**Centre François Baclesse**<br>3 avenue du Général Harris<br>14076 CAEN cedex 5 - FRANCE

**COORDONNATOR INVESTIGATOR**	**Dr Carine SEGURA-DJEZZAR**<br><br>Centre François Baclesse<br>3 avenue du Général Harris<br>14076 CAEN cedex 5 - FRANCE

**ETHIC COMMITTEE**	CPP IDF IV	Approval date: 04/19/2022

PEOPLE INVOLVED IN PREPARING AND CONDUCTING THE RESEARCH

PARTICIPATING CENTERS	
**INVESTIGATORS**	**RESEARCH LOCATION**
**COORDINATING INVESTIGATOR**<br>Dr Carine SEGURA-DJEZZAR<br><br>**CO-INVESTIGATORS - PATIENT RECRUITMENT**<br>Dr Christelle LEVY<br>Dr George EMILE<br>Dr Djelila ALLOUACHE<br>Dr Adeline MOREL	Center François Baclesse – CAEN, FRANCE
**CO-INVESTIGATORS – RECRUITMENT of WITNESSES**<br>Prof. Fausto VIADER<br>Prof. Vincent DE LA SAYETTE<br>Prof. Olivier MARTINAUD<br>Prof. Pierre Denise	INSERM-EPHE-UNICAEN U1077 - CAEN, FRANCE
**PEOPLE INVOLVED IN THE PROJECT**	
**SCIENTIFIC MANAGER**<br>Prof. Bénédicte GIFFARD	**UMR_S 1077 Inserm-EPHE-Normandie University**, CAEN, FRANCE

**U1077 RESEARCH TEAM**<br>⇨ **PFRS**<br>Joy PERRIER (Post-doctoral researcher)<br>Clara Elia (PhD student)<br>Francis EUSTACHE (DE)<br>Laura DE GIROLAMO (IE)<br>Patrice CLOCHON (IR1)<br>Franck DOIDY (AI)<br><br>⇨ **Cyceron**<br>Mikaël NAVEAU (IE)<br>Nicolas DELCROIX (IR)	Health Training and Research Center (PFRS)<br>2 rue des Rochambelles CS 14032<br>F-14032-Caen cedex, FRANCE<br><br>**GIP Cyceron-** Bd Becquerel - BP 5229<br>14074 CAEN cedex 5
**COLLABORATING RESEARCH TEAMS**<br>Gaëlle QUARCK (PU) - U1075, Caen<br>Olivier ETARD (PH) - U1075, Caen<br><br>Tristan Martin (MCU) - EA 4334, Le Mans<br><br>Hélène Castel (DR) - U1239, Rouen	**UMR-S 1075 - UNICAEN-INSERM**<br>Mobility: Aging, Pathology, Health - COMETE<br><br>**EA 4334, University of Le Mans**, FRANCE<br>Movement, Interaction, Performance - MIP<br>**U1239, INSERM, University of Rouen**, FRANCE<br>Neuronal and Neuroendocrine Differentiation and Communication - DC2N
**CLINICAL RESEARCH**<br><br>**PROMOTING RESEARCH**<br>Bénédicte CLARISSE**, Promotion Manager**<br>Jean-Michel GRELLARD, **Project Manager**<br>Justine LEQUESNE**, Methodologist**	Center François Baclesse - CAEN, FRANCE<br><br>**DRCI, Clinical Research Department**

TABLE OF CONTENTS

1 SYNOPSIS 6

2 LIST OF ABBREVIATIONS 9

3 STUDY DIAGRAM 10

4 SUMMARY OF TESTS TO BE PERFORMED 11

5 SCIENTIFIC JUSTIFICATION FOR THE STUDY 13

5.1 CURRENT STATE OF KNOWLEDGE 13

5.2 EXISTING TREATMENTS AND THEIR LIMITATIONS 15

5.3 DESCRIPTION OF PROPOSED NEW MANAGEMENT 15

6 STUDY OBJECTIVES 16

6.1 MAIN OBJECTIVE 16

6.2 SECONDARY OBJECTIVES 16

7 RESEARCH HYPOTHESIS AND EXPECTED RESULTS 16

8 JUDGING CRITERIA 17

8.1 MAIN CRITERION 17

8.2 SECONDARY CRITERIA 19

9 STUDY OUTLINE 20

9.1 METHODOLOGY 20

9.2 DURATION OF STUDY 20

9.3 SELECTION OF PARTICIPANTS 20

9.3.1 Patient inclusion criteria 20

9.3.2 Criteria for non-inclusion of patients 20

9.3.3 Selection criteria for cancer-free women 21

9.4 COURSE OF THE STUDY 21

9.4.1 Inclusion procedure 21

9.4.2 Study evaluations 22

9.4.3 Inclusion report 23

9.4.4 T1 and T2 balances 24

9.4.5 Balance sheet T3 25

9.5 PREMATURE STUDY TERMINATION 26

9.6 METHODS FOR CARRYING OUT PARTICIPANT EVALUATIONS 26

10 EVALUATION TOOLS 26

10.1 QUALITY AND PHYSIOLOGY OF SLEEP AND CIRCADIAN RHYTHMS 26

10.1.1 Self-assessment of sleep and circadian typology 27

10.1.2 Sleep/wake cycle test 27

10.1.3 Examination of nocturnal sleep 27

10.1.4 Physiological measurements related to circadian rhythms and stress response 27

10.2 FUNCTIONAL CONNECTIVITY AND CORTICAL STRUCTURES 28
10.2.1 MRIa 29
10.2.2 fMRI 30
10.3 NEUROPSYCHOLOGICAL TESTS 30
10.3.1 Overall cognitive efficiency 30
10.3.2 Spatial memory task 30
10.3.3 Attentional Network Test (ANT) 32
10.3.4 Classical neuropsychological tests 33
10.4 QUALITY OF LIFE 35
11 GALVANIC VESTIBULAR STIMULATION (GVS) 38
11.1 TECHNICAL DESCRIPTION 38
11.2 CONTRAINDICATIONS AND EXPECTED SIDE EFFECTS 39
12 REGULATORY HEALTH VIGILANCE 39
13 STATISTICAL CONSIDERATIONS 40
13.1 NUMBER OF SUBJECTS REQUIRED 40
13.2 STATISTICAL ANALYSIS 40
13.3 NON-COMPLIANCE WITH PROTOCOL 41
14 QUALITY ASSURANCE 41
15 ETHICAL AND REGULATORY CONSIDERATIONS 41
15.1 REGULATORY APPROVALS 41
15.2 PARTICIPANT INFORMATION AND WRITTEN INFORMED CONSENT FORM 42
15.3 STUDY CONDUCT AND INVESTIGATOR RESPONSIBILITIES 42
15.4 DATA OWNERSHIP AND CONFIDENTIALITY 43
16 DATA PROCESSING AND STORAGE 43
16.1 DATA COLLECTION AND PROCESSING 43
16.2 ARCHIVING 43
16.3 DATA PROPERTIES AND PUBLICATION RULES 44
17 FINANCING AND INSURANCE 44
17.1 STUDY BUDGET 44
17.2 INSURANCE 44
18 BIBLIOGRAPHICAL REFERENCES 45
19 APPENDICES .... ERREUR ! SIGNET NON DEFINI.

1 SYNOPSIS

**TITLE**	**Impact of sleep disturbance on cognition and quality of life in breast cancer**
**ACRONYM**	**ICANSLEEP**
**Coordinator**	Dr Carine SEGURA-DJEZZAR
**Indication**	Patients with operated localized breast cancer who may or may not require adjuvant chemotherapy<br>Women with no history of cancer (control group)
**Design**	Bicentric, longitudinal trial (evaluations before and after chemotherapy, comparison with control group; and comparison before and after vestibular stimulation)
**Objectives**	**Main objectives**<br>**Axis 1:** To characterize the sleep of patients undergoing breast cancer treatment before and after adjuvant chemotherapy, compared with patients not treated with chemotherapy and with women free from history of cancer.<br>**Axis 2:** To explore the regulatory effect of galvanic vestibular stimulation (GVS) on circadian rhythms in patients undergoing treatment for localized breast cancer, compared with SHAM stimulation (not effective).<br>**Secondary objectives**<br>**Axis 1:**<br>**1)** **a)** To assess the effects of cancer and chemotherapy on sleep and circadian rhythms<br>**b)** To determine whether the presence of sleep disturbances is associated with altered circadian rhythms<br>**2)** To assess the effects of cancer and chemotherapy on cognitive abilities and their neuro-functional correlates<br>**3)** To assess the effects of cancer and chemotherapy on cortical structure<br>**4)** Assess the effects of cancer and chemotherapy on functional connectivity at rest<br>**5)** To evaluate the link between sleep disturbances and:<br>**a)** Cognitive abilities and their neuroanatomical correlates<br>**b)** The stress-related physiological systems<br>**c)** Quality of life<br>**Axis 2:**<br>**1)** To evaluate the effects of GVS on:<br>**a)** Circadian rhythms<br>**b)** Spatial memory<br>**c)** The stress-related physiological systems<br>**2)** To assess the effects of rhythm resynchronization on sleep quality<br>**3)** To determine whether improved sleep is accompanied by better cognitive performance and quality of life
**Judgement criteria**	**Main criterion**<br>**Axis 1:** sleep efficiency and number of awakenings (polysomnography) and **Axis 2:** amplitude and acrophase of activity-rest rhythm (actigraphy)

	**Secondary criteria**<br>**Axis 1:**<br>**1) a) b)** Actigraphy (rhythm amplitude and acrophase); Sleep diary (scores), Diurnal cortisol (cortisol levels); Polysomnography (sleep efficiency and wakefulness numbers); ISI, PSQI questionnaires (scores)<br>**2)** Neuropsychological tests (scores); Spatial memory task (scores); fMRI activation - ANT task (task-related BOLD signal - effective connectivity)<br>**3)** aMRI (cortical structures - white and gray matter)<br>**4)** fMRI at rest (dynamic fluctuations of the BOLD signal - functional connectivity, corrected for cardiorespiratory confounding factors)**;** Post-MRI debriefing questionnaire (scores)<br>**5) a)** Polysomnography (sleep efficiency and number of awakenings); ISI, PSQI questionnaires (scores); HVLT (scores); Spatial memory task (scores); aMRI: (cortical structures - white matter); STAI, BDI-II questionnaires (scores)<br>**5) b)** Polysomnography (sleep efficiency and number of awakenings); ISI, PSQI questionnaires (scores); CAR and pre-post MRI cortisol (cortisol levels)<br>**5) c)** Polysomnography (sleep efficiency and number of awakenings); ISI, PSQI questionnaires (scores); Quality of life questionnaires (scores)<br>**Axis 2:**<br>**1) a)** Actigraphy (rhythm amplitude and acrophase); Sleep diary (scores); Diurnal cortisol (cortisol levels)<br>**1) b)** Spatial memory task (scores)<br>**1) c)** CAR and pre-post MRI cortisol (cortisol levels)<br>**2)** Actigraphy (rhythm amplitude and acrophase), Sleep diary: (scores); Polysomnography (sleep efficiency and number of awakenings); ISI, PSQI questionnaires (scores)<br>**3)** Polysomnography (sleep efficiency and number of awakenings); ISI, PSQI questionnaires (scores); Neuropsychological tests (scores); Quality of life questionnaires (scores)
**Inclusion criteria (patients)**	- Patient aged between 45 to 65 years old<br>- Patient with localized breast cancer, whether or not to start adjuvant chemotherapy<br>- Patient with education level 3 "end of primary education" (Barbizet scale)<br>- Patient fluent in French<br>- Patient with Internet access from home computer<br>- Patient having signed consent to participate in the study<br>- Patient with sleep complaints (ISI > 7)<br>- Patient with home computer access to the Internet
**Non-inclusion criteria (patients)**	- Patients with metastatic cancer<br>- Patient with a primary cancer other than breast cancer<br>- Patients with a history of neurological damage<br>- Patient with treated sleep apnea<br>- Patient with drug or alcohol abuse (≥3 drinks/day on average and/or >10 drinks/week)<br>- Patient with treatment that has not been stabilized for at least 3 months (hypnotics, antidepressants, anxiolytics)<br>- Patient with a personality disorder and/or progressive psychiatric pathology<br>- Patients with contraindications to MRI (claustrophobia, metal objects in the body)<br>- Patient with uncorrected vision problem<br>- Patients with cognitive impairment pre-existing cancer diagnosis

**Selection criteria for healthy volunteers**	- Female from general population matched for age and education (Barbizet scale)<br>- Female, minimum education level 3 "end of primary education" (Barbizet scale)<br>- French-speaking woman<br>- Women with access to the Internet from a home computer<br>- Woman who signed consent to participate in the study<br>- Women with no history of cancer<br>- Women with sleep complaints (ISI > 7)<br>- Women with access to the Internet from a home computer<br>- Women who do not work shifts<br>- Women with no history of neurological damage<br>- Women with untreated sleep apnea<br>- Women with no drug use or alcohol abuse (≥3 drinks/day on average and/or >10 drinks/week)<br>- Women with no treatment that has not been stabilized for at least 3 months (hypnotics, antidepressants, anxiolytics)<br>- Women with no personality disorder or progressive psychiatric pathology<br>- Women with no contraindications to MRI (claustrophobia, metal objects in the body)<br>- Women with no uncorrected vision problems
**Description of protocol / experimental design**	Participants (2 subgroups of patients and 1 group of cancer-free, female volunteers of the same age) undergoing 3 successive assessments (T1: baseline, T2: at 6 months, T3: after galvanic vestibular stimulation).<br>Assessments include:<br>- aMRI and fMRI at rest and in activation with the ANT task (only at T1 and T2)<br>- Biological and physiological measurements<br>- Spatial memory task<br>- Cognitive battery<br>- Questionnaires (sleep, quality of life)
**Number of patients required**	• 25 patients treated with chemotherapy for operated localized breast cancer<br>• 25 patients not treated with chemotherapy for operated localized breast cancer<br>• 25 breast cancer-free female volunteers of the same age<br>A total of 75 participants, including 50 patients and 25 breast cancer-free women
**Participating centers**	Center François Baclesse (patient recruitment), Caen, France<br>Unit 1077 (recruitment of healthy subjects), Caen, France
**Study duration**	48 months

2 LIST OF ABBREVIATIONS

ANT: Attentional Network Test

BDI: Beck Depression Inventory

Brief COPE: Brief Coping Orientation to Problems Experienced

BPI: Brief Pain Inventory

CAR: Cortisol Awakening Response

FACIT-F: Functional Assessment of Chronic Illness Therapy - Fatigue

FACT-Cog: Functional Assessment of Cancer Therapy - Cognitive Function

FACT-G: The Functional Assessment of Cancer Therapy - General

GVS: Galvanic Vestibular Stimulation

HVLT: Hopkins Verbal Learning Test

IPAQ: International Physical Activity Questionnaire

ISI: Insomnia Severity Index

KSS: Karolinska Sleepiness Scale

MDC: Memory for numbers

MFI-20: Multidimentional Fatigue Inventory

MoCA: Montreal Cognitive Assessment

Oth: Comments from Others

PCA: Perceived Cognitive Abilities

PCI: Perceived Cognitive Impairment

PCL-5: Posttraumatic Stress Disorder Checklist for DSM-5 (Diagnostic and Statistical Manuel of mental disorders, 5th version)

PSQI: Pittsburgh Sleep Quality Index

PTGI: Post-Traumatic Growth Inventory

QoL: Quality of Life

STAI-Y: State-Trait Anxiety Inventory

TMT: Trail Making Test

vm: Virtual Meter

3 STUDY DIAGRAM

<u>Patients treated with adjuvant chemotherapy ***(CHI group)*** :</u>

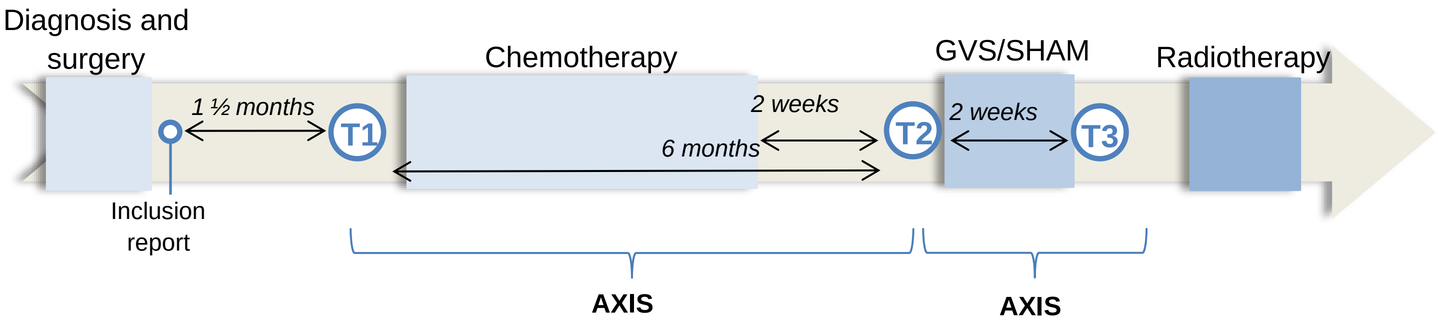

<u>Patients not treated with adjuvant chemotherapy ***(NCH group)*** :</u>

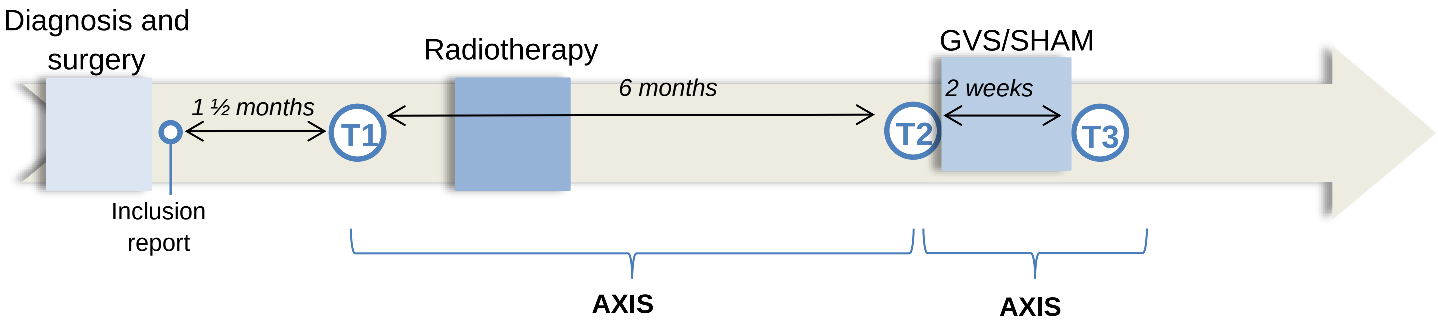

<u>Cancer-free female volunteers ***(CTL group)*** :</u>

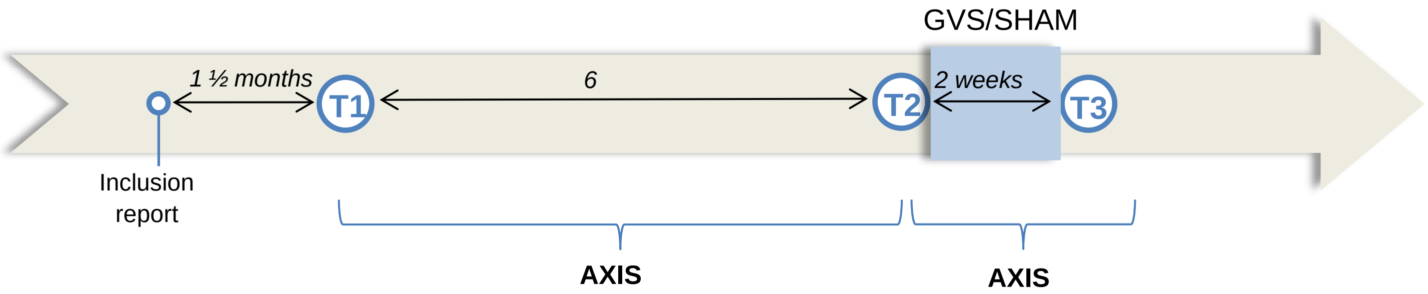

Figure 1. Schematic diagram of the study by group

Note. *GVS: Galvanic Vestibular Stimulation, SHAM, non-effective stimulation*

4 SUMMARY OF TESTS TO BE PERFORMED

Table 1 Summary of assessments offered to participants at different stages of the study

**Time**<br>**Assessments**	**Inclusion**	**T1**<br>Within one month of inclusion and before the start of treatment (chemotherapy or radiotherapy)	**T2**<br>6 months after T1	**T3**<br>2 weeks after T2
Signature of informed consent	•			
Sleep complaints and sleep apnea (questionnaires) :<br>- ISI<br>- Berlin Questionnaire	•			
Quality and physiology of sleep and circadian rhythms<br>- Questionnaires (home): ISI, PSQI, Horne and Ostberg<br>- Actigraphy and sleep diary *(over the 2 weeks preceding each evaluation period T1, T2 and T3)*<br>- Polysomnography<br>- Diurnal salivary cortisol<br>- CAR and cortisol pre-post MRI		•	•	•
Functional connectivity and cortical structures<br>- aMRI<br>- Resting fMRI<br>- Activation fMRI with attentional task (ANT)<br>- Physiology associated with MRI (respiration, plethysmography)<br>- Post-MRI debriefing questionnaire		•	•	
Spatial memory task		•	•	•
Global cognitive ability: MoCA		•		
Cognitive battery<br>- HVLT<br>- Space Memory Subtest (MEM-III)<br>- Baddeley's double task<br>- N-Back<br>- d2-R<br>- TMT A and B<br>- Spelling fluency		•	•	•

- Stroop				
Quality of life (questionnaires)<br>- FACT-G (home, patients only)<br>- FACIT-F (home, patients only)<br>- MFI-20 (home)<br>- FACT-Cog (home)<br>- KSS<br>- BPI<br>- IPAQ<br>- BDI-II<br>- STAI-Y - status and features<br>- PCL-5 (patients only)<br>- PSS<br>- Brief COPE - modified dispositional "before cancer diagnostic" (patients only, T1 only) and dispositional<br>- PTGI (patients only)		•	•	•

Note. *ISI: Insomnia Severity Index; CAR: Cortisol Awakening Response; ANT: Attentional Network Test; MoCA: Montreal Cognitive Assessment; HVLT: Hopkins Verbal Learning Test; MEM-III: Wechsler Clinical Memory Scale - 3ème edition; TMT : Trail Making Test; PSQI: Pittsburgh Sleep Quality Index; FACT-G: Functional Assessment of Cancer Therapy - General; FACIT-F: Functional Assessment of Chronic Illness Therapy - Fatigue; MFI-20: Multidimensional Fatigue Inventory; FACT-Cog: Functional Assessment of Cancer Therapy - Cognitive Function; KSS: Karolinska Sleepiness Scale; BPI: Brief Pain Inventory; IPAQ: International Physical Activity Questionnaire; BDI-II: Beck Depression Inventory, 2nd edition ; STAI-Y: State-Trait Anxiety Inventory ; PSS: Perceived Stress Scale ; PCL-5: Posttraumatic Stress Disorder Checklist for DSM-5 ; Brief COPE: Brief Coping Orientation to Problems Experienced ; PTGI: Post-Traumatic Growth Index*

5 SCIENTIFIC CONTEXT OF THE STUDY

About 40% of patients treated for breast cancer frequently complain of sleep disorders. Insomnia is the most frequent one. The prevalence of insomnia complaints is higher in breast cancer patients than in other types of cancer, and is also higher than in the general population (between 20% and 70% in breast cancer patients *vs.* 30% in the general population) [1,2]. A recent study indicates that sleep complaints concern 25% of patients even before diagnosis, and 46% (including 18% complaining of insomnia) at the time of diagnosis, showing the negative impact of the announcement of the pathology on subjective sleep quality, and in particular on symptoms suggestive of insomnia [3]. However, objective evidence of sleep patterns's modifications in breast cancer remains scarce and inconclusive (see [4] for review). In particular, previous studies have focused only on the effects of chemotherapy and have not always included a control group, limiting the scope of their results. The links with cognitive and psychopathological processes, as well as the underlying mechanisms, are not clearly demonstrated in this pathology. Finally, taking into account patients' complaints, it seems necessary to limit sleep disorders in breast cancer in order to improve patients' quality of life using non-medicinal, easy-to-implement approaches.

5.1 CURRENT STATE OF KNOWLEDGE

Sleep difficulties in breast cancer remain poorly studied in a precise and integrated way (i.e. taking into account all the processes involved in sleep regulation). Indeed, sleep is intrinsically linked to circadian rhythms, with circadian rhythmicity in humans reflected by a spontaneous alternation of periods of sleep and activity over 24 hours, i.e. the activity-rest rhythm. Previous studies have shown deregulation of the activity-rest rhythm in breast cancer before and during chemotherapy [5,6] but also in patients not treated with chemotherapy [7]. Of these studies, only three, including one from our work, included a control group with no history of cancer [8,9]. The results have shown a longer total sleep time and a longer duration of naps during chemotherapy compared with the period before initiation of treatment [10–12] and in comparison with controls free of cancer[8,9]. In addition, the activity-rest rhythm appeared to be less ample in breast cancer patients who had not received chemotherapy, compared with subjects with no history of cancer [7]. Conversely, Li and colleagues showed that initiation of chemotherapy, compared with pre-treatment assessments and the end of chemotherapy, was associated with shorter total sleep time, as well as more frequent awakenings and poorer sleep quality [13]. Beck and colleagues also report a shorter total sleep time on the first night after chemotherapy initiation, compared with before and after the treatment period [14]. Finally, Kuo and colleagues found no difference between assessments before and during chemotherapy [15]. The results concerning the effects of chemotherapy on sleep parameters measured by actigraphy are therefore highly variable, and do not allow to draw definitive conclusions as to sleep alterations as measured by actigraphy [4]. A literature review published in 2015 [16] reported, however, that chemotherapy could accentuate sleep difficulties already present before the initiation of treatment in breast cancer.

In contrast to actigraphic studies, polysomnographic (PSG) studies, the *gold-standard* of sleep assessment, are still scarce. As a result, alterations in the structure of sleep in this pathology are not clearly described. In addition, PSG makes it possible to quantify parameters associated with sleep structure and memory consolidation. Its use therefore provides a better understanding of sleep changes than actigraphy, which remains an indirect approach to quantif

sleep quality and quantity. Previous PSG studies suggested alterations in sleep quality and quantity following chemotherapy treatment. Parker and colleagues compared the sleep architecture of patients treated for advanced non-cerebral cancer (stage 3 or 4 cancers), including 32 patients treated for breast cancer (28% of the cohort) [17]. The results have shown reduced sleep efficiency and sleep stage durations in these patients, compared with the norms established by Williams and colleagues in healthy participants [18]. Roscoe and colleagues [19] revealed that after the end of chemotherapy, patients slept more than before the start of chemotherapy. This result could be explained by poor sleep quality at baseline due to the stress associated with cancer diagnosis and the apprehension of chemotherapy, or by an accumulation of fatigue and sleep deprivation during treatment, leading to compensation at the end of treatment.

These studies have shown changes in the sleep's macrostructure in patients treated for breast cancer, but they have several limitations that prevent a precise understanding of the impact of cancer and treatment on sleep. The absence of a control group in several of these studies is the main limitation. Indeed, the presence of a control group with no history of cancer and a group without treatment makes it possible to determine whether changes in sleep architecture are secondary to the cancer itself or to treatment. In addition, the inclusion of heterogeneous populations in terms of age and cancer type may have limited the statistical power of the studies' results, and prevented changes in sleep architecture from being revealed. In addition, no studies have assessed changes in sleep patterns prior to initiation of chemotherapy. PSG also provides detailed information on the macro- and micro-structure of sleep, enabling links to be made with cognitive performance and the detection of potential sleep disorders such as apnea. New studies, more homogeneous in terms of eligibility criteria, but also longitudinal, are needed to determine the presence of sleep disorders in breast cancer patients. A better understanding of these sleep disturbances will also enable to determine their possible contribution to the onset of cognitive and psychopathological deficits.

Patients with breast cancer suffer not only from impaired episodic memory, but also from impaired executive functions. The latter are particularly involved in a particular form of episodic memory, spatial memory, which has so far received little attention in breast cancer. This type of memory enables to remember a route or find objects in different places, and is therefore essential for everyday life. What's more, it relies on the integrity of the hippocampus, a region that appears to be affected in women with breast cancer [8]. Sleep disturbances are also known to impair executive functions involving the prefrontal cortex [9] and the cognitive control mechanisms that depend on it. These cognitive control mechanisms enable the regulation of behavior in complex situations, particularly after a stressful event. In breast cancer, sleep disorders could have a negative impact on these control mechanisms, leading to reduced resistance to stress.

In addition to the beneficial effects of sleep on cognitive processes, particularly memory and executive functions [20,21], sleep plays an important role in the stress response in healthy subjects and in various pathologies [22]. Cancer diagnosis, as well as the situation associated with the pathology, constitute a major source of stress. However, as is the case in the general population, patients do not react homogeneously to these situations. Sleep disorders could influence the way patients react to stressful situations, such as those associated with their care pathway. There is a need to understand to what extent sleep influences psychopathological processes in breast cancer, so that it is possible to improve supportive care throughout cancer trajectory.

5.2 ALREADY EXISTING CARE METHODS AND THEIR LIMITATIONS

Sleep and circadian rhythm disturbances are common in breast cancer, and need to be managed. Cognitive-behavioural therapies and physical activity are currently proposed as non-medicinal approaches. However, although positive effects have been reported on mood, quality of life and sleep complaints, their beneficial effects on the quality and quantity of sleep measured by PSG in breast cancer are not clearly established [23,24]. Moreover, these approaches do not specifically target sleep and/or circadian rhythm disturbances. Promising results suggest that the vestibular system may be involved in circadian regulation and resynchronization [25–27]. Similarly, recent studies have shown the positive effects of vestibular system stimulation *via* sleep balancing on sleep quality in healthy subjects. Finally, recent results from the COMETE team (doctoral thesis by Florane Pasquier, Dir: Prof. Gaëlle Quarck) suggest that stimulation of the vestibular system, particularly galvanic stimulation, could have an effect on the circadian rhythms [28]. The use of vestibular system stimulation therefore appears particularly appropriate for specifically improving sleep and resynchronizing rhythms in breast cancer. This could be achieved using galvanic vestibular stimulation (GVS), based on stimulation of the peripheral vestibular organ through activation of vestibular afferents, by applying an electric current between the two mastoids. GVS has been used for many years to safely and painlessly stimulate the vestibular system [29] but more recent studies have also demonstrated the feasibility and positive effects of GVS on cognition [30–32] and anxiety levels [33] in healthy participants. GVS has no particular contraindications and could therefore be offered to all participants.

5.3 DESCRIPTION OF THE PROPOSED NEW TREATMENT

The GVS will be performed using a device available from U1075 (Caen, Dir: Prof. Thomas Freret), a team specializing in remediation related to biological rhythms, notably through vestibular stimulation. The device used is distributed by Soterix médical and is CE marked (https://soterixmedical.com/research/vestibular). This equipment enables vestibular afferents to be stimulated autonomously and in a pre-regulated way: the stimulation level is pre-programmed and cannot be modified without being the administrator of the device, ensuring precise and safe stimulation. In order to determine the most accurate and effective stimulation protocol possible, the device is currently being evaluated in our laboratory on cancer-free participants as part of a Master's 2 internship, and no side effects have been reported. In addition, the COMETE unit (Dir: Thomas Freret, work by Prof. Gaëlle Quarck) is currently carrying out a protocol (VELOCCE project NCT05030389) on elderly people with no pathologies. Similarly, no side effects were reported by participants. All these results demonstrate the safety, feasibility and ease of use of the Soterix GVS device to be used in the ICANSLEEP protocol. The stimulation protocol (effective stimulation, GVS, or non-effective stimulation, SHAM) will be carried out remotely, five times a week in the morning for two weeks, using a securely accessible videoconferencing platform available on the Université de Caen network. Participants will be able to connect *via* a computer at the times and on the days indicated by the experimenters. Once connected, they will be able to videoconference with a professional from the research team, who will check the correct position of the electrodes and note the participants' behavior. These videoconferences will also help to ensure that the conditions are consistent between sessions and between participants.

Florane Pasquier's recently defended thesis (supervised by Gaëlle Quarck) demonstrated the tolerance and beneficial effects of galvanic stimulation on anxiety and circadian rhythms.

Publications using Soterix brand GVS equipment have recently been published, attesting to its safety and feasibility of use [34,35].

5.4 MAIN OBJECTIVE

The project has two main aims, each with its own main objective.

Axis 1: To characterize the sleep of patients undergoing breast cancer treatment before and after adjuvant chemotherapy, compared with patients not treated with chemotherapy and with women free from history of cancer.

Axis 2: To explore the regulatory effect of galvanic vestibular stimulation (GVS) on circadian rhythms in patients undergoing treatment for localized breast cancer, compared with SHAM stimulation (not effective).

5.5 SECONDARY OBJECTIVES

Axis 1:

1) **a)** To assess the effects of cancer and chemotherapy on sleep and circadian rhythms
   b) To determine whether the presence of sleep disorders is associated with altered circadian rhythms
2) To assess the effects of cancer and chemotherapy on cognitive abilities and their neuro-functional correlates
3) To assess the effects of cancer and chemotherapy on cortical structure
4) To assess the effects of cancer and chemotherapy on functional connectivity at rest
5) To evaluate the link between sleep disorders and:
   a) Cognitive abilities and their neuroanatomical correlates
   b) The stress-related physiological systems
   c) Quality of life

Axis 2:

1) To evaluate the effects of GVS on:
   a) Circadian rhythms
   b) Spatial memory
   c) The stress-related physiological systems
2) To assess the effects of rhythm resynchronization on sleep quality
3) To determine whether improved sleep is accompanied by better cognitive performance and quality of life

6 RESEARCH HYPOTHESIS AND EXPECTED RESULTS

Axis 1:

1) **a)** Sleep and circadian rhythms would be altered in patients before chemotherapy compared with cancer-free subjects. Chemotherapy would exacerbate these effects compared with the no-chemotherapy group

   - For sleep, more awakenings lasting more than a minute and poorer sleep quality are expected.

- For circadian rhythms, the sleep/wake and cortisol rhythms are expected to be less ample.

b) Altered rhythms have deleterious effects on sleep quality

2) Cognitive abilities would be more impaired after than before chemotherapy, whereas no decline is expected in patients not treated with chemotherapy and in healthy subjects. More specifically, this decline is expected to affect:

- Spatial and episodic memory
- Working memory
- Executive and attentional functions
- Functional connectivity during the ANT (Attentional Network Test) task, particularly within the orientation and cognitive control networks

3) After chemotherapy, the cortical structure should be altered, particularly in the regions involved in the above-mentioned cognitive abilities (i.e., hippocampus, frontal regions, cerebellum) compared to before chemotherapy, and compared to patients not treated with chemotherapy and healthy subjects.

4) For both groups of patients, the dynamics of functional connectivity at rest prior to chemotherapy should be altered, with greater ruminations than in the healthy group.

5) **a)** Sleep disturbances (independently of the effects of anxiety and depression) are thought to be linked to cognitive abilities and their neuro-anatomical correlates, specifically:

- Episodic and spatial memory performance
- White matter structure and functional connectivity

b) Sleep disturbances are also associated with hyperactivity of physiological stress systems

c) Sleep disturbances are associated with a lower quality of life

Axis 2:

1) GVS would allow :

a) Resynchronizing circadian rhythms (i.e. acrophase) and amplifying the sleep/wake rhythm as the procedure progresses and the cortisol rhythm at the end of the procedure.

b) Improving spatial memory performance compared to T2

c) Reducing hyperactivity of stress-related physiological systems

2) Resynchronizing rhythms improves sleep quality

3) This improvement in sleep quality is associated with better cognitive recovery and quality of life following the course of treatment, compared with the absence of GVS.

7 JUDGING CRITERIA

7.1 MAIN CRITERION

Axis 1: The primary endpoint is sleep efficiency (ratio of total sleep time to time spent in bed) and the number of awakenings lasting more than one minute. These data are derived from polysomnography.

Axis 2: The main criterion selected are amplitude (corresponding to half the maximum variation in the rhythm under consideration) and acrophase (i.e. the time of day when activity or cortisol is highest). Changes in sleep/wake rhythms will be quantified over the 2-week

intervention period, and will be derived from actigraphy. Changes in cortisol rhythm will be measured using saliva samples obtained at T2 and T3.

7.2 SECONDARY CRITERIA

Table 2 Secondary criteria associated with secondary objectives

Axis 1	
**Objectives**	**Examinations:** associated measures
**1) a) b)**	- **Actigraphy:** rhythm amplitude and acrophase<br>- **Sleep diary:** scores<br>- **Diurnal cortisol:** salivary cortisol levels<br>- **Polysomnography: sleep efficiency and number of awakenings**<br>- **ISI, PSQI questionnaires: scores**
**2)**	- **Neuropsychological tests:** scores<br>- **Spatial memory task:** scores<br>- **fMRI activation (ANT task):** task-related BOLD signal (effective connectivity)
**3)**	- **aMRI:** cortical structures (white and gray matter)
**4)**	- **Resting fMRI:** dynamic BOLD signal fluctuations (functional connectivity) corrected for cardiorespiratory confounding factors<br>- **Post-MRI debriefing questionnaire:** scores
**5) a)**	- **Polysomnography:** sleep efficiency and number of awakenings<br>- **ISI, PSQI questionnaires:** scores<br>- **HVLT:** scores<br>- **Spatial memory task:** scores<br>- **MRIA:** cortical structures (white matter)<br>- **STAI, BDI-II questionnaires:** scores
**5) b)**	- **Polysomnography:** sleep efficiency and number of awakenings<br>- **ISI, PSQI questionnaires:** scores<br>- **CAR and cortisol pre- and post-MRI:** salivary cortisol levels
**5) c)**	- **Polysomnography:** sleep efficiency and number of awakenings<br>- **ISI, PSQI questionnaires:** scores<br>- **Quality of life questionnaires:** scores
**Axis 2**	
**Objectives**	**Examinations:** associated measures
**1) a)**	- **Actigraphy:** rhythm amplitude and acrophase<br>- **Sleep diary:** scores<br>- **Diurnal cortisol:** salivary cortisol levels
**1) b)**	- **Spatial memory task:** scores
**1) c)**	- **CAR and cortisol pre- and post-MRI:** salivary cortisol levels
**2)**	- **Actigraphy:** rhythm amplitude and acrophase<br>- **Sleep diary:** scores<br>- **Polysomnography:** sleep efficiency and number of awakenings<br>- **ISI, PSQI questionnaires:** scores
**3)**	- **Polysomnography:** sleep efficiency and number of awakenings<br>- **ISI, PSQI questionnaires:** scores<br>- **Neuropsychological tests:** scores<br>- **Quality of life questionnaires:** scores

Note. *ISI: Insomnia Severity Index; PSQI: Pittsburgh Sleep Quality Index; ANT: Attentional Network Test; HVLT: Hopkins Verbal Learning Test; CAR: Cortisol Awakening Response; BDI-II: Beck Depression Inventory; STAI: State-Trait Anxiety Inventory.*

8 STUDY PLAN

8.1 METHODOLOGY

The ICANSLEEP project is a bicentric longitudinal study.

Data from 25 patients with localized breast cancer treated with chemotherapy will be compared with those from a group of 25 patients not treated with chemotherapy and a group of 25 cancer-free volunteers (control groups), matched for sex, age and years of education (Barbizet scale).

The various evaluations will be carried out as follows:

- At T1: Approximately 1.5 months after surgery and before the start of chemotherapy in the CHI group and before the start of radiotherapy in the NCH group,
- At T2: after a delay of around 6 months, within 2 weeks of the end of chemotherapy for the CHI group, or at a distance from radiotherapy for the NCH group,
- At T3: after a two-week intervention with GVS versus non-effective stimulation (known as SHAM). To assess the effect of GVS, patients will be assigned to either the effective GVS group or the SHAM group (random selection). SHAM stimulation involves using the device but receiving a very low-frequency current that has no effect on the vestibular system. This approach has already been used and validated in the COMETE unit (Caen, Prof. Gaëlle Quarck) with which we collaborate. All cancer-free volunteer participants will undergo GVS.

8.2 DURATION OF STUDY

The total duration of the study is 48 months, including a 40-month recruitment period.

The follow-up period for participants is 8 months from the inclusion assessment.

8.3 SELECTION OF PARTICIPANTS

For each of the two groups, patients (25 chemotherapy-treated patients and 25 non-chemotherapy-treated patients) will be recruited from the François Baclesse Cancer Center in Caen. Patients not treated with chemotherapy will be of equivalent age and level of education to patients treated with chemotherapy.

A group of 25 female volunteers with no history of cancer, of the same age and level of education as the patients, will be recruited through a call for volunteers (see "Inclusion of cancer-free women" below). Recruitment will take place within Unit 1077 (Caen, France).

8.3.1 Patient inclusion criteria

- Patient aged 45 to 65
- Patient with localized breast cancer, whether or not to start adjuvant chemotherapy
- Patient with education level 3 "end of primary education" (Barbizet scale)
- Patient fluent in French
- Patient having signed consent to participate in the study
- Patient with sleep complaints (ISI > 7)
- Patient with access to the Internet from a home computer

8.3.2 Criteria for non-inclusion of patients

- Patients with metastatic cancer

- Patient with a primary cancer other than breast cancer
- Patients with a history of neurological damage
- Patient with treated sleep apnea syndrome
- Patient with drug or alcohol abuse (≥3 drinks/day on average and/or >10 drinks/week)
- Patient with treatment that has not been stabilized for at least 3 months (hypnotics, antidepressants, anxiolytics)
- Patient with a personality disorder or progressive psychiatric pathology
- Patients with contraindications to MRI (claustrophobia; metallic objects in the body such as pacemakers, implanted equipment activated by an electrical, magnetic or mechanical system, hemostatic clips for intracerebral aneurysms or carotid arteries, orthopedic implants)
- Patient with uncorrected vision problem
- Patients with pre-existing cognitive impairment at the time of cancer diagnosis

8.3.3 Selection criteria for cancer-free women

- Female from general population matched for age and education (Barbizet scale) to female patients
- Female, minimum education level 3 "end of primary education" (Barbizet scale)
- French-speaking woman
- Woman who signed consent to participate in the study
- Woman with no history of cancer
- Woman with sleep complaints (ISI > 7)
- Woman without treated sleep apnea
- Woman with access to the Internet from a computer at home
- Woman who does not work shifts
- Woman with no history of neurological damage
- Woman with no drug use or alcohol abuse (≤3 drinks/day on average and/or <10 drinks/week)
- Woman with no treatment that has not been stabilized for at least 3 months (hypnotics, antidepressants, anxiolytics)
- Woman with no personality disorder or progressive psychiatric pathology
- Woman with no contraindications to MRI (claustrophobia; metal objects in the body such as pacemakers, implanted equipment activated by an electrical, magnetic or mechanical system, hemostatic clips for intracerebral aneurysms or carotid arteries, orthopedic implants).
- Woman with no uncorrected vision problems

8.4 COURSE OF THE STUDY

8.4.1 Inclusion procedure

8.4.1.1 Patient inclusion

The study will be proposed by oncologists and/or radiotherapists to patients meeting the eligibility criteria (groups with and without adjuvant chemotherapy). Patients will be given an information note and an informed consent form. They will have a cooling-off period of the duration of their choice.

Once the patient's agreement has been obtained by signing the study consent form, the selection criteria will be verified before inclusion in the trial.

Study-specific examinations requested prior to inclusion (inclusion work-up) will be carried out after consent has been signed and prior to inclusion.

Inclusion will be recorded on the study software via an Internet portal.

An identification number will be assigned to the patient and used throughout the study.

8.4.1.2 Inclusion of cancer-free women

Healthy volunteers will be recruited through acquaintances, associations, the ‘’Espace Rencontre et Information [ERI]’’ at the Centre François Baclesse, or other groups of individuals. After prior phone contact and a brief presentation of the study, healthy volunteers will be invited to our research unit for the inclusion visit. During this visit, the investigator will present the purpose and modalities of the study, and then give the volunteers an information leaflet and a consent form. Control participants will be given a period of reflection of their choice to decide whether or not to participate in the protocol.

Cancer-free women will be matched in age and education (Barbizet scale) to patients.

After written agreement from the subject, verification of all selection criteria, and before starting the study, inclusion will be recorded on the study software via an Internet portal.

An identification number will be assigned to the volunteer woman, to be used throughout the study.

8.4.2 Study evaluations

A table summarizing the investigations requested and a diagram showing the different stages in the evaluation of participants are included at the beginning of the document (*Figure 1*).

The first evaluation will be carried out within one month of inclusion and before the start of chemotherapy (CHI group) or radiotherapy (NCH group), depending on the availability of participants.

Throughout the evaluation sessions, participants will be accompanied by the UMR 1077 team, comprising a neuropsychologist, a sleep technician and a PhD student.

Prior to the assessment sessions, actigraphy data will be collected from participants 2 weeks before each assessment session (T1, T2 and T3). For this purpose, an actigraph and a sleep diary with explanations on how to use them will be provided to participants either *in person,* or remotely (actigraph and sleep diary sent by post and videoconference).

The assessments proposed to the participants during the different study phases (T1, T2 and T3) are presented in the following figures (*Figure 2* and *Figure 3*). Each evaluation sequence will be carried out over 2 half-days (afternoon and morning), separated by a night's sleep to allow polysomnography examination.

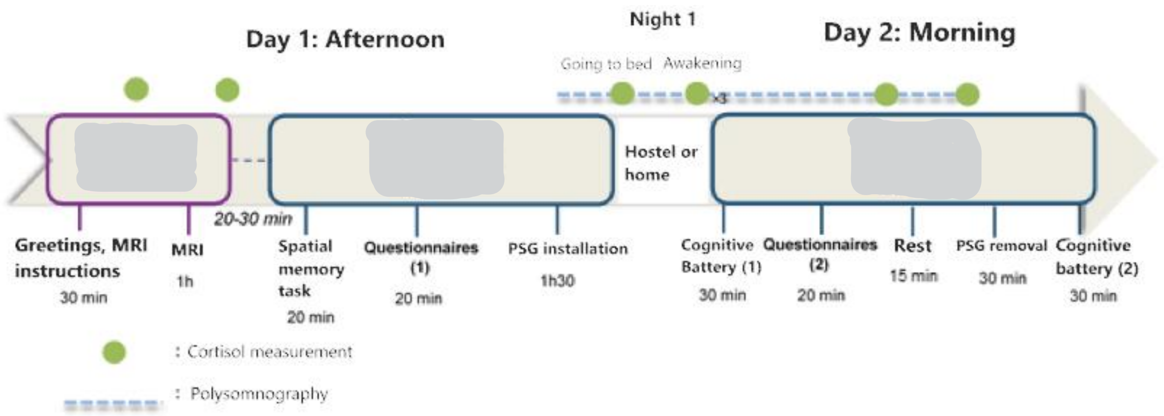

Figure 2. Assessments offered to participants at T1 and T2

Note. *Actigraphy will be provided 2 weeks before T1, idem at T2; At T1, a preliminary interview will take place at the reception of the participants (approximately 30 min, in addition to the MRI instructions); At T1, a period of familiarization with the galvanic vestibular stimulation equipment will take place before the MRI examination (20 min); PSG:*

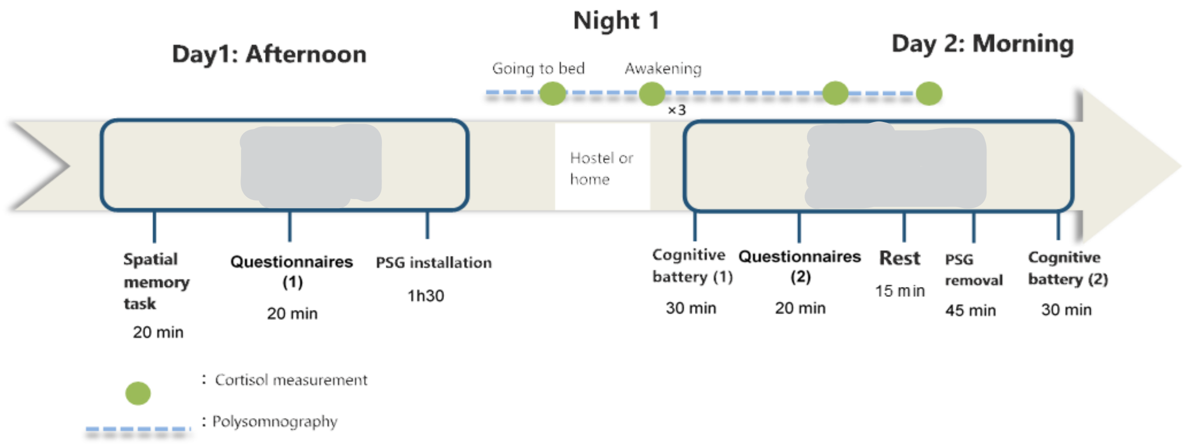

Figure 3. Assessments offered to participants at T3

Note. *Actigraphy will be provided 2 weeks before T3; PSG: Polysomnography*

8.4.3 Inclusion report

Participants who have signed their consent to participate will be asked to perform the following tests**:**

- ISI (sleep complaints)
- Berlin questionnaire (sleep apnea)

8.4.4 T1 and T2 reports

In patients treated with adjuvant chemotherapy (CHI group)

- The T1 assessment will take place after surgery and before the start of chemotherapy.
- The T2 assessment will take place 2 weeks after the end of the last session of chemotherapy.

In patients not treated with adjuvant chemotherapy (NCH group)

- The T1 assessment will take place after surgery and before the start of radiotherapy.
- T2 assessment will take place 6 months after T1

In healthy volunteer participants (CTL group):

- T1 assessment will take place within 6 weeks of inclusion assessment
- T2 assessment will take place 6 months after T1

For all study participants, the following assessments will be proposed at T1 and T2:

- Actigraphy and sleep diary (for 2 weeks before each check-up)
- Questionnaires to be completed at home (sleep and quality of life):
    - ISI
    - PSQI
    - Horne and Ostberg
    - FACT-G (patients only)
    - FACIT-F (patients only)
    - MFI-20
    - FACT-Cog
- Diurnal salivary cortisol and CAR (Cortisol Awakening Response) assays
- MRI (aMRI and fMRI at rest and activation with ANT task + post-MRI debriefing questionnaire)
- Spatial memory task
- Polysomnography
- Neuropsychological tests :
    - MoCA (T1 only)
    - HVLT
    - Space Memory Subtest (MEM-III)
    - Baddeley's double task
    - N-Back
    - d2-R
    - TMT A and B
    - Spelling fluency
    - Stroop
- Questionnaires (quality of life) :
    - KSS
    - BDI-II
    - BPI

    - STAI - State
    - STAI - Trait
    - PCL-5 (patients only)
    - PSS
    - Modified dispositional brief-COPE (before cancer diagnosis) (patients only, T1 only)
    - Dispositional COPE Brief
    - PTGI
    - IPAQ

-Assessment of vestibular function (video Head Impulse Test, vHIT)

8.4.5 Balance sheet T3

For all participants, the following assessment will be carried out at the end of the GVS or SHAM period (2 weeks) after T2.

- Actigraphy and sleep diary (between T2 and T3)
- Questionnaires to be completed at home (sleep and quality of life):
    - ISI
    - PSQI
    - Horne and Ostberg
    - FACT-G (patients only)
    - FACIT-F (patients only)
    - MFI-20
    - FACT-Cog
- Dosing of diurnal salivary cortisol and CAR (Cortisol Awakening Response)
- Spatial memory task
- Polysomnography
- Neuropsychological tests :
    - HVLT
    - Space Memory Subtest (MEM-III)
    - Baddeley's double task
    - N-Back
    - d2-R
    - TMT A and B
    - Spelling fluency
    - Stroop
- Questionnaires (quality of life) :
    - KSS
    - BDI-II
    - BPI
    - STAI - State
    - STAI - Trait
    - PCL-5 (patients only)
    - PSS
    - Dispositional COPE Brief

- PTGI
  IPAQ

-Assessment of vestibular function (video Head Impulse Test, vHIT)

8.5 PREMATURE STUDY TERMINATION

The intervention will be interrupted at any time in the following circumstances:

- Intercurrent event not compatible with continuation of study
- Participant's decision (data already collected during research may be retained and used unless the subject objects)
- Lost participant
- Investigator's decision

8.6 METHODS OF CARRYING OUT PARTICIPANT EVALUATIONS

For T1 and T2, participants will be received in the early afternoon at GIP Cyceron (Caen) to perform MRI sequences in the presence of an investigating physician.

Further assessments will take place at the PFRS, located in the immediate vicinity of Cyceron.

At T3, participants will be received directly by the team at PFRS Caen to carry out all the assessments.

The spatial memory task, the cognitive tests battery and the questionnaire sessions will be carried out in a specially adapted room at UMR 1077, with a psychologist specialized in neuropsychology.

The polysomnography equipment will be installed and removed by two specialized sleep technicians in a dedicated room at UMR 1077.

The vHIT will be performed at times T1, T2 and T3 at the PFRS by a technician trained to perform this test. The results will be interpreted by a physician specializing in vestibular function and attached to the ICANSLEEP protocol.

The night time during which participants will wear the polysomnography equipment is not included in the above durations. For the night between the 2 half-days of assessment, participants can either go home or stay in a hotel, at their own expense. At each stage of the assessment, participants will be given part of the questionnaires (sleep and quality of life) to complete at home.

A total indemnity of 300 € will be given to each participant in return for the constraints and time invested during their participation in the study. In the event of premature exit from the study, compensation will be paid on a pro rata basis, i.e. €100 per evaluation time completed or not.

9 EVALUATION TOOLS

9.1 Quality and physiology of sleep and circadian rhythms

In addition to subjective measures (self-questionnaires), objective measures (actigraphy, PSG) will be used to collect the participants' sleep quantity and quality indices.

9.1.1 Self-assessment of sleep and circadian typology

Sleep questionnaires (ISI and PSQI) are to be completed at home between the two evaluation sessions, to provide information on subjective sleep parameters. The ISI focuses specifically on insomnia-related symptoms, while the PSQI measures participants' overall subjective sleep quality. The Horne and Ostberg questionnaire [36] provides information on circadian typology.

THE ISI [37] is a 7-item Likert scale assessing symptoms associated with insomnia. A score above 8 indicates the presence of a complaint associated with insomnia.

The PSQI [38] is a questionnaire evaluating 7 components associated with sleep (quality, duration, sleep onset latency, sleep efficiency, use of hypnotics, sleep alterations and impact on daytime functioning). The score ranges from 0 to 21, with a score of 5 or more considered to represent a complaint of poor sleep.

Horne and Ostberg's circadian typology questionnaire [36] includes 19 items to determine circadian typology (very much morning, moderately morning, neutral, moderately evening, very much evening) on the basis of a total score between 16 and 86.

All 3 questionnaires can be completed in 15 minutes.

9.1.2 Sleep/wake cycle test

To quantify periods of activity and rest, and thus the alternation of sleep/wake rhythms, an actigraph (a watch-sized accelerometer worn on the wrist) with a sleep diary will be provided to participants 2 weeks before each assessment time (T1, T2 and T3).

The main parameters derived from the analysis of actigraphy data are the amplitude and acrophase of the activity-rest rhythm.

9.1.3 Examination of nocturnal sleep

At each stage of the study, PSG sleep recording will be carried out at the participants' homes or hotels, using a portable device (Siesta, Compumedics). The equipment will be set up at the PFRS by our research team, and will take around 2 hours to set up. Recordings of brain activity (electroencephalography), eye movements, heart rate, respiratory rate and oxygen saturation will be made simultaneously throughout the night. Sleep scoring (i.e. characterizing the alternation of different sleep stages) will be carried out by a sleep technician. The technician's intervention will also detect any sleep apnea syndromes, and participants will be referred to a specialist if necessary. Participants will be asked not to drive while wearing the polysomnography equipment.

The main parameters derived from PSG analyses are the number of intra-sleep arousals and sleep efficiency (total sleep time/time spent in bed).

9.1.4 Physiological measurements related to circadian rhythms and stress response

Repeated saliva sampling will be carried out at each stage of the study (*Table 3*) to study changes in salivary cortisol levels, one of the markers of circadian rhythms, as well as certain stress-related responses (*Cortisol Awakening Response*).

Table 3 Cortisol measurements according to evaluation times

	T1 and T2	T3
**Before and after MRI**	•	
**CAR**<br>- **Waking up**<br>- **30 min.**<br>- **45 min.**	•	•
**Bedtime**	•	•
**Before and after rest**	•	•
**Total measurements**	8	6

Note. *CAR: Cortisol Awekening* Response

Participants will be provided with Salivette® systems for saliva testing. This device involves inserting a cotton ball into the mouth and soaking it in saliva for 2-3 minutes, then returning it to the storage tube.

Participants will receive a description of the procedure to remind them of the instructions (no drinking, no eating, no smoking, no brushing teeth in the 30 minutes prior to collection) and a table to enter the time of collection. Morning samples will be collected at the participant's home or hotel, and placed in an insulated bag to be stored at 4°C before being transported to the laboratory in the morning. The remaining samples will be collected in our research unit. All salivary samples will be centralized and stored at -80°C at the Centre François Baclesse, in a secure environment with optimum traceability. These samples will be sent to Hélène Castel's team in Rouen (Inserm, U1239, Dir: Dr Youssef Anouar) for salivary cortisol determination. Parameters that may influence salivary cortisol levels will also be collected (age, body mass index, smoking habits, contraceptive methods, socio-educational status).

9.2 Functional connectivity and cortical structures

aMRI and fMRI examinations will be offered on the same day at T1 and T2 of the study. The complete procedure will be explained to participants before they set up. Anatomical volumes will be acquired first, followed by fMRI sequences (activation and resting task). An off-camera debriefing questionnaire (10 min) will then be administered. A video recording will be made of the participants' faces, to check that they have not slept during the resting fMRI session. Similarly, physiological measurements will be taken during the MRI examination to enable adequate pre-processing of the data, as physiological parameters are known to have an impact on the BOLD signal. To this end, a physiological signal recording system (BioPac) will be used to jointly measure respiration and pulse wave or plethysmography (oxygen saturation of the blood) with the same box. Respiration will be measured using a strap placed over the chest. The pulse wave is measured with an oximeter placed on the index finger.

aMRI and fMRI examinations will be carried out on the 3T GE Premier imager in the Cyceron site. The GIP CYCERON holds an authorization for research involving the human person for studies in physiology and physiopathology in humans using nuclear medicine and magnetic

resonance imaging techniques. This authorization to carry out MRI studies was issued by the ARS on September 22, 2020 for a period of 3 years.

The measures selected are presented in Table *4*.

Table 4 MRI measurements

MRI sequences	Test	Usefulness/measures taken	Duration ≃
**Anatomical (aMRI)**	T1 3D	Functional data pre-processing<br>Cortical surface	6 min
	T2 Flair	DTI data pre-processing<br>White matter lesions	6 min
	DTI	White matter microstructure	8 min
**Functional (fMRI)**	Rest task	Rumination<br>Activation control<br>Functional connectivity	12 min
	Activation task (ANT)	Attentional performance<br>Cognitive control<br>Functional connectivity	20 min
			**Total: 52 min**

Note. DTI: Diffusion Tensor Imaging; ANT: Attentional Networks Test

9.2.1 aMRI

Two different types of weighted anatomical images will be acquired during this MRI session: T1 and T2 FLAIR. A diffusion-weighted sequence (DTI) will also be acquired during this anatomical session.

These acquisitions will enable us to study the cerebral anatomy of all participants, using various indices: gray matter density, gray matter volume, cortical thickness, sulcus width, length and depth. The different weightings will play a role in image analysis and processing: registration of functional acquisitions (fMRI) on the anatomical image, segmentation of the three tissue groups (gray matter, white matter, cerebrospinal fluid). These will enable us to obtain images with different contrasts, which will also be useful for better assessing the presence of significant abnormalities. These examinations will help to ensure the absence of lesions, and will also be useful for data processing and analysis. A doctor present at the

session will be responsible for carrying out this verification at the time of the examination, and for authorizing the participant to continue or not to continue the study, depending on the MRIa results. In the event of any anomalies, these will be communicated to the participant and her attending physician, who will take charge of the medical follow-up of the patient excluded from the study.

9.2.2 fMRI

If no abnormalities are detected during the fMRI examination, instructions will be given to the participant before starting the ***resting-state fMRI*** acquisition. The acquisition will take place in a quiet, dark environment. Participants will be asked to keep their eyes open, avoid any specific cognitive task or other effort, without talking or moving their head, and to remain awake throughout the examination. During the examination, participants can let their thoughts flow freely. At the end of this session, participants will be offered an off-camera debriefing, enabling them to accurately report on the nature of their thoughts and bodily sensations during the resting fMRI examination. *Self-related* mental activities are often reported in resting-state studies, and include autobiographical reminiscence, mental imagery, self-talk and future planning. Participants will also be asked to describe whether they were fully awake throughout the examination. Questions specifically associated with rumination phenomena will also be proposed. Participants will be asked to accurately describe these mental activities using a questionnaire and analog scales. Analysis of the resting-state fMRI data will consist in modeling the spontaneous low-frequency fluctuations of the BOLD signal, characteristic of network activity in the baseline state.

Activation fMRI acquisition will be organized in 3 sessions of 5 minutes each, thus reducing the attention maintenance time. The ANT (*Attentional Network Test*) task, described in the following section of this document, will be employed and the functional connectivity between the regions involved in each attentional network, described below (*Table 5*), will be quantified and compared between groups.

9.3 Neuropsychological tests

9.3.1 Overall cognitive efficiency

The MoCA will be used to measure global cognitive efficiency for each participant. It will be realized at T1 only.

9.3.2 Spatial memory task

A computerized spatial memory task, inspired by that of [39] and [32] will be proposed to participants.

This task consists of learning and recalling the position of 4 objects (plastic duck, hat, balloon, hat) in a circular virtual environment (*Figure 4*). The environment represents a meadow delimited by a border (low stone wall) behind which various landmarks are visible (mountain, clouds, sun). An intra-environment marker (traffic cone) is also present. Before carrying out the task, the participants will be asked to familiarize themselves with the task.

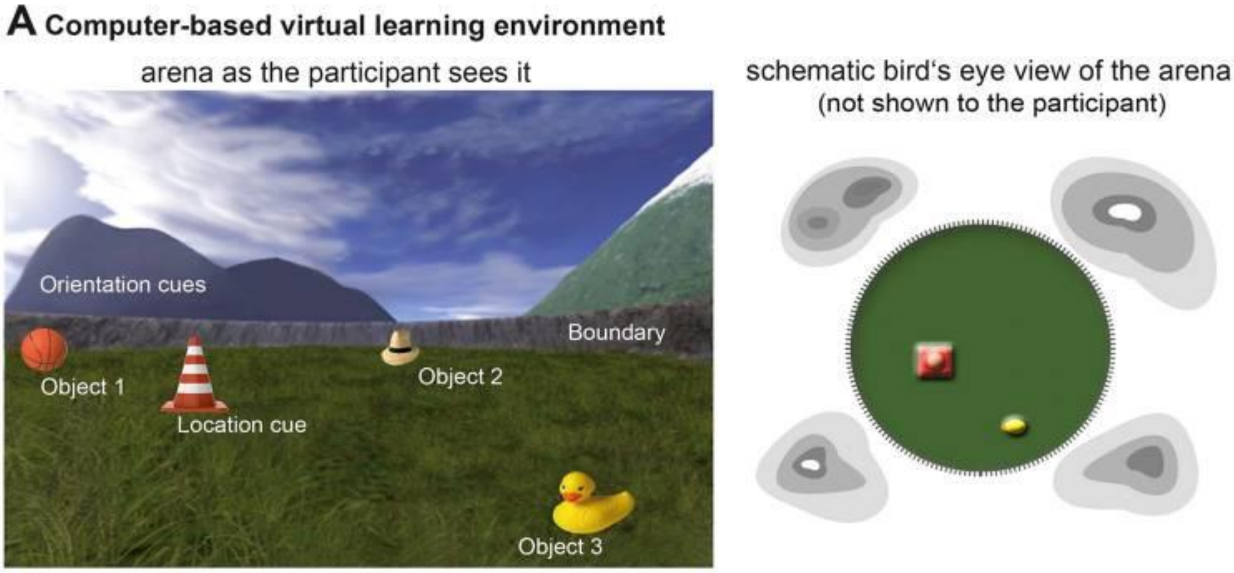

Figure 4. Spatial memory task (Hilliard et al, 2019)

This spatial memory task will be spread over the two days of evaluation, so as to offer delayed recall after a night's sleep. Thus, the proposed task consists of the four phases described below:

Day 1

Phase 1 - Encoding

During this first phase, participants will be asked to explore the location of the 4 objects (one trial per object). Once participants consider that they have retained the object's location, they will be asked to collect the object by pressing on it, making it disappear before moving on to the next object.

Phase 2 - Learning

This second phase consists of 3 series of 4 trials each (one trial per object, in pseudo-random order). For each trial, one of the 4 objects appears alone on the screen against a neutral background for 4 seconds before disappearing. Participants must then recall the position of the memorized object by pressing on the screen, followed by feedback showing the exact location of the object.

Phase 3 - Transfer

For this last phase, proposed directly after the previous one, the aim is to recall the position of the 4 objects in the virtual environment, which will be manipulated by modifying the boundary (low stone wall) or the intra-environment landmark (traffic cone).

- **Situation 1:** The distance between the center of the environment and the boundary (low stone wall) is increased by 20%. The intra-environment marker (traffic cone) remains unchanged.
- **Situation 2:** The intra-environment marker (traffic cone) is moved by 30 virtual meters (vm). The boundary (low stone wall) remains unchanged.

Each object will be presented in both situations, for a total of 8 trials in this phase.

Day 2 (after night's sleep)

Phase 4 - Delayed recall

Participants are asked to recall the positions of the 4 objects they memorized the day before, in a recall phase similar to phase 2.

Phase 5 - Transfer

For this last phase, proposed directly after the previous one, the aim is to recall the position of the 4 objects in the virtual environment, which will be manipulated by modifying the boundary (low stone wall) or the intra-environment landmark (traffic cone).

- **Situation 1:** The distance between the center of the environment and the boundary (low stone wall) is increased by 20%. The intra-environment marker (traffic cone) remains unchanged.
- **Situation 2:** The intra-environment marker (traffic cone) is moved by 30 virtual meters (vm). The boundary (low stone wall) remains unchanged.

Each object will be presented in both situations, for a total of 8 trials in this phase.

Measurements

- Distance (in vm) between recalled position and correct position

9.3.3 Attentional Network Test (ANT)

This attentional task, developed by Fan and colleagues [40,41] will be used as an fMRI activation task at T1 and T2, and as a behavioral measure only at T3. ANT provides measures of the efficiency of 3 distinct attentional networks [42] underlying 1) alerting (achieving and maintaining an alert state), 2) orienting (selecting information on the basis of sensory inputs) and 3) executive control (conflict resolution between multiple responses). These three systems rely on specific neuroanatomical correlates and neurotransmitters (*Table 5*).

Table 5 ANT measurements

**Attentional network**	**Measurements**	**Neuroanatomical correlates**
Alert	TR condition with index - TR condition without index	Right frontal and parietal cortex, reticular formation
Orientation	TR condition with orientation index - TR without orientation index	Superior parietal lobe, thalamus, middle frontal gyrus
Executive control	TR congruent condition - TR non-congruent condition	Anterior cingulate cortex, frontal lobe, especially dorsolateral prefrontal cortex

Note. *ANT: Attentional Network Test; TR: Response Time*

In this computerized task (*Figure 5*), participants will be asked to determine the direction of an arrow (left or right) among other arrows ("*flankers*") in the same direction (congruent situation) or in the opposite direction (incongruent situation). The arrows appear after a variable duration (between 300 and 11800 ms) above or below a fixation point and are preceded or not by a cue according to 3 possible conditions: 1) no cue, 2) central cue 3) spatial cue. The task consists of 6 series of 36 trials + 2 trials not taken into account.

Measurements

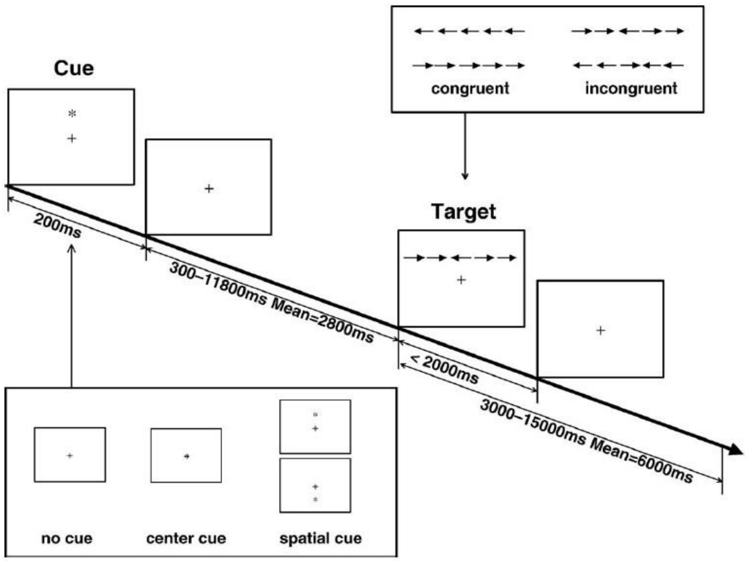

Figure 5. Diagram **of** *the Attentional Network Test*, from Fan et al. (2005)

Response time (RT) is counted for each trial, giving rise to 3 distinct scores presented in Table 5.

9.3.4 Classical neuropsychological tests

The various neuropsychological tests proposed to the participants at T1, T2 and T3 are presented in the following table (*Table 6*).

Table 6. Neuropsychological tests proposed to participants and associated measures

Cognitive function	Test	Measures adopted	Duration ≃
**Episodic memory**	HVLT [43]<br>• Form 1 (T1)<br>• Form 2 (T2)<br>• Form 4 (T3)	Total free recall (/36)	15 min
		Deferred free recall (/12)	
**Working memory**	Space Memory Subtest (MEM-III) [44]	Total direct order score (/16)	5 min
		Total score reverse order (/16)	

**Attention**	Baddeley's double task [45]	Empan task: number of correct sequences; digit span	10 min.
		Motor task: number of boxes ticked in 2 minutes	
		Double task: number of correct sequences; number of ticked boxes	
	d2-R [46]	Number of target characters processed (CCT)	5 min
		Concentration capacity: CCT - errors	
		% errors: (errors / CCT) x 100	
**Executive functions**	N-Back [47]	Number of correct items	5 min
	TMT A and B [45]	Time to complete part A (sec)	5 min
		Time to complete part B - A (sec)	
	Stroop [45]	Completion time Name (sec)	5 min
		Realization time Reading (sec)	
		Realization time Interference - Denomination (sec)	
	Spelling fluency [48]<br>• P (T1)<br>• R (T2)<br>• V (T3)	Number of correct productions	2 min
**Note.** *HVLT: Hopkins Verbal Learning Test; MEM-III: Wechsler Clinical Memory Scale - 3ème edition; TMT: Trail Making Test*			**Total : 1h**

All the neuropsychological tests will be offered in two sessions (30 min each), between which a 20-min questionnaire session and the removal of the PSG (45 min) will take place, at each time of the study and in the same chronology (*Figure 6*).

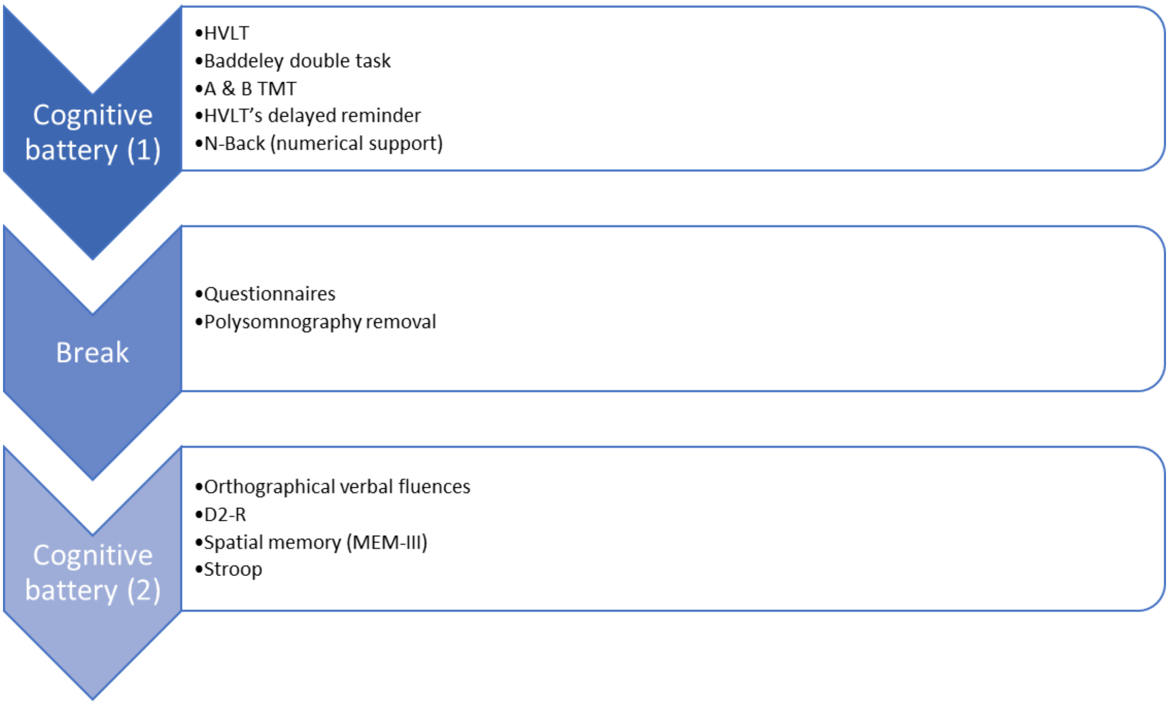

Figure 6. Cognitive battery timeline

Note. *MEM-III: Wechsler Clinical Memory Scale - 3ème edition; HVLT: Hopkins Verbal Learning Test; TMT: Trail Making Test*

9.4 Quality of life

The questionnaires used to measure the various quality of life domains are presented in the following table (*Table 7*). Some will be given to be completed at home, while others will be carried out during two questionnaire sessions lasting around 20 min (*Figure 7*).

Table 7. Questionnaires proposed to participants and associated measures

Domain	Questionnaires	Measurements (range)	Filling time ≃
**General**	FACT-G* [49] *patients only*	Total score (0-108)<br>Physical well-being (0-28)<br>Social/family well-being (0-28)<br>Emotional well-being (0-24)<br>Functional well-being (0-28)	5 min
**Pain**	BPI [50]	Pain severity (0-10); Pain interference (0-10)	10 min.
	FACIT-F* [51] *patients only*	Score (0-52)	5 min

**Fatigue**	MFI-20* [52]		General/physical fatigue (9 - 45)<br>Mental fatigue (6 - 30)<br>Activity reduction (3 - 15)<br>Reduced motivation (2 - 10)	5 min
**Sleepiness**	KSS		Total score (0-9)	1 min (x4)
**Cognitive complaint**	FACT-Cog* [53]		PCI (0-72) ; QoL (0-16) ; Oth (0-16) ; PCA (0-28)	10 min.
**Physical activity**	IPAQ [54]		Continuous score<br>Category score	5 min
**Anxiety - Depression**	STAI-Y [55]	Status	Condition score (20-80)	5 min
		Feature	Trait score (20-80)	5 min
	BDI-II [56]		Total score (0-39)	5 min
**Perceived stress**	PSS [57]		Emotional distress (0-24)<br>Personal effectiveness (0-16)	5 min
**Post-traumatic stress**	PCL-5 [58] *patients only*		Total score (0-80)	5 min
**Post-traumatic growth**	PTGI [59] *patients only*		Overall score (0-105)<br>Relationship with family and friends (0-35)<br>New possibilities (0-25)<br>Personal strengths (0-20)<br>Life appreciation (0-15)<br>Spiritual change (0-10)	5 min
**Coping**	Brief COPE [60–62]	Dispositional modified (before suspicion of cancer) *patients only, only at T1*	Social support research score (8-32)<br>Score Problem solving (4-16)<br>Avoidance score (10-40)<br>Score Positive thinking (6-24)	5 min
		Dispositional		5 min
				**Total :**<br>**PATIENTS: 25 min (home) + 2×20 min**

	**WOMEN WITH CANCER: 15 min (home) + 2 × 20 min**

Note. * : *Questionnaires to be completed at home ; Fact-G : The Functional Assessment of Cancer Therapy - General ; BPI : Brief Pain Inventory ; FACIT-F : Functional Assessment of Chronic Illness Therapy - Fatigue ; MFI-20 : MFI-20 : Multidimensional Fatigue Inventory ; KSS : Karolinska Sleepiness Scale ; Fact-Cog : Functional Assessment of Cancer Therapy - Cognitive Function ; IPAQ : International Physical Activity Questionnaire ; PCI : Perceived Cognitive Impairment; QoL: Impact on Quality of Life; Oth: Comments from Others; PCA: Perceived Cognitive Abilities; STAI-Y: State-Trait Anxiety Inventory; BDI-II: Beck Depression Inventory; PSS: Perceived Stress Scale; PCL-5: Posttraumatic Stress Disorder Checklist for DSM-5; PTGI: Post-Traumatic Growth Inventory; Brief COPE: Brief Coping Orientation to Problems Experienced*

9.5 Evaluation of vestibular function

A recent study found that the prevalence of vestibular disorders such as vertigo and dizziness was 15-20% in adults [63]. In addition, studies suggest that some breast cancer treatments have effects on vestibular function [64 – 67].

The vHIT (Vestibular Head Impulse Test [68]) is a rapid, non-invasive test for objective assessment of vestibular function, and more specifically of each of the six semicircular canals individually. It is generally used to detect possible vestibular dysfunctions as part of the assessment of vertigo and balance disorders.

During the test, a helmet is placed on the participant's head, with micro-cameras recording eye movement. The operator will manually apply rapid head movements in different directions. During the movement, the helmet will record both head and eye movements. From this data, the physician will be able to assess whether vestibular function is intact or affected, and what the condition is, if any.

10 GALVANIC VESTIBULAR STIMULATION (GVS)

10.1 Description of the technique

The GVS principle is based on the use of two electrodes included in the stimulation helmet, a cathode (negative) and an anode (positive) placed on the right and left mastoid bones, behind the ears. The first has an excitatory role, while the second has an inhibitory one.

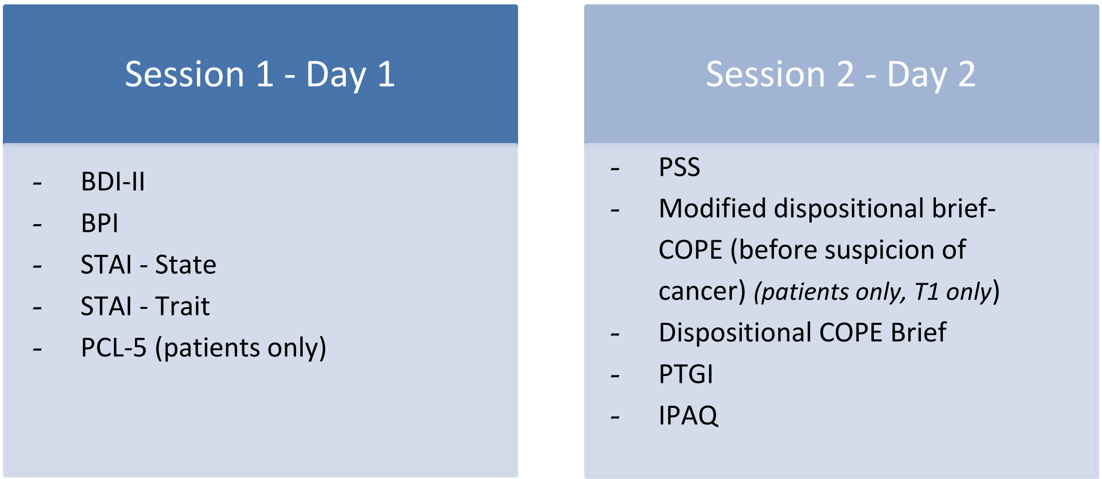

Figure 7. Questionnaires proposed during the 2 evaluation sessions

Note: the KSS is offered at the beginning and end of each assessment day, i.e. a total of 4 times.

A direct electric current is passed through these electrodes to stimulate the irregular fibres of the vestibulocochlear nerve [29]. Sponges that have been soaked in a saline solution will be positioned at the electrodes' location, for better contact with the skin. Electric power intensity will be 1mA [29].

A stimulation headset (**Erreur ! Source du renvoi introuvable.**), already used in other protocols carried out in the COMETE unit, will be made available to participants at time T2 of the study. This headset, marketed by Soterix, is CE-marked and delivers vestibular stimulation defined by the experimenter in a pre-regulated, pre-programmed way. Participants will be briefed on the principle of use, and will be able to familiarize themselves with its operation before taking the equipment home. In addition, participants will already have been familiarized with the video-conferencing platform at the University of Caen during T1.

During the 2 weeks of stimulation, participants will receive an email reminding them of their appointment via the video-conferencing platform. They will then be able to connect at the given time (in the morning, around 9:30 a.m.) and will be in visual contact with one of the team's experimenters, who will check that the headset is correctly positioned and will be able to talk to the participant. The stimulation system is pre-programmed and cannot be modified unless you have a specific code known only to the research team. Once the position of the headset and sponges has been checked, stimulation will be performed for 20 minutes, during which time the participants will be seated and remain in contact with the team. At the end of the 20 minutes, three visual analog scales for pain, fatigue and well-being will be offered.

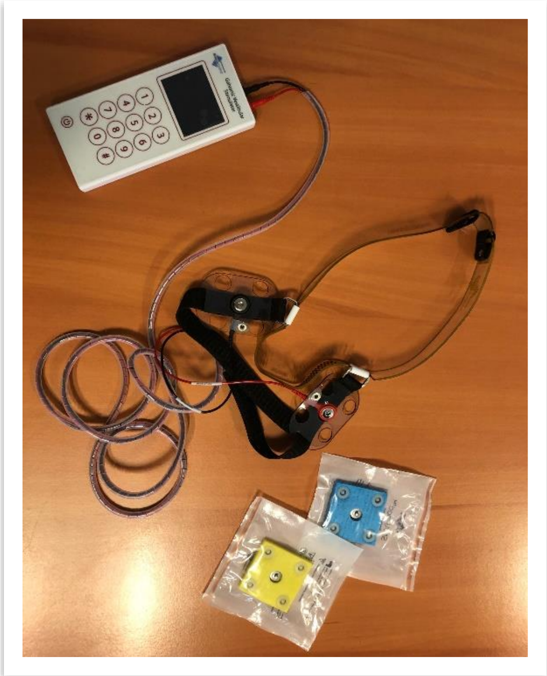

Figure 8 All the equipment required for vestibular stimulation, including the device for controlled, pre-programmed delivery of the stimulation protocol, the headset and the individually delivered sponges.

10.2 Contraindications and expected side effects

GVS induces oculomotor (i.e. nystagmus), postural and perceptual (e.g. head turning) responses. However, the responses observed depend on the type of stimulation, the electrodes, the individual and the context. Some individuals feel mainly perceptions of canalicular origin (i.e. rotation), while others will have more perceptions from otolith afferents (i.e. tilt). In order to limit these side effects, the electrical current threshold will be adapted to each participant, and we will ask participants to perform the stimulation in a lighted room to enable them to have a visual fixation point, which will suppress the visual response. [68]. Finally, the Graybiel scale [69] as well as visual analog scales for fatigue, well-being and pain will be used to quantify patients' feelings and perceived adverse effects.

11 SAFETY CONDUCTED AS CARE VIGILANCE

In accordance with the new research regulation involving the human person, there will be no collection of serious adverse events organized by the sponsor as part of the study.

However, as for all research involving the human person, the sponsor will transmit to the investigators concerned any information likely to affect the safety of persons (Art R1123-52

CSP) and will inform without delay the competent authority and the protection committee of new safety facts and, where appropriate, measures taken (Article R1123-59 of the CSP).

Nevertheless, health professionals are reminded that care vigilance applies, as a result any incident or adverse reaction suspected to be due to a drug or other health product as defined in Article L5311- 1 must be reported by the health professional to the vigilance networks (internet Portal for reporting adverse health events) who, after analysis, report them to the ANSM.

12 STATISTICAL CONSIDERATIONS

12.1 NUMBER OF SUBJECTS REQUIRED

The number of participants in this descriptive and exploratory study is not subject to power calculation. We plan to include 50 breast cancer patients, 25 treated with chemotherapy and 25 not treated with chemotherapy, as well as 25 healthy female volunteers (control group), for a total of 75 participants.

12.2 STATISTICAL ANALYSIS

Axis 1: In order to characterize the sleep of breast cancer patients before and after adjuvant chemotherapy, sleep parameters measured by polysomnography (in particular sleep efficiency and number of awakenings) or by questionnaire will be described in terms of headcount for qualitative variables, and mean and standard deviation (or median and range) for quantitative variables, at T1 and T2 in each of the groups of interest. Similarly, circadian rhythm parameters measured by actigraphy (e.g. mean amplitude over a week) or by salivary cortisol assay will be described at each time and by group. To assess the effect of cancer on sleep and circadian rhythm, parameters at T1 and T2 will be compared between patients and controls using a Chi² test or a Student (or Wilcoxon) test. To assess the effect of chemotherapy on sleep and circadian rhythm, the parameters of chemotherapy-treated patients will be compared between T1 and T2 by a McNemar test or a Student (or Wilcoxon) test for paired series. Alternatively, the effect of chemotherapy on T2 parameters can be tested in linear or logistic models adjusted for T1 parameters. Relationships between sleep parameters and circadian rhythm parameters will be assessed using a correlation matrix (Pearson's or Spearman's). The relationship between sleep parameters and scores on cognitive tests and quality-of-life assessments can be assessed at any time using a linear mixed model (taking into account correlation between repeated measures of the same subject).

Axis 2: In order to assess the effect of galvanic vestibular stimulation on circadian rhythms, the evolution of circadian rhythm parameters measured by actigraphy during the 2-week intervention (in particular the amplitude and phase of the activity-rest rhythm) will be described by a linear mixed model. The model will include a time effect, an intervention effect (GVS or SHAM) and a time x intervention interaction (parameter of interest to assess whether GVS has an effect on the evolution of circadian rhythm compared with SHAM), and will be adjusted for group (patients or controls) and possibly treatment (chemotherapy or not). To meet secondary objectives, sleep parameters, cortisol levels, cognitive abilities and quality of life assessments will be described before (T2) and after intervention (T3) in each group of patients (GVS or SHAM) and controls (GVS only) and compared pre-post GVS by a McNemar test or a Student (or Wilcoxon) test for paired series. Alternatively, the effect of GVS on sleep, cognitive ability

and quality of life at T3 could be tested in linear or logistic models adjusted for parameters at T2.

12.3 NON-COMPLIANCE WITH PROTOCOL

Any deviations from this protocol, as well as any modifications that may occur after the start of the study, will be discussed in the study report.

13 QUALITY ASSURANCE

In order to guarantee the authenticity and credibility of data in accordance with GCP, the sponsor will implement a quality assurance system that includes:

- trial management according to Clinical Research Unit procedures,
- quality control of investigator site data by the monitor, whose role is to check the concordance and consistency of the data in the observation book with the source documents,
- the provision of dedicated staff in the department, if funding allows, to help the investigator with study logistics and data collection in observation notebooks.

14 ETHICAL AND REGULATORY CONSIDERATIONS

The study will be conducted in accordance with the French Public Health Law, specially relating to biomedical research involving the human person of the Public Health Code, articles L1121-1 and following (Law No. 2012-300 of 05/03/2012 as amended by Ordinance No. 2016-800 of 16 June 2016), the Bioethics Law, the law related to the protection of physical persons for the treatments of personal data and related to information technology, database and liberties, the Helsinki declaration and the Good Clinical Practices.

14.1 REGULATORY APPROVALS

A request for authorization will be sent by the Sponsor to the Comité de Protection des Personnes (CPP) before the start of the study. The Competent Authority (ANSM) will be informed, and the summary of the study and the CPP's favorable opinion will be forwarded.

This study falls within the scope of the "Reference Methodology" (MR-001) in application of the provisions of article 54 paragraph 5 of the modified law of January 6, 1978 relating to data processing, files and freedoms. This change was approved by a decision dated January 5, 2006.

The Centre François Baclesse complies with the regulations in force, in particular the rights of persons subject to processing within the meaning of EU Regulation No. 2016/679 on data protection ("RGPD").

Any substantial modification of the protocol, concerning study objectives, design, population, examinations or significant administrative aspects, will require the approval of the coordinating investigator, the sponsor, the favorable opinion of the CPP and the information of the competent authority before implementation.

14.2 PARTICIPANT INFORMATION AND WRITTEN INFORMED CONSENT FORM

Participants will be fully and fairly informed, in comprehensible terms, of the objectives and constraints of the study, the possible risks involved, the necessary monitoring and safety measures, their right to refuse to participate in the study or the possibility of withdrawing at any time.

All this information appears on an information and consent form given to the subject (patient or cancer-free woman). The subject's free, informed and written consent will be obtained by the investigator, or a physician representing him/her, prior to final inclusion in the study. One copy of the information and consent form signed by both parties will be given to the subject, the other copy will be kept by the investigator. For any substantial modification of the protocol, concerning the study's objectives, design, population, examinations or significant administrative aspects, a new consent from the persons taking part in the research will be obtained if necessary.

14.3 STUDY CONDUCT AND INVESTIGATOR RESPONSIBILITIES

The principal investigator of each institution concerned undertakes to conduct the clinical trial in accordance with the protocol approved by the CPP. The investigator must not make any changes to the protocol without the sponsor's authorization, and without the CPP having given a favorable opinion on the proposed modifications.

It is the responsibility of the principal investigator:

- ✓ provide the promoter with his curriculum vitae and those of the co-investigators,
- ✓ identify the team members involved in the trial, and define their responsibilities,
- ✓ to start recruiting patients after authorization from the sponsor,
- ✓ make every effort to include the required number of patients within the established recruitment period.

It is the responsibility of each investigator to:

- ✓ obtain the patient's dated and personally signed informed consent prior to any trial-specific selection procedure,
- ✓ regularly complete case report forms (CRFs) for each patient included in the trial, and give the sponsor's monitor direct access to source documents so that he or she can validate CRF data,
- ✓ accept regular visits from the instructor and, if necessary, auditors appointed by the promoter or inspectors from the supervisory authorities.

All study documentation (protocol, consents, case report forms, investigator file, etc....), as well as original documents (laboratory results, X-rays, consultation reports, reports of clinical examinations performed, etc.) must be made available to the sponsor or persons acting on behalf of the sponsor, and must be kept in a safe place and treated as confidential material.

Data archiving is the responsibility of the investigator, in accordance with current legislation. Documents to be archived include the protocol and appendices, including any amendments, original signed information and consent forms, questionnaires, CRFs and a patient identification list. All these documents will be kept for a minimum of 15 years after the end of the study.

14.4 DATA OWNERSHIP AND CONFIDENTIALITY

The investigator undertakes, for himself and for all persons involved in the trial, to guarantee the confidentiality of all information relating to the project until publication of the trial results. This obligation of confidentiality will not apply to information that the investigator may communicate to participants as part of their participation in the trial, nor to information that has already been published.

The investigator undertakes not to publish, divulge or use in any way whatsoever, directly or indirectly, any scientific or technical information relating to the trial.

The trial may not be the subject of any written or oral comment without the sponsor's agreement; all information communicated or obtained during the trial is the property of the sponsor, who may freely dispose of it.

15 DATA PROCESSING AND STORAGE

15.1 DATA COLLECTION AND PROCESSING

Data will be managed by the Cancéropôle Nord-Ouest's Data Processing Center (CTD). The CTD provides a database management software package dedicated to clinical research: *Ennov Clinical* (version 7.5.10, ENNOV / CLINSIGHT, 33155 Cenon, France).

This software package, based on an Oracleä database architecture, is designed for the global management of clinical and epidemiological studies, and meets the regulatory requirements linked to this type of study. The CTD's *Ennov Clinical* instance is validated in its IT environment. A data validation plan will be drawn up jointly by the Clinical Research Unit and the Data Processing Center, describing in detail the checks to be carried out for each variable.

A study-specific database will be created, tested and validated before data entry begins. All information required by the protocol must be recorded in the paper or electronic case report forms under the responsibility of the principal investigator, with an explanation given for any missing data. Data must be entered in these notebooks as they are obtained, and the sponsor will be responsible for monitoring.

The data will then be checked by the CTD in accordance with the data validation plan.

The database will be frozen after final quality control, then exported in the appropriate format for statistical analysis, using an automated, validated procedure.

Descriptive statistical analyses are planned and will be carried out before the end of the study. These will in no way interfere with the protocol.

15.2 ARCHIVING

The sponsor must ensure that essential documents relating to the conduct of the study are archived in conditions that guarantee their security, for the minimum period stipulated by GCP, i.e. 15 years after the end of the research.

These documents are the protocol and appendices, including any amendments, original signed information forms and consents, questionnaires, CRFs, follow-up documents, statistical analyses and the final study report.

15.3 DATA OWNERSHIP AND PUBLICATION RULES

The results of this study, the property of the promoter (Centre François Baclesse), will be published in the form of scientific articles. Publications concerning or resulting from this research will be communicated to all investigators and submitted for review by the study coordinators.

Authors will include the investigators who included the most patients, the biostatistician who carried out the data analysis (if applicable), the project leader, as well as participants who made a substantial contribution to the development of the study, to the analysis and interpretation of the results and/or to the drafting of the manuscript. No publication will be made without the agreement of the coordinator and the sponsor. The organization having contributed to the financing of the study will be mentioned in the publication.

Acknowledgements to the members of the supervisory committee will be included in the final publication.

The publication rank will be defined according to the investment made in preparing and carrying out the study.

Specific publications may be produced for ancillary studies.

These works will be the property of all the authors and will be made available to them for cross-disciplinary communications and publications.

Publications relating to the results of any ancillary studies will be subject to the prior agreement of the coordinating investigator and the methodologist; they will follow publication of the main study, which must be referenced.

16 FINANCING AND INSURANCE

16.1 STUDY BUDGET

Any additional costs referred to in the Public Health Code are covered by an agreement negotiated between the CFB and the establishment's representative, taking into account the financial resources available to the CFB for its public promotion activities.

However, the CFB is responsible for organizing the study and supplying the following materials (protocol, observation notebook, investigator file) needed to conduct the study.

If materials or treatments are supplied by other partners, the conditions must be specified in the study agreement.

16.2 INSURANCE

The Sponsor has taken out insurance covering its own civil liability and that of any physician involved in the study for the entire duration of the study. It will also provide full compensation for any consequences of the research for the person taking part and his or her beneficiaries, unless it can prove that the damage is not attributable to its own fault or to that of any other party involved, without being able to invoke the act of a third party or the voluntary withdrawal of the person who had initially agreed to take part in the research (cf. article L 1121-10).

17 BIBLIOGRAPHICAL REFERENCES

1. Roth, T. Insomnia: Definition, prevalence, etiology, and consequences. *Journal of Clinical Sleep Medicine* **3**, S7-S10 (2007).
2. Fiorentino, L. & Ancoli-Israel, S. Insomnia and its treatment in women with breast cancer. *Sleep Medicine Reviews* **10**, 419-429 (2006).
3. Fleming, L. *et al.* Insomnia in breast cancer: a prospective observational study. *Sleep* **42**, (2019).
4. Perrier, J., Duivon, M., Rauchs, G. & Giffard, B. Sleep in non-cerebral cancers: review of the literature, potential mechanisms and prospects for better understanding associated cognitive disorders. *Médecine du Sommeil* **18**, 90-103 (2021).
5. Hsiao, F.-H. *et al.* A longitudinal study of diurnal cortisol patterns and associated factors in breast cancer patients from the transition stage of the end of active cancer treatment to post-treatment survivorship. *Breast (Edinburgh, Scotland)* **36**, 96-101 (2017).
6. Carpenter, J. S., Gilchrist, J. M., Chen, K., Gautam, S. & Freedman, R. R. Hot flashes, core body temperature, and metabolic parameters in breast cancer survivors. *Menopause (New York, N.Y.)* **11**, 375-381 (2004).
7. Martin, T. *et al.* Rest activity rhythms characteristics of breast cancer women following endocrine therapy. *Sleep* zsab248 (2021) doi:10.1093/sleep/zsab248.
8. Ancoli-Israel, S. *et al.* Sleep, fatigue, depression, and circadian activity rhythms in women with breast cancer before and after treatment: a 1-year longitudinal study. *Supportive Care in Cancer: Official Journal of the Multinational Association of Supportive Care in Cancer* **22**, 2535-2545 (2014).
9. Payne, J., Piper, B., Rabinowitz, I. & Zimmerman, B. Biomarkers, fatigue, sleep, and depressive symptoms in women with breast cancer: a pilot study. *Oncology Nursing Forum* **33**, 775-783 (2006).
10. Liu, L. *et al.* Decreased Health-Related Quality of Life in Women With Breast Cancer Is Associated With Poor Sleep. *Behavioral Sleep Medicine* **11**, 189-206 (2013).
11. Liu, L. *et al.* Fatigue and sleep quality are associated with changes in inflammatory markers in breast cancer patients undergoing chemotherapy. *Brain Behavior and Immunity* **26**, 706-713 (2012).
12. Liu, L. *et al.* The longitudinal relationship between fatigue and sleep in breast cancer patients undergoing chemotherapy. *Sleep* **35**, 237-245 (2012).
13. Li, W. *et al.* Disruption of sleep, sleep-wake activity rhythm, and nocturnal melatonin production in breast cancer patients undergoing adjuvant chemotherapy: prospective cohort study. *Sleep Medicine* **55**, 14-21 (2019).
14. Beck, S. L. *et al.* Sleep quality after initial chemotherapy for breast cancer. *Supportive Care in Cancer: Official Journal of the Multinational Association of Supportive Care in Cancer* **18**, 679-689 (2010).
15. Kuo, H.-H., Chiu, M.-J., Liao, W.-C. & Hwang, S.-L. Quality of sleep and related factors during chemotherapy in patients with stage I/II breast cancer. *Journal of the Formosan Medical Association = Taiwan Yi Zhi* **105**, 64-69 (2006).
16. Madsen, M. T., Huang, C. & Gögenur, I. Actigraphy for measurements of sleep in relation to oncological treatment of patients with cancer: a systematic review. *Sleep Medicine Reviews* **20**, 73-83 (2015).

17. Parker, K. P. *et al.* Sleep/Wake patterns of individuals with advanced cancer measured by ambulatory polysomnography. *Journal of Clinical Oncology: Official Journal of the American Society of Clinical Oncology* **26**, 2464-2472 (2008).

18. Williams, R. L., Karacan, I. & Hursch, C. J. *Electroencephalography (EEG) of human sleep: clinical applications*. (John Wiley & Sons, 1974).

19. Roscoe, J. A. *et al.* Few changes observed in polysomnographic-assessed sleep before and after completion of chemotherapy. *Journal of Psychosomatic Research* **71**, 423-428 (2011).

20. Diekelmann, S. & Born, J. The memory function of sleep. *Nature Reviews. Neuroscience* **11**, 114-126 (2010).

21. Wilckens, K. A., Woo, S. G., Kirk, A. R., Erickson, K. I. & Wheeler, M. E. The role of sleep continuity and total sleep time in executive function across the adult lifespan. *Psychology and aging* **29**, 658-665 (2014).

22. van Dalfsen, J. H. & Markus, C. R. The influence of sleep on human hypothalamic-pituitary-adrenal (HPA) axis reactivity: A systematic review. *Sleep Medicine Reviews* **39**, 187-194 (2018).

23. Kreutz, C., Schmidt, M. E. & Steindorf, K. Effects of physical and mind-body exercise on sleep problems during and after breast cancer treatment: a systematic review and meta-analysis. *Breast Cancer Research and Treatment* **176**, 1-15 (2019).

24. Garland, S. N. *et al.* Sleeping well with cancer: a systematic review of cognitive behavioral therapy for insomnia in cancer patients. *Neuropsychiatric Disease and Treatment* **10**, 1113-1124 (2014).

25. Besnard, S. *et al.* The balance of sleep: Role of the vestibular sensory system. *Sleep Medicine Reviews* **42**, 220-228 (2018).

26. Martin, T. *et al.* Vestibular loss disrupts daily rhythm in rats. *Journal of Applied Physiology (Bethesda, Md.: 1985)* **118**, 310-318 (2015).

27. Martin, T. *et al.* Exploration of Circadian Rhythms in Patients with Bilateral Vestibular Loss. *PLOS ONE* **11**, e0155067 (2016).

28. Pasquier, F. *et al.* Effect of vestibular stimulation using a rotatory chair in human rest/activity rhythm. *Chronobiology International* **37**, 1244-1251 (2020).

29. Utz, K. S., Dimova, V., Oppenländer, K. & Kerkhoff, G. Electrified minds: transcranial direct current stimulation (tDCS) and galvanic vestibular stimulation (GVS) as methods of non-invasive brain stimulation in neuropsychology-a review of current data and future implications. *Neuropsychologia* **48**, 2789-2810 (2010).

30. Wilkinson, D., Nicholls, S., Pattenden, C., Kilduff, P. & Milberg, W. Galvanic vestibular stimulation speeds visual memory recall. *Experimental Brain Research* **189**, 243-248 (2008).

31. Wilkinson, D., Ferguson, H. J. & Worley, A. Galvanic vestibular stimulation modulates the electrophysiological response during face processing. *Visual Neuroscience* **29**, 255-262 (2012).

32. Hilliard, D. *et al.* Noisy galvanic vestibular stimulation modulates spatial memory in young healthy adults. *Scientific Reports* **9**, 1-11 (2019).

33. Pasquier, F., Denise, P., Gauthier, A., Bessot, N. & Quarck, G. Impact of Galvanic Vestibular Stimulation on Anxiety Level in Young Adults. *Frontiers in Systems Neuroscience* **13**, 14 (2019).

34. Voros, J. L. *et al.* Galvanic Vestibular Stimulation Produces Cross-Modal Improvements in Visual Thresholds. *Front Neurosci* **15**, 640984 (2021).

35. Putman, E. J., Galvan-Garza, R. C. & Clark, T. K. The Effect of Noisy Galvanic Vestibular Stimulation on Learning of Functional Mobility and Manual Control Nulling Sensorimotor Tasks. *Front Hum Neurosci* **15**, 756674 (2021).

36. Horne, J. A. & Ostberg, O. A self-assessment questionnaire to determine morningness-eveningness in human circadian rhythms. *International Journal of Chronobiology* **4**, 97-110 (1976).

37 Morin, C. M. *Insomnia: Psychological assessment and management.* (Guilford press, 1993).

38. Buysse, D. J. *et al.* The Pittsburgh Sleep Quality Index: a new instrument for psychiatric practice and research. *Psychiatry res* **28**, 193-213 (1989).

39. Doeller, C. F., King, J. A. & Burgess, N. Parallel striatal and hippocampal systems for landmarks and boundaries in spatial memory. *Proceedings of the National Academy of Sciences* **105**, 5915-5920 (2007).

40. Fan, J., McCandliss, B. D., Sommer, T., Raz, A. & Posner, M. I. Testing the Efficiency and Independence of Attentional Networks. **14**, 340-347 (2002).

41. Fan, J., McCandliss, B. D., Fossella, J., Flombaum, J. I. & Posner, M. I. The activation of attentional networks. *NeuroImage* **26**, 471-479 (2005).

42. Posner, M. I. & Petersen, S. E. The Attention System of the Human Brain. *Annals of Neuroscience* **13**, 25-42 (1990).

43. Rieu, D., Bachoud-Lévi, A.-C., Laurent, A., Jurion, E. & Dalla Barba, G. French adaptation of the "Hopkins verbal learning test". *Revue Neurologique* **162**, 721-728 (2006).

44. Wechsler III, D. MEM-III. *Wechsler Clinical Memory Scale: manual. Éd. du Centre de psychologie appliquée* (2001).

45 Godefroy, O. & GREFEX. *Executive functions and neurological and psychiatric pathologies*. *Marseille, Solal* (2008).

46. Brickenkamp, R., Schmidt-Atzert, L. & Liepmann, D. *D2-R: concentrated attention test* (Éditions Hogrefe France, 2015).

47. Gevins, A. S. & Cutillo, B. C. Neuroelectric evidence for distributed processing in human working memory. *Electroencephalography and Clinical Neurophysiology* **87**, 128-143 (1993).

48. Cardebat, D., Doyon, B., Puel, M., Goulet, P. & Joanette, Y. Formal and semantic lexical evocation in normal subjects. Performance and production dynamics as a function of sex, age and level of study. *Acta Neurologica Belgica* **90**, 207-217 (1990).

49. Cella, D. F. *et al.* The Functional Assessment of Cancer Therapy scale: development and validation of the general measure. *Journal of Clinical Oncology: Official Journal of the American Society of Clinical Oncology* **11**, 570-579 (1993).

50. Cleeland, C. The Brief Pain Inventory. *Pain Research Group* 143-147 (1991).

51. Webster, K., Cella, D. & Yost, K. The Functional Assessment of Chronic Illness Therapy (FACIT) Measurement System: properties, applications, and interpretation. *Health and Quality of Life Outcomes* **1**, 79 (2003).

52. Gentile, S., Delarozière, J. C., Favre, F., Sambuc, R. & San Marco, J. L. Validation of the French 'multidimensional fatigue inventory' (MFI 20). *European Journal of Cancer Care* **12**, 58-64 (2003).

53. Joly, F. *et al.* French version of the Functional Assessment of Cancer Therapy-Cognitive Function (FACT-Cog) version 3. *Supportive Care in Cancer* **20**, 3297-3305 (2012).

54. Craig, C. L. *et al.* International physical activity questionnaire: 12-country reliability and validity. *Medicine & science in sports & exercise* **35**, 1381-1395 (2003).

55. Spielberger, C. D. Manual for the State-Trait Anxiety Inventory STAI (form Y)(' self-evaluation questionnaire'). (1983).

56. Beck, A. T., Ward, C. H., Mendelson, M., Mock, J. & Erbaugh, J. An Inventory for Measuring Depression, Archives of General Psychiatry, 4. 561-571 (1961).

57. Cohen, S., Kamarck, T. & Mermelstein, R. Perceived stress scale. *Measuring stress: A guide for health and social scientists* **10**, 1-2 (1994).

58. Weathers, F. W. *et al.* The PTSD Checklist for DSM-5 (PCL-5). *Scale available from the National Center for PTSD at www. ptsd. va. gov* (2013).

59. Tedeschi, R. G. & Calhoun, L. G. The Posttraumatic Growth Inventory: Measuring the positive legacy of trauma. *Journal of traumatic stress* **9**, 455-471 (1996).

60. Carver, C. S. You want to measure coping but your protocol's too long: Consider the brief COPE. *International Journal of Behavioral Medicine* vol. 4 92-100 (1997).

61. Muller, L. & Spitz, E. Évaluation multidimensionnelle du coping: Validation du Brief COPE sur une population française. *Encephale* **29**, 507-518 (2003).

62. Baumstarck, K. *et al.* Assessment of coping: A new French four-factor structure of the brief COPE inventory. *Health and Quality of Life Outcomes* **15**, 1-9 (2017).

63. Neuhauser HK. [Epidemiology of dizziness and vertigo]. Der Nervenarzt. 2009 Aug;80(8):887-894.

64. Medina HN, Liu Q, Cao C, Yang L. Balance and vestibular function and survival in US cancer survivors. Cancer. 2021 Nov 1;127(21):4022-4029.

65. Monfort SM, Pan X, Patrick R, Singaravelu J, Loprinzi CL, Lustberg MB, Chaudhari AMW. Natural history of postural instability in breast cancer patients treated with taxane-based chemotherapy: A pilot study. Gait Posture. 2016 Jul;48:237-242.

66. Wampler MA, Topp KS, Miaskowski C, Byl NN, Rugo HS, Hamel K. Quantitative and clinical description of postural instability in women with breast cancer treated with taxane chemotherapy. Arch Phys Med Rehabil. 2007 Aug;88(8):1002-8.

67. Winters-Stone KM, Torgrimson B, Horak F, Eisner A, Nail L, Leo MC, Chui S, Luoh SW. Identifying factors associated with falls in postmenopausal breast cancer survivors: a multi-disciplinary approach. Arch Phys Med Rehabil. 2011 Apr;92(4):646-52.

68. Curthoys, I. S. & MacDougall, H. G. What galvanic vestibular stimulation actually activates. *Frontiers in neurology* **3**, 117 (2012).

69. Graybiel, A., Wood, C. D. & Miller II, E. F. *Diagnostic criteria for grading the severity of acute motion sickness*. vol. 1030 (Naval Aerospace Medical Institute, Naval Aerospace Medical Center, 1968).
