# Supplementary material for: Exploration of effects of galvanic vestibular stimulation on circadian rhythms and its associations with sleep and spatial memory in patients with breast cancer: The ICANSLEEP-2 protocol
Source: PLoS One. 2024 Jul 31;19(7):e0306462. doi: 10.1371/journal.pone.0306462 (PMC11290633; doi:10.1371/journal.pone.0306462)

PROTOCOLE ICANSLEEP

IMPACT DES TROUBLES DU SOMMEIL SUR LA COGNITION ET LA QUALITÉ DE VIE DANS LE CANCER DU SEIN SUITE À LA CHIMIOTHÉRAPIE

ICANSLEEP

N° ID-RCB : 2022-A00437-36

Version n°2.1 du 05/09/2023

Cet essai est soutenu par la Région NORMANDIE, dans le cadre d'un « RIN [Recherche et Innovation » Recherche 2020 « Chaire d'Excellence » et par un financement doctoral de l'école doctorale N°556 - HSRT (HOMME, SOCIÉTÉS, RISQUES, TERRITOIRE).

CLASSIFICATION DE L'ESSAI : RIPH catégorie 2

**PROMOTEUR**	**Centre François Baclesse**<br>3 avenue du Général Harris<br>14076 CAEN cedex 5

**INVESTIGATEUR COORDONNATEUR**	**Dr Carine SEGURA-DJEZZAR**<br><br>Centre François Baclesse<br>3 avenue du Général Harris<br>14076 CAEN cedex 5

**AUTORISATIONS REGLEMENTAIRES**	CPP IDF IV	Date d'avis : 19/04/2022

PERSONNES IMPLIQUÉES DANS LA PRÉPARATION ET LA CONDUITE DE LA RECHERCHE

<table>
<tr><th colspan="2">CENTRES PARTICIPANTS</th></tr>
<tr><th>INVESTIGATEURS</th><th>LIEU DE LA RECHERCHE</th></tr>
<tr><td>INVESTIGATEUR COORDONATEUR
Dr Carine SEGURA-DJEZZAR

CO-INVESTIGATEURS – RECRUTEMENT DES PATIENTES
Dr Christelle LEVY
Dr George EMILE
Dr Djelila ALLOUACHE
Dr Adeline MOREL</td><td>Centre François Baclesse – CAEN</td></tr>
<tr><td>CO-INVESTIGATEURS – RECRUTEMENT DES TEMOINS
Pr Fausto VIADER
Pr Vincent DE LA SAYETTE
Pr Olivier MARTINAUD
Pr Pierre Denise</td><td>INSERM-EPHE-UNICAEN U1077 – CAEN</td></tr>
<tr><th colspan="2">PERSONNES ASSOCIÉES AU PROJET</th></tr>
<tr><td>RESPONSABLE SCIENTIFIQUE
Pr Bénédicte GIFFARD</td><td>UMR_S 1077 Inserm-EPHE-Normandie Université</td></tr>
</table>

**ÉQUIPE DE RECHERCHE U1077**<br>⇨ **PFRS**<br>Joy PERRIER (Post-doctorante)<br>Clara Elia (Doctorante)<br>Francis EUSTACHE (DE)<br>Laura DE GIROLAMO (IE)<br>Patrice CLOCHON (IR1)<br>Franck DOIDY (AI)<br><br>⇨ **Cyceron**<br>Mikaël NAVEAU (IE)<br>Nicolas DELCROIX (IR)	Pôle des Formations et de Recherche en Santé (PFRS)<br>2 rue des Rochambelles CS 14032<br>F-14032-Caen cedex<br><br>**GIP Cyceron**- Bd Becquerel – BP 5229<br>14074 CAEN cedex 5
**ÉQUIPES DE RECHERCHE COLLABORATRICES**<br>Gaëlle QUARCK (PU) – U1075, Caen<br>Olivier ETARD (PH) – U1075, Caen<br><br>Tristan Martin (MCU) – EA 4334, Le Mans<br><br>Hélène Castel (DR) - U1239, Rouen	**UMR-S 1075 – UNICAEN-INSERM**<br>Mobilités : Vieillissement, Pathologie, Santé – COMETE<br><br>**EA 4334, Université Le Mans**<br>Mouvement, Interactions, Performances – MIP<br><br>**U1239, INSERM, Université de Rouen**<br>Différenciation et Communication Neuronale et Neuroendocrine – DC2N
**RECHERCHE CLINIQUE**<br><br>**PROMOTION DE LA RECHERCHE**<br>Bénédicte CLARISSE**, Responsable promotion**<br>Jean-Michel GRELLARD, **Chef de Projet**<br>Justine LEQUESNE**, Méthodologiste**	Centre François Baclesse – CAEN<br><br>**DRCI, Service de recherche Clinique**

TABLE DES MATIÈRES

1 SYNOPSIS 7

2 LISTE DES ABRÉVIATIONS 11

3 SCHÉMA DE L'ÉTUDE 12

4 RÉCAPITULATIF DES EXAMENS À RÉALISER 13

5 JUSTIFICATION SCIENTIFIQUE DE L'ÉTUDE 15

5.1 ÉTAT ACTUEL DES CONNAISSANCES 15

5.2 PRISE EN CHARGE DÉJÀ EXISTANTES ET LEURS LIMITES 17

5.3 DESCRIPTION DE LA NOUVELLE PRISE EN CHARGE PROPOSÉE 17

6 OBJECTIFS DE L'ÉTUDE 18

6.1 OBJECTIF PRINCIPAL 18

6.2 OBJECTIFS SECONDAIRES 18

7 HYPOTHÈSE DE RECHERCHE ET RÉSULTATS ATTENDUS 19

8 CRITÈRES DE JUGEMENT 20

8.1 CRITÈRE PRINCIPAL 20

8.2 CRITÈRES SECONDAIRES 21

9 PLAN DE L'ÉTUDE 22

9.1 MÉTHODOLOGIE 22

9.2 DURÉE DE L'ÉTUDE 22

9.3 SÉLECTION DES PARTICIPANTES 22

9.3.1 Critères d'inclusion des patientes 22

9.3.2 Critères de non inclusion des patientes 23

9.3.3 Critères de sélection des femmes indemnes de cancer 23

9.4 DÉROULEMENT DE L'ÉTUDE 24

9.4.1 Procédure d'inclusion 24

9.4.2 Évaluations de l'étude 24

9.4.3 Bilan d'inclusion 25

9.4.4 Bilans T1 et T2 26

9.4.5 Bilan T3 27

9.5 ARRÊT PRÉMATURÉ DE L'ÉTUDE 28

9.6 MODALITES DE REALISATION DES ÉVALUATIONS DES PARTICIPANTES 28

10 OUTILS D'EVALUTATION 29

10.1 QUALITE ET PHYSIOLOGIE DU SOMMEIL ET DES RYTHMES CIRCADIENS 29

10.1.1 Auto-évaluations du sommeil et de la typologie circadienne 29

10.1.2 Examen du cycle veille/sommeil 29

10.1.3 Examen du sommeil nocturne 29

10.1.4 Mesures physiologiques liées aux rythmes circadiens et à la réponse au stress ... 30
10.2 CONNECTIVITE FONCTIONNELLE ET STRUCTURES CORTICALES ... 31
10.2.1 IRMa ... 32
10.2.2 IRMf ... 32
10.3 TESTS NEUROPSYCHOLOGIQUES ... 33
10.3.1 Efficience cognitive globale ... 33
10.3.2 Tâche de mémoire spatiale ... 33
10.3.3 Attentional Network Test (ANT) ... 35
10.3.4 Tests neuropsychologiques classiques ... 36
10.4 QUALITE DE VIE ... 37
11 STIMULATION VESTIBULAIRE GALVANIQUE (SVG) ... 40
11.1 DESCRIPTION DE LA TECHNIQUE ... 40
11.2 CONTRE-INDICATIONS ET EFFETS INDESIRABLES ATTENDUS ... 41
12 VIGILANCE SANITAIRE RÈGLEMENTAIRE ... 41
13 CONSIDÉRATIONS STATISTIQUES ... 42
13.1 NOMBRE DE SUJETS NÉCÉSSAIRES ... 42
13.2 ANALYSE STATISTIQUE ... 42
13.3 NON RESPECT DU PROTOCOLE ... 43
14 ASSURANCE QUALITÉ ... 43
15 CONSIDERATIONS ÉTHIQUES ET RÈGLEMENTAIRES ... 43
15.1 AUTORISATIONS RÈGLEMENTAIRES ... 43
15.2 INFORMATION DE LA PARTICIPANTE ET FORMULAIRE DE CONSENTEMENT ECLAIRÉ ECRIT ... 44
15.3 CONDUITE DE L'ÉTUDE ET RESPONSABILITES DES INVESTIGATEURS ... 44
15.4 PROPRIETÉS DES DONNÉES ET CONFIDENTIALITÉ ... 45
16 TRAITEMENT ET CONSERVATION DES DONNÉES ... 45
16.1 RECUEIL ET TRAITEMENT DES DONNÉES ... 45
16.2 ARCHIVAGE ... 46
16.3 PROPRIÉTES DES DONNÉES ET RÈGLES DE PUBLICATION ... 46
17 FINANCEMENT ET ASSURANCE ... 47
17.1 BUDGET DE L'ÉTUDE ... 47
17.2 ASSURANCE ... 47
18 RÉFÉRENCES BIBLIOGRAPHIQUES ... 47
19 ANNEXES ... 52

1 SYNOPSIS

**TITRE**	**Impact des troubles du sommeil sur la cognition et la qualité de vie dans le cancer du sein**
**ACRONYME**	**ICANSLEEP**
**Coordonnateur**	Dr Carine SEGURA-DJEZZAR
**Indication**	Patientes présentant un cancer du sein localisé opéré devant débuter ou non un traitement par chimiothérapie adjuvante<br>Femmes sans antécédent de cancer (groupe témoin)
**Méthodologie de l'essai**	Essai bicentrique, longitudinal (évaluations avant et après chimiothérapie, comparaison aux groupes témoins ; et comparaison avant et après stimulation vestibulaire)
**Objectifs**	**Objectifs principaux**<br>**Axe 1 :** Caractériser le sommeil de patientes suivies pour un cancer du sein avant et après chimiothérapie adjuvante par rapport à des patientes non traitées par chimiothérapie et à des femmes sans antécédents de cancer.<br>**Axe 2 :** Explorer l'effet régulateur de la stimulation vestibulaire galvanique (SVG) sur les rythmes circadiens chez les patientes suivies pour un cancer du sein localisé par rapport à une stimulation SHAM (non effective).<br>**Objectifs secondaires**<br>**Axe 1 :**<br>**1)** **a)** Évaluer les effets du cancer et de la chimiothérapie sur le sommeil et les rythmes circadiens<br>**b)** Déterminer si la présence de troubles du sommeil est associée à une altération des rythmes circadiens<br>**2)** Évaluer les effets du cancer et de la chimiothérapie sur les capacités cognitives et leurs corrélats neuro-fonctionnels<br>**3)** Évaluer les effets du cancer et de la chimiothérapie sur la structure corticale<br>**4)** Évaluer les effets du cancer et de la chimiothérapie sur la connectivité fonctionnelle au repos<br>**5)** Évaluer le lien existant entre les troubles du sommeil avec :<br>**a)** Les capacités cognitives et leurs corrélats neuro-anatomiques<br>**b)** Les systèmes physiologiques du stress<br>**c)** La qualité de vie<br>**Axe 2 :**<br>**1)** Évaluer les effets de la SVG sur :<br>**a)** Les rythmes circadiens<br>**b)** La mémoire spatiale<br>**c)** Les systèmes physiologiques du stress<br>**2)** Évaluer les effets de la resynchronisation des rythmes sur la qualité de sommeil<br>**3)** Déterminer si l'amélioration du sommeil est accompagnée de meilleures performances cognitives ainsi que d'une meilleure qualité de vie

**Critères de jugement**	**Critère principal**<br>**Axe 1 :** efficacité de sommeil et nombre d'éveils (polysomnographie) et **Axe 2 :** amplitude et acrophase du rythme activité-repos (actimétrie)<br>**Critères secondaires**<br>**Axe 1 :**<br>**1) a) b)** Actimétrie (amplitude et acrophase du rythme) ; Agenda du sommeil (scores), Cortisol diurne (taux de cortisol) ; Polysomnographie (efficacité du sommeil et nombres d'éveil) ; Questionnaires ISI, PSQI (scores)<br>**2)** Tests neuropsychologiques (scores) ; Tâche de mémoire spatiale (scores) ; IRMf activation – tâche ANT (signal BOLD relatif à la tâche – connectivité effective)<br>**3)** IRMa (structures corticales – substances blanche et grise)<br>**4)** IRMf au repos (fluctuations dynamiques du signal BOLD - connectivité fonctionnelle, corrigées des facteurs de confusions cardiorespiratoires) **;** Questionnaire de débriefing post-IRM (scores)<br>**5) a)** Polysomnographie (efficacité du sommeil et nombre d'éveils) ; Questionnaires ISI, PSQI (scores) ; HVLT (scores) ; Tâche de mémoire spatiale (scores) ; IRMa : (structures corticales – substance blanche) ; Questionnaires STAI, BDI-II (scores)<br>**5) b)** Polysomnographie (efficacité du sommeil et nombres d'éveil) ; Questionnaires ISI, PSQI (scores) ; CAR et cortisol pré- post- IRM (taux de cortisol)<br>**5) c)** Polysomnographie (efficacité du sommeil et nombre d'éveils) ; Questionnaires ISI, PSQI (scores) ; Questionnaires qualité de vie (scores)<br>**Axe 2 :**<br>**1) a)** Actimétrie (amplitude et acrophase du rythme) ; Agenda du sommeil (scores) ; Cortisol diurne (taux de cortisol)<br>**1) b)** Tâche de mémoire spatiale (scores)<br>**1) c)** CAR et cortisol pré- post- IRM (taux de cortisol)<br>**2)** Actimétrie (amplitude et acrophase du rythme), Agenda du sommeil : (scores) ; Polysomnographie (efficacité du sommeil et nombres d'éveil) ; Questionnaires ISI, PSQI (scores)<br>**3)** Polysomnographie (efficacité du sommeil et nombres d'éveil) ; Questionnaires ISI, PSQI (scores) ; Tests neuropsychologiques (scores) ; Questionnaires – qualité de vie (scores)
**Critères d'inclusion (patientes)**	- Patiente âgée de 45 à 65 ans<br>- Patiente atteinte d'un cancer du sein localisé opéré devant ou non débuter un traitement par chimiothérapie adjuvante<br>- Patiente de niveau d'études 3 « fin d'études primaires » (échelle de Barbizet)<br>- Patiente maîtrisant la langue française<br>- Patiente disposant d'un accès à internet depuis un ordinateur au domicile<br>- Patiente ayant signé le consentement pour participer à l'étude<br>- Patiente présentant une plainte de sommeil (ISI > 7)<br>- Patiente ayant accès à internet depuis un ordinateur au domicile

**Critères de non inclusion (patientes)**	- Patiente présentant un cancer métastasique<br>- Patiente présentant un cancer primitif différent du cancer du sein<br>- Patiente ayant un antécédent d'atteinte neurologique<br>- Patiente présentant une apnée du sommeil traitée<br>- Patiente faisant usage de drogue ou ayant une consommation abusive d'alcool (≥3 verres/jour en moyenne et/ou >10 verres /semaine)<br>- Patiente ayant un traitement non stabilisé depuis au moins 3 mois (hypnotiques, antidépresseurs, anxiolytiques)<br>- Patiente présentant un trouble de la personnalité et/ou une pathologie psychiatrique évolutive<br>- Patiente présentant une contre-indication à la réalisation d'une IRM (claustrophobie, objets métalliques dans le corps)<br>- Patiente ayant un problème de vue non corrigé<br>- Patiente présentant des altérations cognitives préexistantes au diagnostic du cancer
**Critères de sélection des volontaires sains**	- Femme issue de la population générale appariée en âge et en niveau d'études (échelle de Barbizet)<br>- Femme de niveau d'études 3 « fin d'études primaires » minimum (échelle de Barbizet)<br>- Femme maîtrisant la langue française<br>- Femme disposant d'un accès à internet depuis un ordinateur au domicile<br>- Femme ayant signé le consentement pour participer à l'étude<br>- Femme ne présentant pas d'antécédent de cancer<br>- Femme présentant une plainte de sommeil (ISI > 7)<br>- Femme ayant accès à internet depuis un ordinateur au domicile<br>- Femme ne travaillant pas en horaires postés<br>- Femme ne présentant pas d'antécédent d'atteinte neurologique<br>- Femme ne présentant pas d'apnée du sommeil traitée<br>- Femme ne faisant pas usage de drogue ou n'ayant pas une consommation abusive d'alcool (≥3 verres/jour en moyenne et/ou >10 verres /semaine)<br>- Femme n'ayant pas de traitement non stabilisé depuis au moins 3 mois (hypnotiques, antidépresseurs, anxiolytiques)<br>- Femme ne présentant pas de trouble de la personnalité ou de pathologie psychiatrique évolutive<br>- Femme ne présentant pas de contre-indication à la réalisation d'une IRM (claustrophobie, objet métallique dans le corps)<br>- Femme n'ayant pas de problème de vue non corrigé
**Description du protocole / plan expérimental**	Les participantes (2 sous-groupes de patientes et 1 groupe de volontaires indemnes de cancer, de sexe féminin et de même âge) réalisent 3 évaluations successives (T1 : baseline, T2 : à 6 mois, T3 : après stimulation vestibulaire galvanique). Les évaluations comprennent :<br>- IRMa et IRMf au repos et en activation avec la tâche ANT (uniquement à T1 et T2)<br>- Mesures biologiques et physiologiques<br>- Tâche de mémoire spatiale<br>- Batterie cognitive<br>- Questionnaires (sommeil, qualité de vie)
**Nombre de patients nécessaires**	• 25 patientes traitées par chimiothérapie pour un cancer du sein localisé opéré<br>• 25 patientes non traitées par chimiothérapie pour un cancer du sein localisé opéré<br>• 25 femmes volontaires indemnes de cancer du sein de même âge<br><br>Soit 50 patientes au total et 75 participantes au total

**Centres participants**	Centre François Baclesse (recrutement des patientes)<br>Unité 1077 (recrutement des sujets sains)
**Durée de l'étude**	48 mois

2 LISTE DES ABRÉVIATIONS

ANT: Attentional Network Test

BDI: Beck Depression Inventory

Brief COPE: Brief Coping Orientation to Problems Experienced

BPI: Brief Pain Inventory

CAR: Cortisol Awakening Response

FACIT-F: Functional Assessment of Chronic Illness Therapy – Fatigue

FACT-Cog: Functional Assessment of Cancer Therapy - Cognitive Function

FACT-G: The Functional Assessment of Cancer Therapy - General

HVLT: Hopkins Verbal Learning Test

IPAQ: International Physical Activity Questionnaire

ISI: Index de Sévérité de l'Insomnie

KSS : Karolinska Sleepiness Scale

MDC: Mémoire des chiffres

MFI-20: Multidimentional Fatigue Inventory

MoCA: Montreal Cognitive Assessment

Oth: Comments from Others

PCA: Perceived Cognitive Abilities

PCI: Perceived Cognitive Impairment

PCL-5: Posttraumatic Stress Disorder Checklist for DSM-5 (Diagnostic and Statistical Manuel of mental disorders, 5th version)

PSQI: Index de Qualité du Sommeil de Pittsburgh

PTGI: Post-Traumatic Growth Inventory

QoL: Quality of Life

STAI-Y: Inventaire d'anxiété état-trait

SVG: Stimulation Vestibulaire Galvanique

TMT: Trail Making Test

vm: Virtual Meter

3 SCHÉMA DE L'ÉTUDE

Patientes traitées par chimiothérapie adjuvante ***(Groupe CHI)*** :

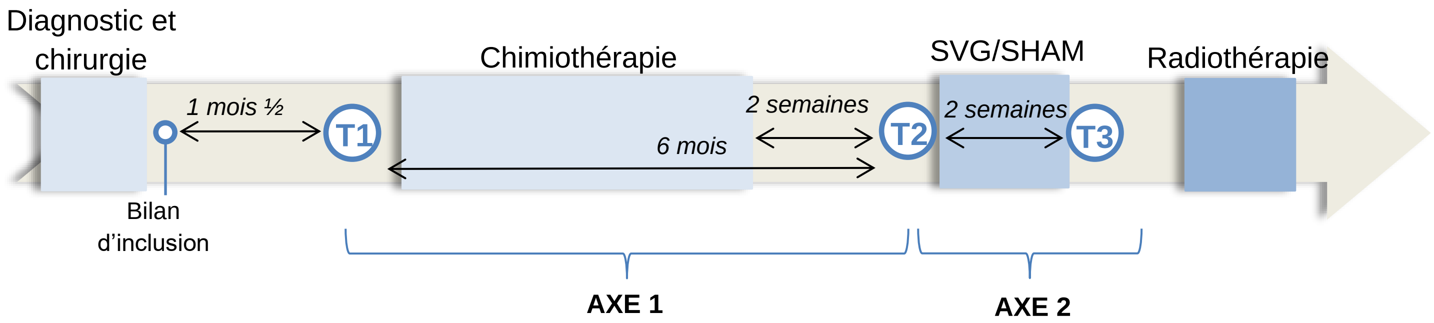

Patientes non traitées par chimiothérapie adjuvante ***(Groupe NCH)*** :

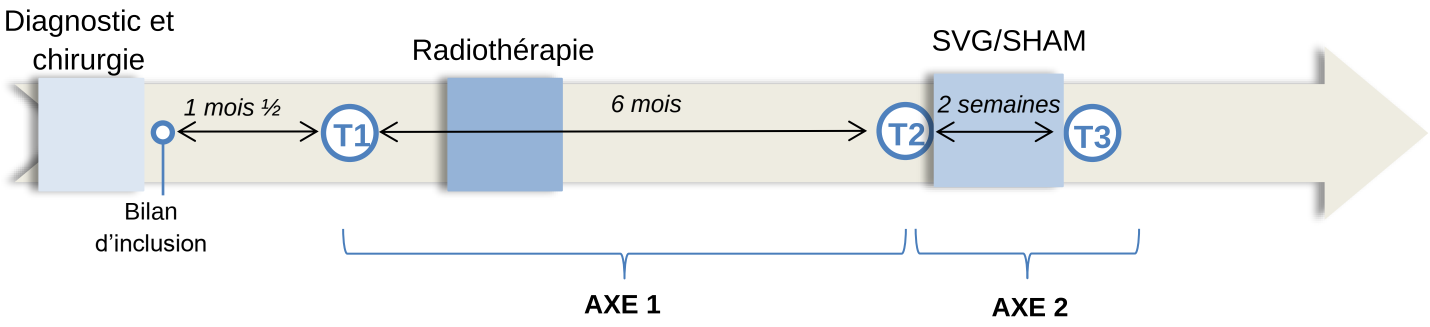

Femmes volontaires indemnes de cancer ***(Groupe CTL)*** :

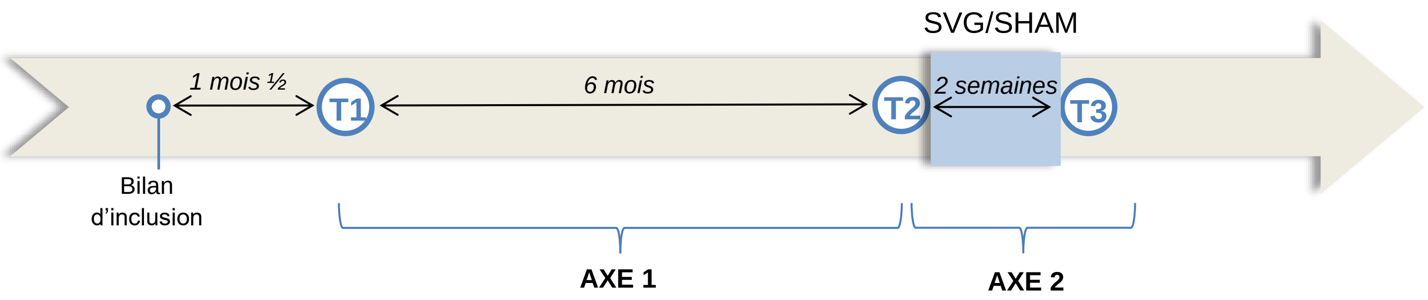

Figure 1 : Schéma de l'étude en fonction du groupe

Note. *SVG : Stimulation Vestibulaire Galvanique, SHAM, stimulation non effective*

4 RÉCAPITULATIF DES EXAMENS À RÉALISER

Tableau 1 : Récapitulatif des évaluations proposées aux participantes aux différents temps de l'étude

Temps<br>Évaluations	Inclusion	**T1**<br>Dans le mois suivant l'inclusion et avant le début du traitement (chimiothérapie ou radiothérapie)	**T2**<br>6 mois après T1	**T3**<br>2 semaines après T2
Signature du consentement éclairé	•			
Plainte de sommeil et apnée du sommeil (questionnaires) :<br>- ISI<br>- Questionnaire de Berlin	•			
Qualité et physiologie du sommeil et des rythmes circadiens<br>- Questionnaires (domicile) : ISI, PSQI, Horne et Ostberg<br>- Actimétrie et agenda du sommeil *(sur les 2 semaines précédant chaque temps d'évaluation T1, T2 et T3)*<br>- Polysomnographie<br>- Cortisol salivaire diurne<br>- CAR et cortisol pré-post IRM		•	•	•
Connectivité fonctionnelle et structures corticales<br>- IRMa<br>- IRMf au repos<br>- IRMf en activation avec tâche attentionnelle (ANT)<br>- Physiologie associée à l'IRM (respiration, pléthysmographie)<br>- Questionnaire de débriefing post-IRM		•	•	
Tâche de mémoire spatiale		•	•	•
Efficience cognitive globale : MoCA		•		
Batterie cognitive<br>- HVLT<br>- Subtest Mémoire Spatiale (MEM-III)<br>- Double tâche de Baddeley<br>- N-Back<br>- d2-R		•	•	•

- TMT A et B<br>- Fluences orthographiques<br>- Stroop				
Qualité de vie (questionnaires)<br>- FACT-G (domicile, patientes uniquement)<br>- FACIT-F (domicile, patientes uniquement)<br>- MFI-20 (domicile)<br>- FACT-Cog (domicile)<br>- KSS<br>- BPI<br>- IPAQ<br>- BDI-II<br>- STAI-Y – état et trait<br>- PCL-5 (patientes uniquement)<br>- PSS<br>- Brief COPE – dispositionnel modifié « avant la suspicion de cancer » (patientes uniquement, T1 uniquement) et dispositionnel<br>- PTGI (patientes uniquement)		•	•	•

Note. *ISI : Index de Sévérité de l'Insomnie ; CAR : Cortisol Awekening Response ; ANT : Attentional Network Test ; MoCA : Montreal Cognitive Assessment ; HVLT : Hopkins Verbal Learning Test ; MEM-III : Échelle Clinique de mémoire de Wechsler – 3ème edition ; TMT : Trail Making Test ; PSQI : Index de Qualité du Sommeil de Pittsburgh ; FACT-G : Functional Assessment of Cancer Therapy – General ; FACIT-F : Functional Assessment of Chronic Illness Therapy – Fatigue ; MFI-20 : Multidimensional Fatigue Inventory ; FACT-Cog : Functional Assessment of Cancer Therapy – Cognitive Function ; KSS : Karolinska Sleepiness Scale ; BPI : Brief Pain Inventory ; IPAQ : International Physical Activity Questionnaire ; BDI-II : Beck Depression Inventory, 2nd edition ; STAI-Y : Inventaire d'anxiété état-trait ; PSS : Perceived Stress Scale ; PCL-5 : Posttraumatic Stress Disorder Checklist for DSM-5 ; Brief COPE : Brief Coping Orientation to Problems Experienced ; PTGI : Post-Traumatic Growth Index*

5 JUSTIFICATION SCIENTIFIQUE DE L'ÉTUDE

Les patientes traitées pour un cancer du sein se plaignent fréquemment de troubles du sommeil, environ 40% d'entre elles. Parmi les troubles du sommeil rencontrés par les patientes, l'insomnie constitue la plainte la plus fréquente. La prévalence de plainte d'insomnie est plus importante chez les patientes atteintes d'un cancer du sein en comparaison à d'autres types de cancer, et est également plus élevée que dans la population générale (entre 20% et 70% chez les patientes atteintes d'un cancer du sein *vs* 30% dans la population générale) [1,2]. Une étude récente indique que la plainte de sommeil concerne 25% des patientes avant même le diagnostic, et 46% (dont 18% se plaignant d'insomnie) au moment du diagnostic, montrant l'impact négatif de l'annonce de la pathologie sur la qualité subjective du sommeil, et notamment sur des symptômes évocateurs d'insomnie [3]. Pourtant, les informations objectives d'une modification de la structure du sommeil dans le cancer du sein restent peu nombreuses et ne permettent pas de conclure (voir [4], pour revue). En particulier, les études précédentes se sont intéressées seulement aux effets de la chimiothérapie et ne comportaient pas toujours de groupe contrôle, limitant la portée de leurs résultats. Les liens avec les processus cognitifs et psychopathologiques, ainsi que les mécanismes sous-jacents ne sont pas clairement démontrés dans cette pathologie. Enfin, tenant compte des plaintes des patientes, il apparaît nécessaire de limiter les troubles du sommeil dans le cancer du sein afin d'améliorer la qualité de vie des patientes à l'aide d'approche non-médicamenteuse et faciles à mettre en œuvre.

5.1 ÉTAT ACTUEL DES CONNAISSANCES

Les difficultés de sommeil dans le cancer du sein restent peu étudiées de façon précise et intégrée (i.e. tenant compte de l'ensemble des processus impliqués dans la régulation du sommeil). En effet, le sommeil est intrinsèquement lié aux rythmes circadiens avec une rythmicité circadienne chez l'humain traduite par une alternance spontanée de périodes de sommeil et d'activité sur 24h, c'est-à-dire le rythme activité-repos. Les études précédentes ont montré une dérégulation du rythme activité-repos dans le cancer du sein avant et pendant la chimiothérapie [5,6] mais également chez des patientes non traitées par chimiothérapie [7]. Parmi ces études, seules trois d'entre elles, dont une issue de nos travaux, comportaient un groupe contrôle sans antécédents de cancer [8,9]. Les résultats montrent un temps total de sommeil plus long ainsi qu'un allongement de la durée des siestes au cours de la chimiothérapie par rapport à la période avant l'initiation du traitement [10–12] et par rapport aux sujets contrôles [8,9]. En outre, le rythme activité-repos semble moins important (i.e. moins ample) chez les patientes avec un cancer du sein n'ayant pas reçu de chimiothérapie par rapport à des sujets sans antécédents de cancer [7]. À l'inverse, Li et collaborateurs ont montré que l'initiation de la chimiothérapie, comparativement aux évaluations pré-traitements et à la fin de la chimiothérapie, était associée à un temps de sommeil moins long ainsi qu'à des éveils plus nombreux et une qualité de sommeil moindre [13]. Beck et collaborateurs rapportent également un temps de sommeil plus court lors de la première nuit après l'initiation de la chimiothérapie par rapport à avant et après la période de traitement [14]. Enfin, Kuo et collaborateurs n'ont pas mis en évidence de différence entre les évaluations avant et au cours de la chimiothérapie [15]. Les résultats concernant les effets de la chimiothérapie sur les paramètres du sommeil mesurés en actimétrie sont donc très variables et ne permettent pas de conclure de façon définitive quant à des altérations du sommeil telles que mesurées en actimétrie [4]. Une revue de la littérature publiée en 2015 [16] rapportait cependant que la chimiothérapie pourrait

accentuer des difficultés de sommeil déjà présentes avant l'initiation des traitements dans le cancer du sein.

Contrairement aux études en actimétrie, les études en polysomnographie (PSG), *« gold-standard »* de l'évaluation du sommeil, restent très rares. De ce fait, les altérations de la structure du sommeil dans cette pathologie ne sont pas clairement décrites. En outre, la PSG permet de quantifier des mesures fines associées à la structure du sommeil et à la consolidation mnésique. Son utilisation permet donc une meilleure compréhension des modifications du sommeil que l'actimétrie qui reste une approche indirecte pour quantifier la qualité et la quantité de sommeil. Les études précédentes réalisées en PSG suggèrent des altérations de la qualité et de la quantité de sommeil suite aux traitements par chimiothérapie. Parker et collaborateurs ont comparé l'architecture du sommeil de patients traités pour un cancer non-cérébral à un stade avancé (cancers de stade 3 ou 4), dont 32 patientes traitées pour un cancer du sein (28% de la cohorte) [17]. Les résultats montrent chez ces patientes une efficacité du sommeil et des durées de stade de sommeil réduites par rapport aux normes établies par Williams et collaborateurs auprès de participants sains [18]. Roscoe et collaborateurs [19] ont révélé qu'après la fin de la chimiothérapie, les patientes dormaient plus qu'avant le début de la chimiothérapie. Ce résultat pourrait s'expliquer par un sommeil de mauvaise qualité au départ en raison du stress associé au diagnostic du cancer et à l'appréhension de la chimiothérapie, ou par une accumulation de fatigue et de manque de sommeil pendant le traitement, entraînant une compensation à l'issue du traitement.

Ces études mettent en évidence des changements dans la macrostructure du sommeil des patientes traitées pour un cancer du sein mais elles présentent plusieurs limites empêchant de comprendre de manière précise l'impact du cancer et des traitements sur le sommeil. L'absence de groupe témoin dans plusieurs de ces études est la principale limite. En effet, la présence d'un groupe témoin sans antécédents de cancer et d'un groupe sans traitement permet de déterminer si les changements dans l'architecture du sommeil sont secondaires au cancer lui-même ou au traitement. De plus, l'inclusion de populations hétérogènes en termes d'âge et de types de cancer pourrait avoir limité la puissance statistique des résultats des études et empêché de révéler des modifications de l'architecture du sommeil. En outre, aucune étude n'a évalué les modifications du sommeil avant l'initiation des traitements par chimiothérapie. La PSG permet en outre d'obtenir des informations fines sur la macro- et la micro-structure du sommeil, donnant la possibilité de faire des liens avec les performances cognitives et de détecter de potentiels troubles du sommeil tels que les apnées. De nouvelles études, plus homogènes en termes de critères d'éligibilité, mais également longitudinales, sont nécessaires afin de déterminer la présence de troubles du sommeil chez des patientes présentant un cancer du sein. Une meilleure compréhension de ces troubles du sommeil permettra, en outre, de déterminer leur éventuelle contribution dans la survenue des déficits cognitifs et psychopathologiques.

Les patientes atteintes d'un cancer du sein présentent des troubles de la mémoire épisodique, mais également des fonctions exécutives. Ces dernières sont particulièrement sollicitées dans une forme particulière de mémoire épisodique, la mémoire spatiale, jusqu'ici peu étudiée dans le cancer du sein. Ce type de mémoire permet de se souvenir d'un trajet ou de retrouver des objets dans divers lieux ; elle est donc essentielle au quotidien. De plus, elle repose sur l'intégrité de l'hippocampe, une région qui semble affectée chez les femmes avec un cancer du sein [8]. Les troubles du sommeil sont également connus pour altérer les fonctions exécutives impliquant le cortex préfrontal [9] et les mécanismes de contrôle cognitif qui en dépendent. Ces mécanismes de contrôle cognitif permettent la régulation du comportement lors de situations complexes, notamment après un événement stressant. Dans le

cancer du sein, les troubles du sommeil pourraient avoir un impact négatif sur les mécanismes de contrôle, induisant une moindre résistance au stress.

Au-delà des effets bénéfiques du sommeil sur les processus cognitifs, notamment mnésiques et sur les fonctions exécutives [20,21], le sommeil joue un rôle important dans la réponse au stress chez le sujet sain et dans diverses pathologies [22]. L'annonce du cancer, de même que la situation créée par la pathologie, constituent une source de stress majeure. Cependant, les patientes, comme cela est le cas dans la population générale, ne réagissent pas de façon homogène à ces situations. Les troubles du sommeil pourraient influencer la façon dont les patientes réagissent à des situations de stress, comme celles liées à leur parcours de soin. Il est nécessaire de comprendre dans quelle mesure le sommeil influence les processus psychopathologiques dans le cancer du sein afin d'accompagner au mieux les patientes dans leur parcours en améliorant les soins de support.

5.2 PRISE EN CHARGE DÉJÀ EXISTANTES ET LEURS LIMITES

Les troubles du sommeil et les troubles des rythmes circadiens sont fréquents dans le cancer du sein et il apparaît nécessaire de les prendre en charge. Dans cet objectif, les approches non-médicamenteuses proposées actuellement sont les thérapies cognitivo-comportementales et l'activité physique. Cependant, bien que des effets positifs aient été rapportés sur l'humeur, la qualité de vie et la plainte de sommeil, leurs effets bénéfiques sur la qualité et la quantité de sommeil mesurées par PSG dans le cancer du sein ne sont pas clairement établis [23,24]. De plus, ces approches ne ciblent pas spécifiquement les troubles du sommeil et/ou des rythmes circadiens. Des résultats prometteurs suggèrent que le système vestibulaire pourrait être impliqué dans la régulation et la resynchronisation circadienne [25–27]. De même, des études récentes ont montré les effets positifs d'une stimulation du système vestibulaire *via* le balancement pendant le sommeil sur la qualité de sommeil chez les sujets sains. Enfin, des résultats récents de l'équipe COMETE (thèse de doctorat de Florane Pasquier, Dir : Prof. Gaëlle Quarck) suggèrent que la stimulation du système vestibulaire, notamment galvanique, permettrait d'agir sur les rythmes [28]. L'utilisation d'une stimulation du système vestibulaire apparaît donc particulièrement appropriée pour améliorer spécifiquement le sommeil et resynchroniser les rythmes dans le cancer du sein. Pour cela, il serait possible d'utiliser la stimulation vestibulaire galvanique (SVG), fondée sur la stimulation de l'organe vestibulaire périphérique, à travers l'activation des afférences vestibulaires, en appliquant un courant électrique entre les deux mastoïdes. La SVG est utilisée depuis de nombreuses années afin de stimuler le système vestibulaire de façon indolore et sécurisée [29], mais des études plus récentes ont également démontré la faisabilité et les effets positifs de la SVG sur la cognition [30–32] et sur les niveaux d'anxiété [33] chez des participants sains. La SVG ne présente pas de contre-indication particulière et pourrait donc être proposée à l'ensemble des participantes.

5.3 DESCRIPTION DE LA NOUVELLE PRISE EN CHARGE PROPOSÉE

La SVG sera réalisée grâce à un dispositif disponible dans l'U1075 (Caen, Dir : Prof. Thomas Freret), cette équipe étant spécialisée dans la remédiation en lien avec les rythmes biologiques, notamment à travers la stimulation vestibulaire. L'appareil utilisé est distribué par Soterix médical et dispose d'un marquage CE (https://soterixmedical.com/research/vestibular). Ce matériel permet de stimuler les afférences vestibulaires de façon autonome et pré-régulée : ainsi, le niveau de stimulation est pré-programmé et ne peut pas être modifié sans être administrateur de l'appareil, assurant une précision et une sécurité de la stimulation. Afin de déterminer un protocole de stimulation le plus juste et le plus

efficace possible, une évaluation du dispositif est en cours dans notre laboratoire chez des participants indemnes de cancer dans le cadre d'un stage de Master 2 et aucun effet secondaire n'a été rapporté. En outre, l'unité COMETE (Dir : Thomas Freret, travaux du Prof. Gaëlle Quarck) réalise actuellement un protocole (projet VELOCCE) chez des personnes âgées sans pathologies. De même, aucun effet secondaire n'a été déclaré par les participants. L'ensemble de ces résultats démontrent l'innocuité, la faisabilité et la facilité d'utilisation du dispositif de SVG de la marque Soterix qui sera utilisé dans le cadre du protocole ICANSLEEP. Le protocole de stimulation (stimulation effective, SVG ou non effective, SHAM) sera réalisé à distance, cinq fois par semaine au cours de la matinée pendant deux semaines, à l'aide d'une plateforme de visio-conférence accessible de façon sécurisée disponible sur le réseau de l'Université de Caen. Les participantes pourront s'y connecter *via* un ordinateur aux horaires et jours indiqués par les expérimentateurs. Une fois connectées, elles seront en visio-conférence avec un(e) professionnel(le) de l'équipe de recherche qui vérifiera la bonne position des électrodes et notera le comportement des participantes. Ces visioconférences permettront également de s'assurer d'une homogénéité des conditions de réalisation entre les séances et entre les participantes.

La thèse de Florane Pasquier (encadrée par Gaëlle Quarck), récemment soutenue a permis de montrer la tolérance et les effets bénéfiques de la stimulation galvanique sur l'anxiété et sur les rythmes circadiens. Des publications utilisant le matériel de SVG de la marque Soterix ont été publiées récemment, attestant de son innocuité et de la faisabilité d'utilisation [34,35]OBJECTIFS DE L'ÉTUDE

5.4 OBJECTIF PRINCIPAL

Ce projet s'articule autour de deux axes, ayant chacun un objectif principal.

Axe 1 : Caractériser le sommeil de patientes suivies pour un cancer du sein avant et après chimiothérapie adjuvante par rapport à des patientes non traitées par chimiothérapie et à des femmes sans antécédents de cancer.

Axe 2 : Explorer l'effet régulateur de la stimulation vestibulaire galvanique (SVG) sur les rythmes circadiens chez les patientes suivies pour un cancer du sein localisé par rapport à une stimulation SHAM (non effective).

5.5 OBJECTIFS SECONDAIRES

Axe 1 :

1) **a)** Évaluer les effets du cancer et de la chimiothérapie sur le sommeil et les rythmes circadiens
   b) Déterminer si la présence de troubles du sommeil est associée à une altération des rythmes circadiens
2) Évaluer les effets du cancer et de la chimiothérapie sur les capacités cognitives et leurs corrélats neuro-fonctionnels
3) Évaluer les effets du cancer et de la chimiothérapie sur la structure corticale
4) Évaluer les effets du cancer et de la chimiothérapie sur la connectivité fonctionnelle au repos
5) Évaluer le lien existant entre les troubles du sommeil avec :
   a) Les capacités cognitives et leurs corrélats neuro-anatomiques
   b) Les systèmes physiologiques du stress
   c) La qualité de vie

Axe 2 :

1) Évaluer les effets de la SVG sur :
   a) Les rythmes circadiens
   b) La mémoire spatiale
   c) Les systèmes physiologiques du stress
2) Évaluer les effets de la resynchronisation des rythmes sur la qualité de sommeil
3) Déterminer si l'amélioration du sommeil est accompagnée de meilleures performances cognitives ainsi que d'une meilleure qualité de vie

6 HYPOTHÈSE DE RECHERCHE ET RÉSULTATS ATTENDUS

Axe 1 :

1) **a)** Le sommeil et les rythmes circadiens seraient altérés chez les patientes avant chimiothérapie par rapport aux sujets indemnes de cancer. La chimiothérapie exacerberait ces effets par rapport au groupe sans chimiothérapie

    - Pour le sommeil, plus d'éveils de plus d'une minute et une moindre qualité de sommeil sont attendus
    - Pour les rythmes circadiens, il est attendu que le rythme veille/sommeil et le rythme du cortisol soient moins amples

   b) L'altération des rythmes entrainerait des effets délétères sur la qualité de sommeil

2) Les capacités cognitives seraient plus altérées après qu'avant la chimiothérapie, alors qu'aucun déclin n'est attendu chez les patientes non traitées par chimiothérapie et les sujets sains. Plus précisément ce déclin devrait concerner :

    - La mémoire spatiale et la mémoire épisodique
    - La mémoire de travail
    - Les fonctions exécutives et les capacités attentionnelles
    - La connectivité fonctionnelle au cours de la tâche d'ANT (Attentional Network Test), en particulier au sein des réseaux de l'orientation et du contrôle cognitif

3) Après chimiothérapie, la structure corticale devrait être altérée, notamment dans les régions impliquées dans les capacités cognitives sus-décrites (c-à-d, hippocampe, régions frontales, cervelet Crus) par rapport à avant chimiothérapie, et par rapport aux patientes non traitées par chimiothérapie et aux sujets sains
4) Avant chimiothérapie, pour les deux groupes de patientes, la connectivité fonctionnelle au repos devrait être modifiée dans sa dynamique, en lien avec des ruminations plus importantes en comparaison au groupe de sujets sains.
5) **a)** Les troubles du sommeil (indépendamment des effets de l'anxiété et de la dépression) seraient liés aux capacités cognitives et à leurs corrélats neuro-anatomiques, plus précisément :
   - Aux performances en mémoire épisodique et en mémoire spatiale
   - À la structure de la substance blanche et à la connectivité fonctionnelle

   b) Les troubles du sommeil seraient également associés à une hyperactivité des systèmes physiologiques du stress

   c) Ces troubles du sommeil seraient associés à une moindre qualité de vie

Axe 2 :

1) La SVG permettrait :
   a) De resynchroniser les rythmes circadiens (c-à-d l'acrophase) et d'amplifier le rythme veille/sommeil au fur et à mesure de l'intervention et le rythme du cortisol à la fin de l'intervention
   b) Une amélioration des performances en mémoire spatiale par rapport à T2
   c) Une diminution de l'hyperactivité des systèmes physiologiques du stress
2) La resynchronisation des rythmes permettrait une meilleure qualité de sommeil
3) Cette amélioration de la qualité de sommeil permettrait une meilleure récupération cognitive et une meilleure qualité de vie suite au parcours de soin par rapport à l'absence de SVG

7 CRITÈRES DE JUGEMENT

7.1 CRITÈRE PRINCIPAL

Axe 1 : Le critère principal retenu est l'efficacité de sommeil (ratio temps total de sommeil/temps passé au lit) et le nombre d'éveils de plus d'une minute. Ces données sont issues de la polysomnographie.

Axe 2 : Le critère principal retenu est l'amplitude (correspondant à la moitié de la variation maximale du rythme considéré) et l'acrophase (c-à-d l'heure à laquelle l'activité ou le cortisol est le plus important). L'évolution du rythme veille/sommeil sera quantifiée au cours des 2 semaines de l'intervention et seront issues de l'actimétrie. L'évolution du rythme du cortisol sera mesurée à l'aide des recueils salivaires obtenus à T2 et T3.

7.2 CRITÈRES SECONDAIRES

Tableau 2 : Critères secondaires associés aux objectifs secondaires

**Axe 1**	
**Objectifs**	**Examens :** mesures associées
**1) a) b)**	- **Actimétrie :** amplitude et acrophase du rythme<br>- **Agenda du sommeil :** scores<br>- **Cortisol diurne :** taux de cortisol salivaire<br>- **Polysomnographie : efficacité du sommeil et nombre d'éveils**<br>- **Questionnaires ISI, PSQI : scores**
**2)**	- **Tests neuropsychologiques :** scores<br>- **Tâche de mémoire spatiale :** scores<br>- **IRMf activation (tâche ANT) :** signal BOLD relatif à la tâche (connectivité effective)
**3)**	- **IRMa :** structures corticales (substances blanche et grise)
**4)**	- **IRMf au repos :** fluctuations dynamiques du signal BOLD (connectivité fonctionnelle) corrigées des facteurs de confusions cardiorespiratoires<br>- **Questionnaire de débriefing post-IRM :** scores
**5) a)**	- **Polysomnographie :** efficacité du sommeil et nombre d'éveils<br>- **Questionnaires ISI, PSQI :** scores<br>- **HVLT :** scores<br>- **Tâche de mémoire spatiale :** scores<br>- **IRMa :** structures corticales (substance blanche)<br>- **Questionnaires STAI, BDI-II :** scores
**5) b)**	- **Polysomnographie :** efficacité du sommeil et nombre d'éveils<br>- **Questionnaires ISI, PSQI :** scores<br>- **CAR et cortisol pré- post- IRM :** taux de cortisol salivaire
**5) c)**	- **Polysomnographie :** efficacité du sommeil et nombre d'éveils<br>- **Questionnaires ISI, PSQI :** scores<br>- **Questionnaires (qualité de vie) :** scores
**Axe 2**	
**Objectifs**	**Examens :** mesures associées
**1) a)**	- **Actimétrie :** amplitude et acrophase du rythme<br>- **Agenda du sommeil :** scores<br>- **Cortisol diurne :** taux de cortisol salivaire
**1) b)**	- **Tâche de mémoire spatiale :** scores
**1) c)**	- **CAR et cortisol pré- post- IRM :** taux de cortisol salivaire
**2)**	- **Actimétrie :** amplitude et acrophase du rythme<br>- **Agenda du sommeil :** scores<br>- **Polysomnographie :** efficacité du sommeil et nombre d'éveils<br>- **Questionnaires ISI, PSQI :** scores
**3)**	- **Polysomnographie :** efficacité du sommeil et nombre d'éveils<br>- **Questionnaires ISI, PSQI :** scores<br>- **Tests neuropsychologiques :** scores<br>- **Questionnaires (qualité de vie) :** scores

Note. *ISI : Index de Sévérité de l'Insomnie ; PSQI : Index de Qualité du Sommeil de Pittsburgh ; ANT : Attentional Network Test ; HVLT : Hopkins Verbal Learning Test ; CAR : Cortisol Awakening Response ; BDI-II : Beck Depression Inventory ; STAI: Inventaire d'anxiété état-trait*

8 PLAN DE L'ÉTUDE

8.1 MÉTHODOLOGIE

Le projet ICANSLEEP consiste en une étude longitudinale bicentrique.

Les données de 25 patientes suivies pour un cancer du sein localisé et traitées par chimiothérapie et seront comparées à celles d'un groupe de 25 patientes non traitées par chimiothérapie et d'un groupe constitué de 25 sujets volontaires indemnes de cancer (groupes témoins), appariés en sexe, âge et en nombre d'années d'études (Échelle de Barbizet).

Les différentes évaluations seront effectuées comme suit :

- A T1 : Environ 1 mois et demi après la chirurgie et avant le début du traitement par chimiothérapie pour le groupe CHI et avant le début du traitement par radiothérapie pour le groupe NCH,
- A T2 : après un délai d'environ 6 mois, dans les 2 semaines suivant la fin de la chimiothérapie pour le groupe CHI, ou à distance de la radiothérapie pour le groupe NCH,
- A T3 : après une intervention de deux semaines par SVG versus une stimulation non effective (dite SHAM). Afin d'évaluer l'effet de la SVG, les patientes seront assignées soit au groupe réalisant la SVG effective soit au groupe SHAM (tirage au sort). La stimulation SHAM consiste à utiliser l'appareil mais en recevant un courant de très basse fréquence n'ayant aucun effet sur le système vestibulaire. Cette approche est déjà utilisée et validée dans l'unité COMETE (Caen, Prof. Gaëlle Quarck) avec laquelle nous collaborons. Les participantes volontaires indemnes de cancer réaliseront toutes la SVG.

8.2 DURÉE DE L'ÉTUDE

La durée totale de l'étude est de 48 mois dont une période de recrutement de 40 mois.

La durée de la période de suivi des participantes est de 8 mois à partir du bilan d'inclusion.

8.3 SÉLECTION DES PARTICIPANTES

Pour chacun des deux groupes, les patientes (25 patientes traitées par chimiothérapie et 25 patientes non-traitées par chimiothérapie) seront recrutées au Centre de Lutte Contre le Cancer François Baclesse de Caen. Les patientes non traitées par chimiothérapie seront d'âge et de niveau d'études équivalents aux patientes traitées par chimiothérapie.

Un groupe de 25 femmes volontaires sans antécédent de cancer, d'âge et de niveau d'études équivalents aux patientes sera constitué, par appel à volontaires (cf section « Inclusion des femmes indemnes de cancer » ci-après). Ce recrutement sera effectué au sein de l'unité 1077.

8.3.1 Critères d'inclusion des patientes

- Patiente âgée de 45 à 65 ans
- Patiente atteinte d'un cancer du sein localisé opéré devant ou non débuter un traitement par chimiothérapie adjuvante
- Patiente de niveau d'études 3 « fin d'études primaires » (échelle de Barbizet)
- Patiente maîtrisant la langue française

- Patiente ayant signé le consentement pour participer à l'étude
- Patiente présentant une plainte de sommeil (ISI > 7)
- Patiente ayant accès à internet depuis un ordinateur au domicile

8.3.2 Critères de non inclusion des patientes

- Patiente présentant un cancer métastasique
- Patiente présentant un cancer primitif différent du cancer du sein
- Patiente ayant un antécédent d'atteinte neurologique
- Patiente présentant un syndrome d'apnée du sommeil traité
- Patiente faisant usage de drogue ou ayant une consommation abusive d'alcool (≥3 verres/jour en moyenne et/ou >10 verres /semaine)
- Patiente ayant un traitement non stabilisé depuis au moins 3 mois (hypnotiques, antidépresseurs, anxiolytiques)
- Patiente présentant un trouble de la personnalité ou une pathologie psychiatrique évolutive
- Patiente présentant une contre-indication à la réalisation d'une IRM (claustrophobie ; objets métalliques dans le corps tels que pacemaker, matériel implanté activé par un système électrique, magnétique ou mécanique, clips hémostatiques des anévrysmes intracérébraux ou des artères carotides, implants orthopédiques)
- Patiente ayant un problème de vue non corrigé
- Patiente présentant des altérations cognitives préexistantes au diagnostic du cancer

8.3.3 Critères de sélection des femmes indemnes de cancer

- Femme issue de la population générale appariée en âge et en niveau d'études (échelle de Barbizet) aux patientes
- Femme de niveau d'études 3 « fin d'études primaires » minimum (échelle de Barbizet)
- Femme maîtrisant la langue française
- Femme ayant signé le consentement pour participer à l'étude
- Femme ne présentant pas d'antécédent de cancer
- Femme présentant une plainte de sommeil (ISI > 7)
- Femme ne présentant pas d'apnée du sommeil traitée
- Femme ayant accès à internet depuis un ordinateur au domicile
- Femme ne travaillant pas en horaires postés
- Femme ne présentant pas d'antécédent d'atteinte neurologique
- Femme ne faisant pas usage de drogue ou n'ayant pas une consommation abusive d'alcool (≤3 verres/jour en moyenne et/ou <10 verres /semaine)
- Femme n'ayant pas de traitement non stabilisé depuis au moins 3 mois (hypnotiques, antidépresseurs, anxiolytiques)
- Femme ne présentant pas de trouble de la personnalité ou de pathologie psychiatrique évolutive
- Femme ne présentant pas de contre-indication à la réalisation d'une IRM (claustrophobie ; objets métalliques dans le corps tels que pacemaker, matériel implanté activé par un système électrique, magnétique ou mécanique, clips hémostatiques des anévrysmes intracérébraux ou des artères carotides, implants orthopédiques)
- Femme n'ayant pas de problème de vue non corrigé

8.4 DÉROULEMENT DE L'ÉTUDE

8.4.1 Procédure d'inclusion

8.4.1.1 Inclusion des patientes

L'étude sera proposée par les oncologues et/ou radiothérapeutes aux patientes répondant aux critères d'éligibilité (groupes avec et sans chimiothérapie adjuvante). Une note d'information et un formulaire de consentement éclairé leur seront remis (voir Annexe 1). Les patientes auront un délai de réflexion de la durée de leur choix.

Après le recueil de l'accord de la patiente par signature du consentement de l'étude, les critères de sélection seront vérifiés avant l'inclusion dans l'essai.

Les examens spécifiques à l'étude demandés avant l'inclusion (bilan d'inclusion) seront réalisés après signature du consentement et avant inclusion.

L'inclusion sera enregistrée sur le logiciel dédié à l'étude via un portail internet.

Un numéro d'identification sera attribué à la patiente et servira tout au long de l'étude.

8.4.1.2 Inclusion des femmes indemnes de cancer

Les volontaires sains seront recrutés par connaissances, associations, par l'intermédiaire de l'Espace Rencontre et Information [ERI] du Centre François Baclesse, ou encore par d'autres groupes d'individus. Après un contact téléphonique préalable et une brève présentation de l'étude, les volontaires sains seront invitées au sein de notre unité de recherche afin de réaliser la visite d'inclusion. Durant cette visite, l'investigateur présentera le but et les modalités de cette étude, puis remettra aux volontaires une note d'information et un formulaire de consentement (voir Annexe 2). Les participantes témoins auront un délai de réflexion de la durée de leur choix afin de prendre leur décision de participer ou non au protocole.

Les femmes indemnes de cancer seront appariées en âge et en niveau d'études (échelle de Barbizet) aux patientes.

Après accord écrit du sujet, vérification de tous les critères de sélection, et avant de débuter l'étude, l'inclusion sera enregistrée sur le logiciel dédié à l'étude via un portail internet.

Un numéro d'identification sera attribué à la femme volontaire et servira tout au long de l'étude.

8.4.2 Évaluations de l'étude

Un tableau récapitulatif des investigations demandées et ainsi qu'un schéma précisant les différents temps d'évaluation des participantes sont présents en début de document (*Figure 1*).

La première évaluation sera réalisée dans un délai d'un mois suivant l'inclusion et avant le début des traitements par chimiothérapie (groupe CHI) ou radiothérapie (groupe NCH) selon la disponibilité des participantes.

Tout au long des sessions évaluatives, les participantes seront accompagnées par l'équipe de l'UMR 1077, composée d'une neuropsychologue, d'un technicien du sommeil et d'un doctorant.

En amont des temps d'évaluation, des données d'actimétrie seront recueillies auprès des participantes 2 semaines avant chaque temps d'évaluation (T1, T2 et T3). Pour ce faire, un actimètre accompagné d'un agenda de sommeil avec des explications relatives à leur utilisation seront fournis aux participantes soit *de visu,* soit à distance (envoi de l'actimètre et de l'agenda de sommeil par voie postale et visio-conférence).

Les évaluations proposées aux participantes durant les différents temps de l'étude (T1, T2 et T3) sont présentées dans les figures suivantes (*Figure 2* et *Figure 3*). Chaque séquence évaluative sera effectuée en 2 demi-journées (après-midi et matin) séparées d'une nuit de sommeil afin de permettre l'examen de polysomnographie.

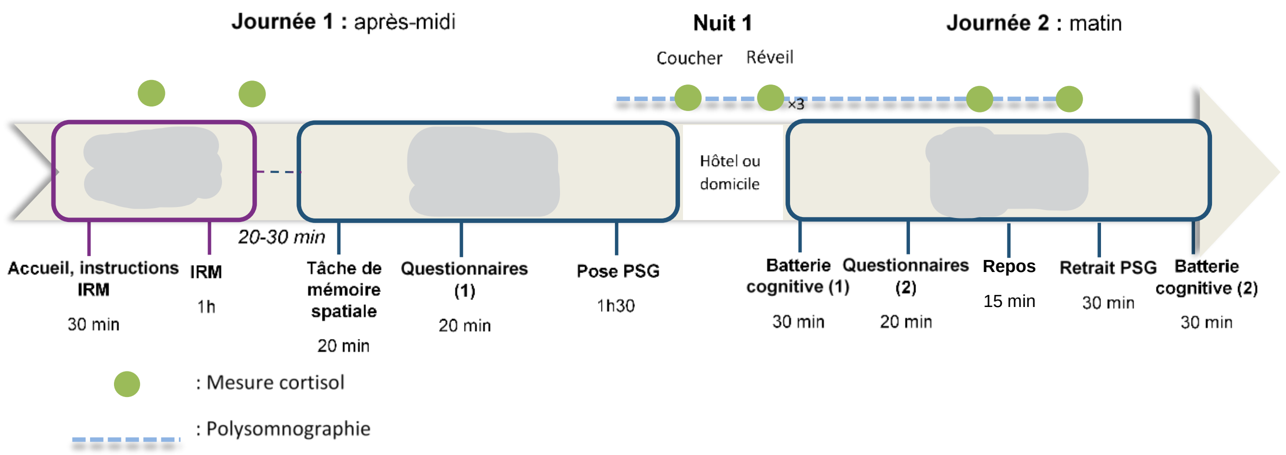

Figure 2 : Évaluations proposées aux participantes à T1 et T2

Note. *L'actimétrie sera fournie 2 semaines avant T1, idem à T2 ; À T1, un entretien préalable aura lieu à l'accueil des participantes (environ 30 min, en plus des instructions IRM) ; À T1, un temps de familiarisation avec le matériel de stimulation vestibulaire galvanique aura lieu avant l'examen IRM (20 min) ; PSG : Polysomnographie*

8.4.3 Bilan d'inclusion

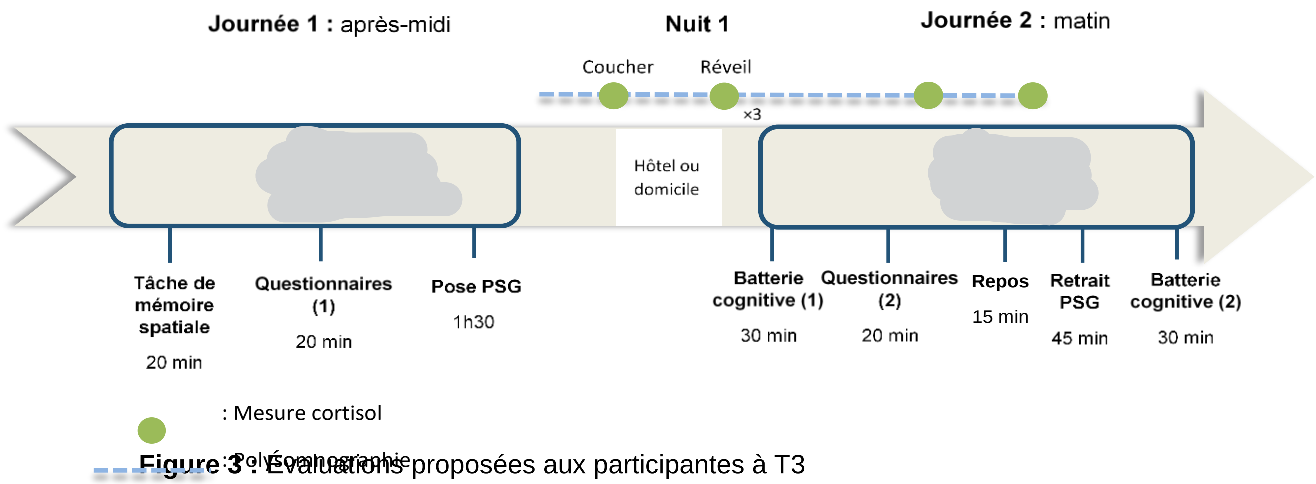

Figure 3 : Évaluations proposées aux participantes à T3

Note. *L'actimétrie sera fournie 2 semaines avant T3 ; PSG : Polysomnographie*

Les participantes ayant signé leur consentement de participation devront effectuer les examens suivants :

- ISI (plainte de sommeil)
- Questionnaire de Berlin (apnées du sommeil)

8.4.4 Bilans T1 et T2

Chez les patientes traitées par chimiothérapie adjuvante (Groupe CHI)

- Le bilan T1 aura lieu après la chirurgie et avant le début de la chimiothérapie
- Le bilan T2 aura lieu 2 semaines après la fin de la dernière cure de chimiothérapie

Chez les patientes non-traitées par chimiothérapie adjuvante (Groupe NCH)

- Le bilan T1 aura lieu après la chirurgie et avant le début de la radiothérapie
- Le bilan T2 aura lieu 6 mois après T1

Chez les participantes volontaires saines (Groupe CTL) :

- Le bilan T1 aura lieu dans les 6 semaines suivant le bilan d'inclusion
- Le bilan T2 aura lieu 6 mois après T1

Pour l'ensemble des participantes de l'étude, les évaluations suivantes seront proposées à T1 et T2 :

- Actimétrie et agenda du sommeil (pendant 2 semaines avant chaque bilan)
- Questionnaires à remplir au domicile (sommeil et qualité de vie) :
    - ISI
    - PSQI
    - Horne et Ostberg
    - FACT-G (patientes uniquement)
    - FACIT-F (patientes uniquement)
    - MFI-20
    - FACT-Cog
- Dosage du cortisol salivaire diurne et CAR (Cortisol Awakening Response)
- IRM (IRMa et IRMf au repos et d'activation avec la tâche ANT + questionnaire de débriefing post-IRM)
- Tâche de mémoire spatiale
- Polysomnographie
- Tests neuropsychologiques :
    - MoCA (T1 uniquement)
    - HVLT
    - Subtest Mémoire Spatiale (MEM-III)
    - Double tâche de Baddeley
    - N-Back
    - d2-R
    - TMT A et B
    - Fluences orthographiques
    - Stroop

- Questionnaires (qualité de vie) :
    - KSS
    - BDI-II
    - BPI
    - STAI – État
    - STAI – Trait
    - PCL-5 (patientes uniquement)
    - PSS
    - Brief-COPE dispositionnel modifié (avant suspicion de cancer) (patientes uniquement, T1 uniquement)
    - Brief COPE dispositionnel
    - PTGI
    - IPAQ
- Évaluation de la fonction vestibulaire (video Head Impulse Test, vHIT)

8.4.5 Bilan T3

Pour l'ensemble des participantes, le bilan suivant sera réalisé à l'issue de la période de SVG ou SHAM (2 semaines) effectuée après T2.

- Actimétrie et agenda du sommeil (entre T2 et T3)
- Questionnaires à remplir au domicile (sommeil et qualité de vie) :
    - ISI
    - PSQI
    - Horne et Ostberg
    - FACT-G (patientes uniquement)
    - FACIT-F (patientes uniquement)
    - MFI-20
    - FACT-Cog
- Dosage du cortisol salivaire diurne et CAR (Cortisol Awekening Response)
- Tâche de mémoire spatiale
- Polysomnographie
- Tests neuropsychologiques :
    - HVLT
    - Subtest Mémoire Spatiale (MEM-III)
    - Double tâche de Baddeley
    - N-Back
    - d2-R
    - TMT A et B
    - Fluences orthographiques
    - Stroop
- Questionnaires (qualité de vie) :
    - KSS

- BDI-II
- BPI
- STAI – État
- STAI – Trait
- PCL-5 (patientes uniquement)
- PSS
- Brief COPE dispositionnel
- PTGI
- IPAQ

- Évaluation de la fonction vestibulaire (video Head Impulse Test, vHIT)

8.5 ARRÊT PRÉMATURÉ DE L'ÉTUDE

L'intervention sera interrompue à tout moment dans les circonstances suivantes :

- Événement intercurrent, non compatible avec la poursuite de l'étude
- Décision de la participante (les données déjà collectées lors de la recherche pourront être conservées et exploitées sauf si le sujet s'y oppose)
- Participante perdue de vue
- Décision de l'investigateur

8.6 MODALITES DE REALISATION DES ÉVALUATIONS DES PARTICIPANTES

Pour T1 et T2, les participantes seront reçues en début d'après-midi au GIP Cyceron (Caen) afin de réaliser les séquences IRM en la présence d'un médecin investigateur.

La poursuite des évaluations se fera au PFRS, situé à proximité immédiate de Cyceron (*Annexe 3*).

À T3, les participantes seront directement reçues par l'équipe au PFRS de Caen pour réaliser l'ensemble des évaluations.

La passation de la tâche de mémoire spatiale, de la batterie cognitive ainsi que les sessions de questionnaires seront effectuées dans une pièce adaptée au sein de l'UMR 1077, avec une psychologue spécialisée en neuropsychologie.

La pose et le retrait de l'équipement de polysomnographie seront effectués par deux techniciens du sommeil spécialisés, dans une pièce dédiée au sein de l'UMR 1077.

Le vHIT sera réalisé aux temps T1, T2 et T3 au PFRS par un technicien formé à la passation de ce test. Les résultats seront interprétés par un médecin spécialiste de la fonction vestibulaire et rattaché au protocole ICANSLEEP.

Le temps de nuit durant lequel les participantes porteront l'équipement de polysomnographie n'est pas comptabilisé dans les durées précédemment décrites. Concernant la nuit séparant les 2 demi-journées d'évaluation, les participantes pourront se rendre à leur domicile ou bien à l'hôtel dont les frais seront pris en charge. À chaque temps de l'évaluation, une partie des questionnaires (sommeil et qualité de vie) sera donnée aux participantes à remplir à leur domicile.

Une indemnité totale de 300 € sera versée à chaque participante en contrepartie des contraintes et du temps investi durant leur participation à l'étude. En cas de sortie prématurée de l'étude, l'indemnisation sera réalisée au prorata de la participation à l'étude soit 100 € par temps d'évaluation réalisé dans son intégralité ou non.

9 OUTILS D'EVALUTATION

9.1 Qualité et physiologie du sommeil et des rythmes circadiens

Les indices de quantité et de qualité du sommeil des participantes seront recueillis non seulement à l'aide de mesures subjectives (auto-questionnaires) mais aussi avec des mesures objectives (actimétrie, PSG).

9.1.1 Auto-évaluations du sommeil et de la typologie circadienne

Les questionnaires de sommeil (ISI et PSQI) seront à remplir à domicile entre les deux sessions évaluatives afin de renseigner les paramètres du sommeil subjectifs. L'ISI se focalise précisément sur les symptômes liés à l'insomnie tandis que le PSQI permet de mesurer la qualité de sommeil subjective globale des participantes. Le questionnaire de Horne et Ostberg [36] permettra de renseigner sur la typologie circadienne.

L'ISI [37] comporte 7 items avec une échelle de Likert évaluant les symptômes associés à l'insomnie. Un score supérieur à 8 indique la présence d'une plainte associée à l'insomnie.

Le PSQI [38] est un questionnaire évaluant 7 composantes associées au sommeil (qualité, durée, latence d'endormissement, efficacité de sommeil, utilisation d'hypnotiques, altérations du sommeil et impact sur le fonctionnement diurne). Le score est compris entre 0 et 21, un score supérieur ou égal à 5 est considéré comme représentant une plainte d'un mauvais sommeil.

Le questionnaire de typologie circadienne de Horne et Ostberg [36] comprend 19 items permettant de déterminer la typologie circadienne (tout à fait du matin, modérément du matin, neutre, modérément du soir, tout à fait du soir) sur la base d'un score total compris entre 16 et 86

L'ensemble de ces 3 questionnaires peut être complété en 15 minutes.

9.1.2 Examen du cycle veille/sommeil

Pour quantifier les périodes d'activité et de repos et donc l'alternance des rythmes veille/sommeil, un actimètre (accéléromètre de la taille d'une montre porté au poignet) avec un agenda du sommeil sera fourni aux participantes 2 semaines avant chaque temps d'évaluation (T1, T2 et T3).

Les principaux paramètres issus des analyses des données d'actimètrie sont l'amplitude et l'acrophase du rythme activité-repos.

9.1.3 Examen du sommeil nocturne

À chaque temps de l'étude, un enregistrement du sommeil par PSG sera réalisé au domicile des participantes ou à l'hôtel au moyen d'un appareil portatif (Siesta, Compumedics). La mise en place du matériel sera réalisée au PFRS par notre équipe de recherche et nécessite environ 2h. Des enregistrements de l'activité cérébrale (électroencéphalographie), des mouvements oculaires, du

rythme cardiaque, du rythme respiratoire, de la saturation en oxygène seront réalisés simultanément tout au long de la nuit. Le scorage du sommeil (c-à-d la caractérisation de l'alternance des différents stades de sommeil) sera réalisé par un technicien spécialiste du sommeil. Son intervention permettra en outre de détecter d'éventuels syndromes d'apnées du sommeil ; les participantes seront redirigées vers un spécialiste le cas échéant. Il sera demandé aux participantes de ne pas conduire en portant le matériel de polysomnographie.

Les principaux paramètres issus des analyses de PSG sont le nombre d'éveils intra-sommeil et l'efficacité du sommeil (temps total de sommeil/temps passé au lit).

9.1.4 Mesures physiologiques liées aux rythmes circadiens et à la réponse au stress

Des recueils salivaires répétés seront réalisés à chaque temps de l'étude (*Tableau 3*) afin d'étudier l'évolution du taux du cortisol salivaire, un des marqueurs des rythmes circadiens, ainsi que certaines réponses liées au stress (*Cortisol Awakening Response*).

Tableau 3 : Mesures du cortisol selon les temps d'évaluation

	**T1 et T2**	**T3**
**Avant et après IRM**	●	
**CAR**<br>- **Au réveil**<br>- **30 min**<br>- **45 min**	●	●
**Coucher**	●	●
**Avant et après repos**	●	●
**Total mesures**	8 mesures	6 mesures

Note. *CAR : Cortisol Awekening Response*

Afin de réaliser cet examen de salive, des systèmes Salivette® seront mis à disposition des participantes. Ce dispositif consiste à introduire un coton dans la bouche et à l'imbiber de salive pendant 2-3 minutes puis de le remettre dans le tube de conservation.

Les participantes recevront une fiche descriptive de la procédure (*Annexe 4*) pour rappel des consignes (ne pas boire, ne pas manger, ne pas fumer, ne pas se brosser les dents dans les 30 minutes précédant le recueil) et un tableau permettant de renseigner l'heure de recueil. Des rappels par SMS seront envoyés aux participantes afin de limiter la charge cognitive. Les recueils réalisés le matin seront réalisés au domicile des participantes ou à l'hôtel et seront placés dans une sacoche isotherme mise à disposition afin d'être conservés à 4°C avant qu'ils ne soient acheminés au laboratoire dans la matinée. Les autres recueils seront réalisés au sein de notre unité de recherche. L'ensemble des échantillons salivaires seront centralisés et stockés à -80°C au Centre François Baclesse de manière sécurisée et avec une traçabilité optimale. Ces échantillons seront transmis à l'équipe d'Hélène Castel à Rouen (Inserm, U1239, Dir : Dr Youssef Anouar) pour doser le taux de cortisol salivaire. A noter que des

paramètres pouvant influencer le taux de cortisol salivaire seront recueillis (âge, indice de masse corporelle, tabagisme, méthodes contraceptives, statut socio-éducatif).

9.2 Connectivité fonctionnelle et structures corticales

Les examens d'IRMa et IRMf seront proposés le même jour aux temps T1 et T2 de l'étude. La procédure complète sera précisée aux participantes avant leur installation. L'acquisition des volumes anatomiques sera réalisée en premier, puis seront réalisées les séquences en IRMf (tâche d'activation puis de repos). Un questionnaire de débriefing hors caméra (10 min) sera ensuite administré. Un enregistrement vidéo sera réalisé afin de filmer le visage des participantes, il permettra de vérifier qu'elles n'auront pas dormi au cours de la session d'IRMf de repos. De même, des mesures physiologiques seront réalisées au cours de l'examen IRM afin de permettre un pré-traitement adéquat des données, les paramètres physiologiques étant connus pour impacter le signal BOLD. Pour cela, un système d'enregistrements de signaux physiologiques (BioPac) sera utilisé afin de mesurer conjointement avec le même boitier : la respiration et l'onde de pouls ou pléthysmographie (saturation du sang en oxygène). La respiration sera mesurée grâce à une sangle placée au-dessus de la poitrine. L'onde de pouls sera mesurée à l'aide d'un oxymètre placé au niveau de l'index.

Les examens d'IRMa et d'IRMf seront réalisés sur l'imageur 3T GE Premier du centre Cyceron. Le GIP CYCERON est détenteur d'une autorisation de lieu de recherches impliquant la personne humaine pour des études en physiologie et physiopathologie chez l'Homme avec les techniques d'imagerie de médecine nucléaire et en résonance magnétique. Cette autorisation à la réalisation d'études en IRM a été délivré par l'ARS en date du 22 septembre 2020 pour une durée de 3 ans.

L'ensemble des mesures retenues est présenté dans le Tableau *4*.

Tableau 4 : Mesures retenues à l'IRM

Séquences IRM	Test	Utilité/Mesures retenues	Durée ≃
**Anatomiques (IRMa)**	T1 3D	Prétraitements des données fonctionnelles<br>Surface corticale	6 min
	T2 Flair	Prétraitements des données de DTI<br>Lésions de substance blanche	6 min
	DTI	Microstructure de la substance blanche	8 min
**Fonctionnelles (IRMf)**	Tâche de repos	Rumination<br>Contrôle pour l'activation<br>Connectivité fonctionnelle	12 min

	Tâche d'activation (ANT)	Performances attentionnelles<br>Contrôle cognitif<br>Connectivité fonctionnelle	20 min
			**Total : 52 min**

Note. DTI : Imagerie de Tenseur de Diffusion ; ANT : Attentional Networks Test

9.2.1 IRMa

Deux types d'images anatomiques de pondération différente seront acquis au cours de cette session d'IRM : T1 et T2 FLAIR. Une séquence avec une pondération en diffusion (DTI) sera également réalisée pendant cette session anatomique.

Ces acquisitions permettront l'étude de l'anatomie cérébrale de l'ensemble des participantes à l'aide de différents indices : densité de substance grise, volume de substance grise, épaisseur corticale, largeur, longueur et profondeur des sillons. Les différentes pondérations auront un rôle dans l'analyse et le traitement des images : recalage des acquisitions fonctionnelles (IRMf) sur l'image anatomique, segmentation des trois groupes tissulaires (substance grise, substance blanche, liquide céphalorachidien). Elles permettront l'obtention d'images de contrastes différents, qui seront également utiles pour mieux évaluer la présence d'anomalies significatives. Ainsi, ces examens permettront d'une part de s'assurer de l'absence de lésions, et d'autre part seront utiles au traitement et l'analyse des données. Un médecin présent lors de la session sera chargé de procéder à cette vérification au moment de l'examen, et d'autoriser le participant à poursuivre ou non l'étude selon les résultats de l'IRMa. En cas de présence d'anomalies, celles-ci seront communiquées au participant et à son médecin traitant qui prendra en charge le suivi médical de la patiente exclue de l'étude.

9.2.2 IRMf

Si aucune anomalie n'a été détectée à l'examen IRMa, les consignes seront données à la participante avant de commencer l'acquisition en ***IRMf au repos***. L'acquisition se déroulera dans un environnement calme et sombre. Les participantes seront invitées à garder les yeux ouverts, à éviter toute tâche cognitive spécifique ou tout autre effort, sans parler ou bouger la tête et à rester éveillées tout le temps de l'examen. Durant cet examen, les participantes peuvent laisser leurs pensées aller librement. À l'issue de cette session, un débriefing hors caméra sera proposé aux participantes, permettant de rapporter précisément la nature des pensées et des sensations corporelles au cours de l'examen d'IRMf au repos. Des activités mentales liées au *self* sont en effet souvent rapportées dans les études au repos, et impliquent notamment des réminiscences autobiographiques, des images mentales, un discours intérieur et une planification de l'avenir. Les participantes devront également décrire si elles étaient pleinement éveillées pendant toute la durée de l'examen. Des questions spécifiquement associées aux phénomènes de rumination seront également proposées. Les participantes devront décrire précisément ces activités mentales à l'aide d'un questionnaire et d'échelles analogiques. L'analyse des données IRMf au repos consistera à modéliser les fluctuations spontanées de basse fréquence du signal BOLD, caractéristiques de l'activité du réseau à l'état de base.

L'acquisition en ***IRMf en activation*** sera organisée en 3 sessions de 5 minutes chacune permettant ainsi de réduire la durée de maintien de l'attention. La tâche d'ANT (*Attentional Network Test*), décrite dans la partie suivante de ce document, sera employée et la connectivité fonctionnelle entre les régions impliquées dans chaque réseau attentionnel, décrit ci-après (*Tableau 5*), sera quantifiée et comparée entre les groupes.

9.3 Tests neuropsychologiques

9.3.1 Efficience cognitive globale

La MoCA sera employée afin de mesurer l'efficience cognitive globale pour chacune des participantes. Elle sera proposée à T1 uniquement.

9.3.2 Tâche de mémoire spatiale

Une tâche de mémoire spatiale informatisée, inspirée de celle de [39] et de [32] sera proposée aux participantes.

Cette tâche consiste en l'apprentissage et le rappel de la position de 4 objets (canard en plastique, chapeau, ballon, chapeau) dans un environnement virtuel circulaire (*Figure 4*). L'environnement représente une prairie délimitée par une frontière (muret en pierre) derrière laquelle différents repères sont visibles (montagne, nuages, soleil). Un repère intra-environnement (cône de signalisation) est également présent. Un temps de familiarisation sera proposé aux participantes avant la réalisation de la tâche.

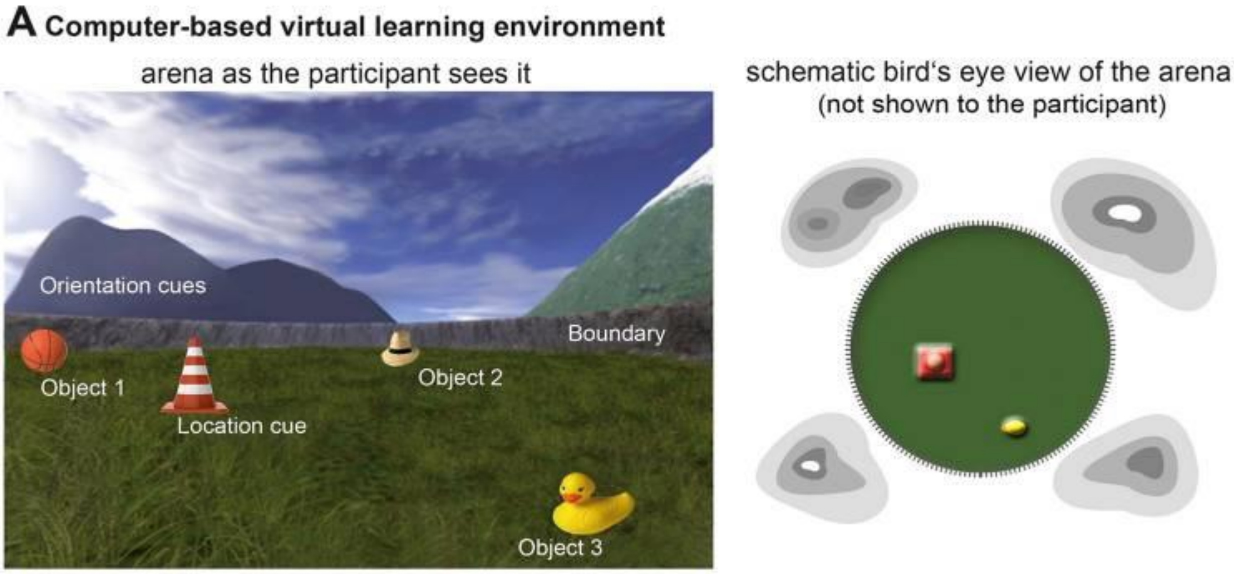

Figure 4 : Tâche de mémoire spatiale (Hilliard et al, 2019)

Cette tâche de mémoire spatiale sera répartie sur les deux jours d'évaluation de façon à proposer un rappel différé après une nuit de sommeil. Ainsi, la tâche proposée se compose des quatre phases décrites ci-après :

Jour 1

Phase 1 – Encodage

Au cours de cette première phase, les participantes seront amenées à explorer l'emplacement des 4 objets (un essai par objet). Une fois que les participantes considèreront avoir retenu l'emplacement de l'objet, il leur sera demandé de collecter l'objet en appuyant sur celui-ci, le faisant ainsi disparaître avant de passer à l'objet suivant.

Phase 2 – Apprentissage

Cette deuxième phase est composée de 3 séries de 4 essais chacune (un essai par objet, ordre pseudo-aléatoire). Pour chaque essai, un des 4 objets apparaît à l'écran seul sur un fond neutre durant 4 sec avant de disparaître. Les participantes devront donc rappeler la position de l'objet mémorisé en appuyant sur l'écran puis un feedback présentant l'emplacement exact de l'objet sera fourni.

Phase 3 – Transfert

Pour cette dernière phase, proposée directement à la suite de la précédente, l'objectif est le rappel de la position des 4 objets dans l'environnement virtuel qui sera manipulé par la modification de la frontière (muret de pierre) ou du repère intra-environnement (cône de signalisation).

- **Situation 1 :** La distance entre le centre de l'environnement et la frontière (muret de pierres) est augmentée de 20%. Le repère intra-environnement (cône de signalisation) n'est pas modifié.
- **Situation 2 :** Le repère intra-environnement (cône de signalisation) est déplacé de 30 virtual meters (vm). La frontière (muret de pierres) n'est pas modifiée.

Chaque objet sera présenté dans les deux situations, soit 8 essais au total au cours de cette phase.

Jour 2 (après nuit de sommeil)

Phase 4 – Rappel différé

Les participantes devront rappeler la position des 4 objets mémorisés la veille au cours d'une phase de rappel similaire à la phase 2 d'apprentissage.

Phase 5 – Transfert

Pour cette dernière phase, proposée directement à la suite de la précédente, l'objectif est le rappel de la position des 4 objets dans l'environnement virtuel qui sera manipulé par la modification de la frontière (muret de pierre) ou du repère intra-environnement (cône de signalisation).

- **Situation 1 :** La distance entre le centre de l'environnement et la frontière (muret de pierres) est augmentée de 20%. Le repère intra-environnement (cône de signalisation) n'est pas modifié.
- **Situation 2 :** Le repère intra-environnement (cône de signalisation) est déplacé de 30 virtual meters (vm). La frontière (muret de pierres) n'est pas modifiée.

Chaque objet sera présenté dans les deux situations, soit 8 essais au total au cours de cette phase.

Mesures recueillies

- Distance (en vm) entre la position rappelée et la position correcte

9.3.3 Attentional Network Test (ANT)

Cette tâche attentionnelle, développée par Fan et collaborateurs [40,41], sera employée comme tâche d'activation en IRMf à T1 et T2 et en mesure comportementale uniquement à T3. L'ANT fournit des mesures de l'efficacité des 3 réseaux distincts de l'attention [42] sous-tendant 1) l'alerte (atteindre et maintenir un état d'alerte), 2) l'orientation (sélectionner les informations sur la base d'entrées sensorielles) et 3) le contrôle exécutif (résolution de conflit entre plusieurs réponses). Ces trois systèmes reposent sur des corrélats neuro-anatomiques et des neurotransmetteurs spécifiques (*Tableau 5*).

Tableau 5 : Mesures de l'ANT

**Réseau attentionnel**	**Mesures recueillies**	**Corrélats neuroanatomiques**
Alerte	TR condition avec indice – TR condition sans indice	Cortex frontal et pariétal droit, formation réticulée
Orientation	TR condition avec indice d'orientation – TR sans indice d'orientation	Lobe pariétal supérieur, thalamus, gyrus frontal moyen
Contrôle exécutif	TR condition congruente – TR condition non-congruente	Cortex cingulaire antérieur, Lobe frontal, notamment le cortex dorso-latéral préfrontal

Note. *ANT : Attentional Network Test ; TR : Temps de Réponse*

Dans cette tâche informatisée (*Figure 5*), il sera demandé aux participantes de déterminer la direction d'une flèche (gauche ou droite) se trouvant parmi d'autres flèches (« *flankers* ») qui se trouvent dans la même direction (situation congruente) ou bien dans la direction opposée (situation non-congruente). Les flèches apparaissent après une durée variable (entre 300 et 11800 ms) au-dessus ou en-dessous d'un point de fixation et sont précédées ou non d'un indice selon 3 conditions possibles : 1) pas d'indice, 2) indice central 3) indice spatial. La tâche est composée de 6 séries de 36 essais + 2 essais non pris en compte.

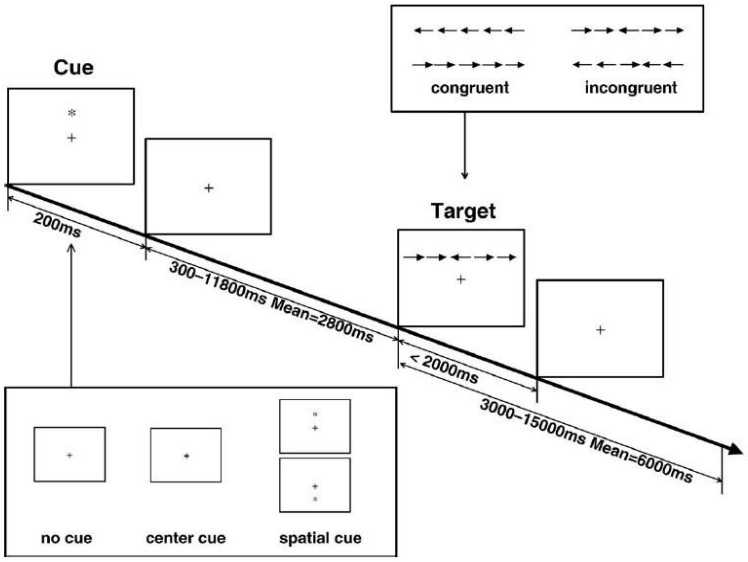

Figure 5 : Schéma de *l'Attentional Network Test*, issu de Fan et al. (2005)

Mesures recueillies

Le temps de réponse (TR) est comptabilisé pour chaque essai, donnant lieu à 3 scores distincts présentés dans le Tableau 5.

9.3.4 Tests neuropsychologiques classiques

Les différents tests neuropsychologiques proposés aux participantes à T1, T2 et T3 sont présentés dans le tableau suivant (*Tableau 6*).

Tableau 6 : Tests neuropsychologiques proposés aux participantes et mesures associées

Fonction cognitive	Test	Mesures retenues	Durée ≃
**Mémoire épisodique**	HVLT [43]<br>• Forme 1 (T1)<br>• Forme 2 (T2)<br>• Forme 4 (T3)	Rappel libre total (/36)	15 min
		Rappel libre différé (/12)	
**Mémoire de travail**	Subtest Mémoire Spatiale (MEM-III) [44]	Note total ordre direct (/16)	5 min
		Note total ordre inverse (/16)	
**Attention**	Double tâche de Baddeley [45]	Tâche d'empan : nombre de séquences correctes ; empan de chiffre	10 min
		Tâche motrice : nombre de cases cochées en 2 min	
		Double tâche : nombre de séquences correctes ; nombre de cases cochées	
	d2-R [46]	Nombre de caractères cibles traités (CCT)	5 min
		Capacité de concentration : CCT - erreurs	
		% d'erreurs : (erreurs / CCT) x 100	
**Fonctions exécutives**	N-Back [47]	Nombre items corrects	5 min
	TMT A et B [45]	Temps réalisation partie A (sec)	5 min
		Temps réalisation partie B - A (sec)	
	Stroop [45]	Temps réalisation Dénomination (sec)	5 min
		Temps réalisation Lecture (sec)	
		Temps réalisation Interférence – Dénomination (sec)	

	Fluences orthographiques [48]<br>• P (T1)<br>• R (T2)<br>• V (T3)	Nombre productions correctes	2 min
**Note.** *HVLT : Hopkins Verbal Learning Test ; MEM-III : Échelle Clinique de mémoire de Wechsler – 3ème edition ; TMT : Trail Making Test*			**Total :**<br>**1h**

L'ensemble des tests neuropsychologiques sera proposé sous la forme de deux sessions (30 min chacune) entre lesquelles une session de questionnaires d'une durée de 20 min ainsi que le retrait de la PSG (45 min) auront lieu, ceci à chaque temps de l'étude et dans la même chronologie (*Figure 6*).

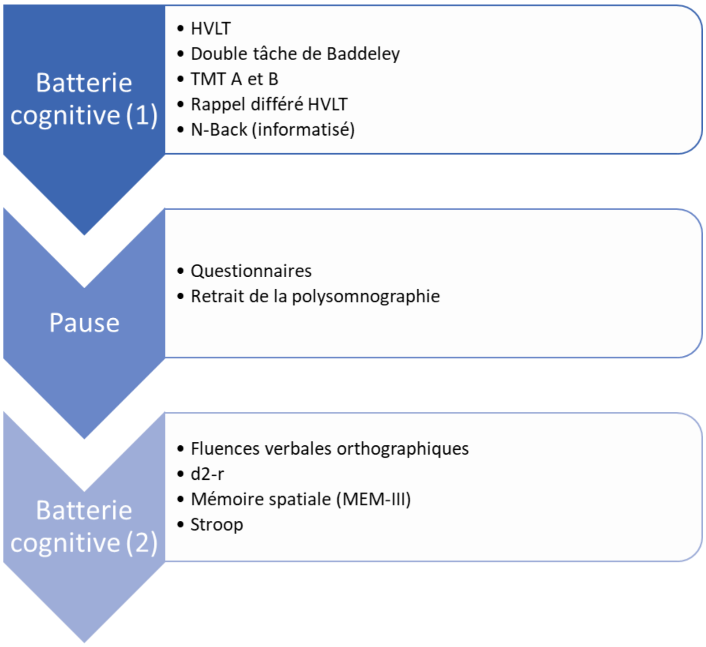

Figure 6 : Chronologie de la batterie cognitive

Note. *MEM-III : Échelle Clinique de mémoire de Wechsler – 3ème edition ; HVLT : Hopkins Verbal Learning Test ; TMT : Trail Making Test*

9.4 Qualité de vie

Les questionnaires visant à mesurer les différents domaines de qualité de vie sont présentés dans le tableau suivant (*Tableau 7*). Certains seront donnés à remplir au domicile tandis que d'autres seront réalisés au cours de deux sessions de questionnaires d'une durée de 20 min environ (*Figure 7*).

Tableau 7 : Questionnaires proposés aux participantes et mesures associées

Domaine	Questionnaires	Mesures (étendue)	Durée de remplissage ≃

**Général**	FACT-G* [49] *patientes uniquement*		Score total (0-108)<br>Bien-être physique (0-28)<br>Bien-être social/familial (0-28)<br>Bien-être émotionnel (0-24)<br>Bien-être fonctionnel (0-28)	5 min
**Douleur**	BPI [50]		Sévérité de la douleur (0-10) ; Interférence de la douleur (0-10)	10 min
**Fatigue**	FACIT-F* [51] *patientes uniquement*		Score (0-52)	5 min
	MFI-20* [52]		Fatigue générale/physique (9 – 45)<br>Fatigue mentale (6 – 30)<br>Réduction des activités (3 – 15)<br>Réduction de la motivation (2 – 10)	5 min
**Somnolence**	KSS		Score total (0-9)	1 min (x4)
**Plainte cognitive**	FACT-Cog* [53]		PCI (0-72) ; QoL (0-16) ; Oth (0-16) ; PCA (0-28)	10 min
**Activité physique**	IPAQ [54]		Score continu<br>Score catégoriel	5 min
**Anxiété – Dépression**	STAI-Y [55]	État	Score état (20-80)	5 min
		Trait	Score trait (20-80)	5 min
	BDI-II [56]		Score total (0-39)	5 min
**Stress perçu**	PSS [57]		Détresse émotionnelle (0-24)<br>Efficacité personnelle (0-16)	5 min
**Stress post-traumatique**	PCL-5 [58] *patientes uniquement*		Score total (0-80)	5 min
**Croissance post-traumatique**	PTGI [59] *patientes uniquement*		Score global (0-105)<br>Relation aux proches (0-35)<br>Nouvelles possibilités (0-25)<br>Forces personnelles (0-20)<br>Appréciation de la vie (0-15)<br>Changement spirituels (0-10)	5 min

**Coping**	Brief COPE [60–62]	Dispositionnel modifiée (avant suspicion de cancer) *patientes uniquement, uniquement à T1*	Score recherche support social (8-32)<br>Score Résolution de problème (4-16)<br>Score Évitement (10-40)<br>Score Pensée positive (6-24)	5 min
		Dispositionnel		5 min
				**Total :**<br>**PATIENTES : 25 min (domicile) + 2×20 min**<br>**FEMMES INDEMNES DE CANCER : 15 min (domicile) + 2 × 20 min**

Note. * : *Questionnaires à remplir au domicile ; Fact-G : The Functional Assessment of Cancer Therapy – General ; BPI : Brief Pain Inventory ; FACIT-F : Functional Assessment of Chronic Illness Therapy – Fatigue ; MFI-20 : MFI-20 : Multidimensional Fatigue Inventory ; KSS : Karolinska Sleepiness Scale ; Fact-Cog : Functional Assessment of Cancer Therapy - Cognitive Function ; IPAQ : International Physical Activity Questionnaire ; PCI : Perceived Cognitive Impairment ; QoL : Impact on Quality of Life ; Oth : Comments from Others ; PCA : Perceived Cognitive Abilities ; STAI-Y : Inventaire d'anxiété état-trait ; BDI-II : Beck Depression Inventory ; PSS : Perceived Stress Scale ; PCL-5 : Posttraumatic Stress Disorder Checklist for DSM-5 ; PTGI : Post-Traumatic Growth Inventory ; Brief COPE : Brief Coping Orientation to Problems Experienced*

9.5 Evaluation de la fonction vestibulaire

Une étude récente a établi que la prévalence des troubles vestibulaires comme les vertiges et les étourdissements était de 15 à 20% chez l'adulte [63]. De plus, des études suggèrent que certains traitements visant le cancer du sein ont des effets sur le fonctionnement vestibulaire [64 – 67].

Le vHIT (vestibular Head Impulse Test [68]) est un test rapide et non invasif permettant d'évaluer de manière objectif la fonction vestibulaire et plus particulièrement celle de chacun des six canaux semi-circulaires individuellement. Il est généralement utilisé afin de détecter d'éventuels dysfonctionnements vestibulaires dans le cadre du bilan des vertiges et troubles de l'équilibre.

Au cours du test, un casque est posé sur la tête de la participante avec des micro-caméras enregistrant le mouvement des yeux. L'opérateur appliquera manuellement des mouvements rapides de la tête dans différentes directions. Pendant le mouvement, le casque enregistrera à la fois les mouvements de la tête et les mouvements des yeux. À partir de ces données, le médecin sera en mesure d'évaluer si la fonction vestibulaire est intacte ou affectée, et quelle en est l'affection le cas échéant.

10 Stimulation Vestibulaire Galvanique (SVG)

10.1 Description de la technique

Le principe de la SVG repose sur l'utilisation de deux électrodes incluses dans le casque de stimulation,

Session 1 - Jour 1

- BDI-II
- BPI
- STAI – État
- STAI – Trait
- PCL-5 (patientes uniquement)

Session 2 - Jour 2

- PSS
- Brief-COPE dispositionnel modifié (avant suspicion de cancer) *(patientes uniquement, T1 uniquement)*
- Brief COPE dispositionnel
- PTGI
- IPAQ

Figure 7 : Questionnaires proposés au cours des 2 sessions d'évaluation

Note : le KSS est proposé au début et à la fin de chaque jour d'évaluation, soit à 4 reprises au total

une cathode (Négative) et une anode (Positive) placées au niveau des os mastoïdes droit et gauche, derrière les oreilles. La première a un rôle excitateur tandis que la seconde a un rôle inhibiteur.

Un courant continu est diffusé à travers ces électrodes permettant de stimuler les fibres irrégulières du nerf vestibulo-cochléaire [29]. Des éponges qui ont préalablement trempé dans une solution saline seront positionnées au niveau de l'emplacement des électrodes, ce qui favorise un meilleur contact avec la peau. L'intensité de courant sera de 1mA [29].

Un casque de stimulation (**Erreur ! Source du renvoi introuvable.**) déjà utilisé dans d'autres protocoles réalisés dans l'unité COMETE sera mis à disposition des participantes au temps T2 de l'étude. Ce casque, commercialisé par l'entreprise Soterix, possède un marquage CE et permet de délivrer une stimulation vestibulaire définie par l'expérimentateur de façon pré-régulée et pré-programmée. Les participantes seront informées du principe d'utilisation et pourront se familiariser avec son utilisation avant de partir à leur domicile avec le matériel. En outre, les participantes auront déjà été familiarisées avec la plateforme *de visio-conférence de l'Université de Caen* au cours du temps T1.

Lors des 2 semaines de stimulation, les participantes recevront un email leur rappelant leur rendez-vous via la plateforme de visio-conférence. Elles pourront alors se connecter à l'heure donnée (le matin, aux alentours de 9h30) et seront en contact visuel avec un des expérimentateurs de l'équipe qui vérifiera le bon positionnement du casque et pourra échanger avec la participante. Le système de stimulation est préprogrammé et ne peut pas être modifié à moins d'avoir un code spécifique connu uniquement de l'équipe de recherche. Une fois la position du casque et des éponges vérifiées, une stimulation sera réalisée pendant 20 minutes durant lesquelles les participantes seront assises et resteront en contact avec l'équipe. À l'issue des 20 minutes, trois échelles visuelles analogiques concernant la douleur, la fatigue et le bien-être seront proposées.

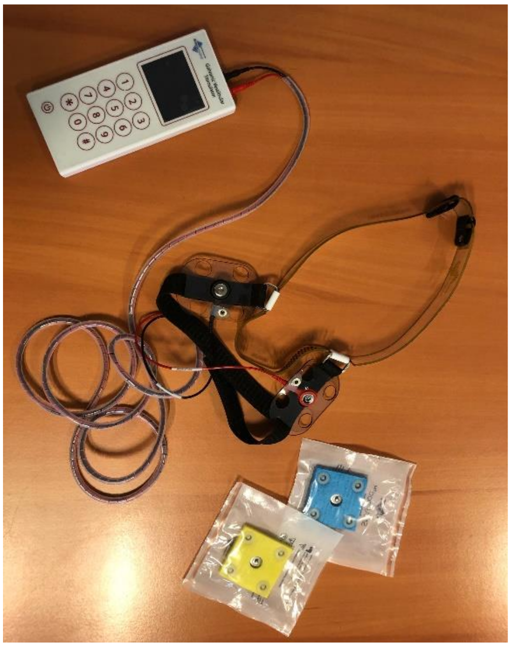

Figure 8 : Ensemble du matériel nécessaire à la stimulation vestibulaire, incluant l'appareil permettant de délivrer le protocole de stimulation da façon contrôlée et préprogrammée, le casque et les éponges délivrées individuellement

10.2 Contre-indications et effets indésirables attendus

La SVG induit des réponses oculomotrices (i.e. nystagmus), posturales et perceptives (e.g. tête qui tourne). Toutefois les réponses observées dépendent du type de stimulation, des électrodes, des individus mais aussi du contexte. Certains individus ressentent principalement les perceptions d'origine canaliculaire (i.e. rotation) tandis que d'autres auront davantage de perceptions issues des afférences otolithiques (i.e. inclinaison). Afin de limiter ces effets secondaires, le seuil de courant électrique sera adapté à chaque participante et nous demanderons aux participantes de réaliser la stimulation dans une pièce éclairée afin de leur permettre d'avoir un point de fixation visuel, ce qui supprimera la réponse visuelle [68]. Enfin, l'échelle de Graybiel [69] ainsi que des échelles visuelles analogiques liées à la fatigue, au bien-être et aux douleurs seront utilisées afin de quantifier le ressenti des patientes et les effets indésirables perçus.

11 VIGILANCE SANITAIRE RÈGLEMENTAIRE

Conformément à la nouvelle réglementation de recherches impliquant la personne humaine, il n'y aura pas de recueil des évènements indésirables graves organisé par le promoteur dans le cadre de la recherche.

Cependant, comme pour toutes les recherches impliquant la personne humaine, le promoteur transmettra aux investigateurs concernés toute information susceptible d'affecter la sécurité des personnes (Art. R1123-52 CSP) et informera sans délai l'autorité compétente et le comité de protection des personnes des faits nouveaux* et, le cas échéant, des mesures prises (Art. R1123-59 du CSP).

Néanmoins il est rappelé à l'attention des professionnels de santé que **la vigilance sanitaire s'applique**, de ce fait tout incident ou effet indésirable suspecté d'être dû à un médicament ou à un autre produit de santé défini à l'article L5311-1 doit être signalé par le professionnel de santé aux réseaux de vigilance qui, après analyse, les déclarent à l'ANSM.

* Fait nouveau : toute nouvelle donnée pouvant conduire à une réévaluation du rapport des bénéfices et des risques de la recherche ou du produit objet de la recherche, à des modifications dans l'utilisation de ce produit, dans la conduite de la recherche, ou des documents relatifs à la recherche, ou à suspendre ou interrompre ou modifier le protocole de la recherche ou des recherches similaires.

12 CONSIDÉRATIONS STATISTIQUES

12.1 NOMBRE DE SUJETS NÉCÉSSAIRES

Le nombre de participantes dans cette étude descriptive et exploratoire ne fait pas l'objet d'un calcul de puissance. Nous prévoyons d'inclure 50 patientes suivies pour un cancer du sein dont 25 traitées par chimiothérapie et 25 non traitées par chimiothérapie, ainsi que 25 femmes volontaires saines (groupe témoin), soit un total de 75 participantes.

12.2 ANALYSE STATISTIQUE

Axe 1 : Afin de caractériser le sommeil des patientes suivies pour un cancer du sein avant et après chimiothérapie adjuvante, les paramètres de sommeil mesurés par polysomnographie (en particulier l'efficacité du sommeil et le nombre d'éveils) ou par questionnaire seront décrits en termes d'effectif pour les variables qualitatives et de moyenne et d'écart type (ou médiane et étendue) pour les variables quantitatives, à T1 et à T2 dans chacun des groupes d'intérêt. De la même façon, les paramètres du rythme circadien mesurés par actimétrie (e.g. amplitude moyenne sur une semaine) ou par dosage de cortisol salivaire seront décrits à chaque temps et par groupe. Afin d'évaluer l'effet du cancer sur le sommeil et le rythme circadien, les paramètres à T1 et T2 seront comparés entre les patients et les contrôles par un test du Chi² ou un test de Student (ou de Wilcoxon). Afin d'évaluer l'effet de la chimiothérapie sur le sommeil et le rythme circadien, les paramètres des patientes traitées par chimiothérapie seront comparés entre T1 et T2 par un test de McNemar ou un test de Student (ou de Wilcoxon) pour séries appariées. Alternativement, l'effet de la chimiothérapie sur les paramètres à T2 pourra être testé dans des modèles linéaires ou logistiques ajustés sur les paramètres à T1. Les relations entre les paramètres de sommeil et les paramètres du rythme circadien seront évalués à l'aide d'une matrice de corrélations (de Pearson ou de Spearman). La relation entre les paramètres de sommeil et les scores aux tests cognitifs et aux évaluations de qualité de vie pourra être évaluée à tout temps à l'aide d'un modèle linéaire mixte (prenant en compte la corrélation entre les mesures répétées d'un même sujet).

Axe 2 : Afin d'évaluer l'effet de la stimulation vestibulaire galvanique sur les rythmes circadiens, l'évolution des paramètres du rythme circadien mesurés par actimétrie au cours des 2 semaines d'intervention (en particulier l'amplitude et la phase du rythme activité-repos) sera décrite par un

modèle linéaire mixte. Le modèle inclura un effet temps, un effet intervention (SVG ou SHAM) et une interaction temps x intervention (paramètre d'intérêt qui permettra d'évaluer si la SVG a un effet sur l'évolution du rythme circadien par rapport au SHAM) et sera ajusté sur le groupe (patients ou contrôles) et éventuellement le traitement (chimiothérapie ou non). Pour répondre aux objectifs secondaires, les paramètres de sommeil, les dosages de cortisol, les capacités cognitives et les évaluations de qualité de vie seront décrits avant (T2) et après intervention (T3) dans chacun des groupes de patientes (SVG ou SHAM) et contrôles (SVG uniquement) et comparés pré-post SVG par un test de McNemar ou un test de Student (ou de Wilcoxon) pour séries appariées. Alternativement, l'effet de la SVG sur le sommeil, les capacités cognitives et la qualité de vie à T3 pourra être testé dans des modèles linéaires ou logistiques ajustés sur les paramètres à T2.

12.3 NON RESPECT DU PROTOCOLE

Toute déviation à ce protocole ainsi que toute modification éventuelle qui pourrait intervenir après le début de l'étude sera discutée dans le rapport d'étude.

13 ASSURANCE QUALITÉ

Afin de garantir l'authenticité et la crédibilité des données conformément aux BPC, le promoteur mettra en place un système d'assurance qualité qui comprend :

- la gestion de l'essai selon les procédures de l'Unité de Recherche clinique,
- le contrôle qualité des données du site investigateur par le moniteur dont le rôle est de vérifier la concordance et la cohérence des données du cahier d'observation par rapport aux documents-source,
- la mise à disposition si le financement le prévoit de personnel dédié dans le service pour aider l'investigateur dans la logistique de l'étude et le recueil des données dans les cahiers d'observation.

14 CONSIDERATIONS ÉTHIQUES ET RÈGLEMENTAIRES

La recherche sera conduite dans le respect de la réglementation française en vigueur, notamment les dispositions relatives à la recherche impliquant la personne humaine biomédicale du Code de la Santé publique, articles L1121-1 et suivants (loi n° 2012-300 du 05/03/2012 telle que modifiée par l'ordonnance n° 2016-800 du 16 juin 2016), les lois de bioéthiques, la loi informatique et libertés, la déclaration d'Helsinki, et les Bonnes Pratiques Cliniques.

14.1 AUTORISATIONS RÈGLEMENTAIRES

Une demande d'autorisation sera adressée par le Promoteur avant le début de l'étude au Comité de Protection des Personnes (CPP). Une information sera donnée à l'Autorité Compétente (ANSM), avec transmission du résumé de l'étude et de l'avis favorable du CPP.

Cette étude entre dans le cadre de la « Méthodologie de Référence » (MR-001) en application des dispositions de l'article 54 alinéa 5 de la loi du 6 janvier 1978 modifiée relative à l'informatique, aux fichiers et aux libertés. Ce changement a été homologué par décision du 5 janvier 2006.

Le Centre François Baclesse respecte la réglementation en vigueur notamment les droits des personnes faisant l'objet d'un traitement au sens du règlement UE n°2016/679 relatif à la protection des données (« RGPD »).

Toute modification substantielle du protocole, concernant les objectifs de l'étude, son plan, la population, les examens ou des aspects administratifs significatifs, nécessitera l'approbation de l'investigateur coordonnateur, du promoteur, l'avis favorable du CPP et l'information de l'autorité compétente avant toute mise en œuvre.

14.2 INFORMATION DE LA PARTICIPANTE ET FORMULAIRE DE CONSENTEMENT ÉCLAIRÉ ÉCRIT

Les participantes seront informées de façon complète et loyale, en des termes compréhensibles, des objectifs et des contraintes de l'étude, des risques éventuels encourus, des mesures de surveillance et de sécurité nécessaires, de leurs droits de refuser de participer à l'étude ou de la possibilité de se rétracter à tout moment.

Toutes ces informations figurent sur un formulaire d'information et de consentement remis au sujet (patiente ou femme indemne de cancer). Le consentement libre, éclairé et écrit du sujet sera recueilli par l'investigateur, ou un médecin qui le représente avant l'inclusion définitive dans l'étude. Une copie du formulaire d'information et de consentement signé par les deux parties sera remise au sujet, l'autre copie sera conservée par l'investigateur. Pour toute modification substantielle du protocole, concernant les objectifs de l'étude, son plan, la population, les examens ou des aspects administratifs significatifs, un nouveau consentement des personnes participant à la recherche sera recueilli si nécessaire.

14.3 CONDUITE DE L'ÉTUDE ET RESPONSABILITÉS DES INVESTIGATEURS

L'investigateur principal de chaque établissement concerné s'engage à conduire l'essai clinique conformément au protocole qui a été approuvé par le CPP. L'investigateur ne doit apporter aucune modification au protocole sans l'autorisation du promoteur et sans que les CPP ait donné un avis favorable sur les modifications proposées.

Il est de la responsabilité de l'investigateur principal :

- ✓ de fournir au promoteur son curriculum vitae ainsi que ceux des co-investigateurs,
- ✓ d'identifier les membres de son équipe qui participent à l'essai et de définir leurs responsabilités,
- ✓ de démarrer le recrutement des patients après autorisation du promoteur,
- ✓ de faire le maximum pour inclure le nombre requis de patients dans les limites de la période de recrutement établie.

Il est de la responsabilité de chaque investigateur :

- ✓ de recueillir le consentement éclairé daté et signé personnellement par le patient avant toute procédure de sélection spécifique à l'essai,
- ✓ de compléter régulièrement les cahiers d'observation (CRF) pour chacun des patients inclus dans l'essai et de laisser au moniteur mandaté par le promoteur un accès direct aux documents-source afin que ce dernier puisse valider les données du CRF,

- ✓ d'accepter les visites régulières du moniteur et éventuellement celles des auditeurs mandatés par le promoteur ou des inspecteurs des autorités de tutelle.

Toute la documentation relative à l'étude (protocole, consentements, cahiers d'observation, dossier investigateur, etc.…), ainsi que les documents originaux (résultats de laboratoire, radiologies, comptes-rendus de consultations, rapports d'examens cliniques pratiqués, etc.) doivent être mis à disposition du promoteur ou des personnes agissant pour le compte du promoteur et être détenus dans un lieu sûr et considérés comme du matériel confidentiel.

L'archivage des données sera sous la responsabilité de l'investigateur et selon la législation en vigueur. Les documents qui devront être archivés sont le protocole et annexes incluant les amendements éventuels, les formulaires d'information et consentements originaux signés, les questionnaires, les CRF ainsi qu'une liste d'identification des patients. L'ensemble de ces documents sera conservé pendant une durée minimale de 15 ans après la fin de l'étude.

14.4 PROPRIÉTÉS DES DONNÉES ET CONFIDENTIALITÉ

L'investigateur s'engage, pour lui-même et pour toutes les personnes amenées à suivre le déroulement de l'essai, à garantir la confidentialité de toutes les informations portant sur le projet jusqu'à la publication des résultats de l'essai. Cette obligation de confidentialité ne s'appliquera pas aux renseignements que l'investigateur sera amené à communiquer aux participantes dans le cadre de leur participation à l'essai ni aux informations déjà publiées.

L'investigateur s'engage à ne pas publier, divulguer ou utiliser, de quelque façon que ce soit, directement ou indirectement, les informations scientifiques ou techniques en relation avec l'essai.

L'essai ne pourra faire l'objet d'aucun commentaire écrit ou oral sans l'accord du promoteur ; l'ensemble des informations communiquées ou obtenues pendant la réalisation de l'essai appartenant de plein droit au promoteur qui pourra librement en disposer.

15 TRAITEMENT ET CONSERVATION DES DONNÉES

15.1 RECUEIL ET TRAITEMENT DES DONNÉES

La gestion des données sera réalisée par le Centre de Traitement des Données (CTD) du Cancéropôle Nord-Ouest. Le CTD met à disposition un progiciel de gestion de bases de données dédié à la recherche clinique : *Ennov Clinical* (version 7.5.10, ENNOV / CLINSIGHT, 33155 Cenon, France).

Ce progiciel, qui s'appuie sur une architecture de base de données Oracleä, est conçu pour la gestion globale des études cliniques et épidémiologiques, répond aux exigences réglementaires liées à ce type d'étude. L'instance *Ennov Clinical* du CTD est validée dans son environnement informatique. Un plan de validation des données sera élaboré conjointement par l'Unité de Recherche Clinique et le Centre de Traitement des Données et décrira de manière détaillée les contrôles à exécuter pour chaque variable.

Une base de données spécifique à l'étude sera créée, testée et validée avant le début de la saisie. Toutes les informations requises par le protocole doivent être consignées sur les cahiers d'observation papier -ou sur le cahier d'observation électronique- sous la responsabilité de l'investigateur principal et une explication doit être apportée pour chaque donnée manquante. Les données devront être

renseignées dans ces cahiers au fur et à mesure qu'elles sont obtenues, et le promoteur prendra en charge le monitoring.

Les données seront ensuite contrôlées par le CTD conformément au plan de validation des données.

La base de données sera gelée après contrôle de qualité final, puis exportée au format adapté pour l'analyse statistique selon une procédure automatisée et validée.

Des analyses statistiques descriptives sont prévues et seront réalisées avant la fin de la réalisation de l'étude. Celles-ci n'interfèreront en aucune manière avec le déroulé du protocole.

15.2 ARCHIVAGE

Le promoteur doit assurer l'archivage des documents essentiels sur la conduite de l'étude dans des conditions assurant leur sécurité, pendant la durée minimale prévue par les BPC, soit 15 ans après la fin de la recherche.

Ces documents sont le protocole et annexes incluant les amendements éventuels, les formulaires d'information et consentements originaux signés, les questionnaires, les CRF, les documents de suivi, les analyses statistiques, le rapport final de l'étude.

15.3 PROPRIÉTES DES DONNÉES ET RÈGLES DE PUBLICATION

Les résultats de cette étude, propriété du promoteur (Centre François Baclesse), seront publiés sous forme d'articles scientifiques. Les publications concernant ou issues de cette recherche seront communiquées et soumises pour relecture par les coordinateurs de l'étude à l'ensemble des investigateurs.

Les auteurs comprendront les investigateurs ayant inclus le plus de patientes, le biostatisticien qui aura réalisé l'analyse des données (le cas échéant), le chef de projet, ainsi que les participants qui ont apporté une contribution substantielle au développement de l'étude, à l'analyse et à l'interprétation des résultats et/ou à la rédaction du manuscrit. Aucune publication ne sera faite sans l'accord du coordinateur et du promoteur. L'organisme ayant contribué au financement de l'étude sera mentionné dans la publication.

Les remerciements aux membres du comité de surveillance seront indiqués dans la publication finale.

Le rang de publication sera défini en fonction de l'investissement fourni dans l'élaboration et la réalisation de l'étude.

Des publications spécifiques aux études ancillaires pourront être réalisées.

Ces travaux seront la propriété de tous les auteurs et seront mis à leur disposition pour la réalisation de communications et de publications transversales.

Les publications relatives aux résultats d'éventuelles études annexes seront soumises à l'accord préalable de l'investigateur coordonnateur et du méthodologiste ; elles seront postérieures à la publication de l'étude principale, qui devra être citée en référence.

16 FINANCEMENT ET ASSURANCE

16.1 BUDGET DE L'ÉTUDE

Les éventuels frais supplémentaires visés au Code de la Santé Publique font l'objet d'une convention négociée entre le CFB et le représentant de l'établissement en tenant compte des moyens financiers dont dispose le CFB dans le cadre de son activité de promotion publique.

Cependant, le CFB assure l'organisation de l'étude et la prise en charge de la fourniture du matériel suivant (protocole, cahier d'observation, dossier investigateur) nécessaire à la conduite de l'étude.

Dans le cas où du matériel ou des traitements sont fournis par d'autres partenaires, les conditions doivent être précisées dans la convention de l'étude.

16.2 ASSURANCE

Le Promoteur, a souscrit pour toute la durée de l'étude une assurance garantissant sa propre responsabilité civile ainsi que celle de tout médecin impliqué dans la réalisation de l'étude. Il assurera également l'indemnisation intégrale des conséquences dommageables à la recherche pour la personne qui s'y prête et ses ayants droit, sauf preuve à sa charge que le dommage n'est pas imputable à sa faute ou à celle de tout intervenant, sans que puisse être opposé le fait d'un tiers ou le retrait volontaire de la personne qui avait initialement consenti à se prêter à la recherche (cf. article L 1121-10).

17 RÉFÉRENCES BIBLIOGRAPHIQUES

1. Roth, T. Insomnia: Definition, prevalence, etiology, and consequences. *Journal of Clinical Sleep Medicine* **3**, S7–S10 (2007).
2. Fiorentino, L. & Ancoli-Israel, S. Insomnia and its treatment in women with breast cancer. *Sleep Medicine Reviews* **10**, 419–429 (2006).
3. Fleming, L. *et al.* Insomnia in breast cancer: a prospective observational study. *Sleep* **42**, (2019).
4. Perrier, J., Duivon, M., Rauchs, G. & Giffard, B. Le sommeil dans les cancers non cérébraux : revue de la littérature, mécanismes potentiels et perspectives pour mieux comprendre les troubles cognitifs associés. *Médecine du Sommeil* **18**, 90–103 (2021).
5. Hsiao, F.-H. *et al.* A longitudinal study of diurnal cortisol patterns and associated factors in breast cancer patients from the transition stage of the end of active cancer treatment to post-treatment survivorship. *Breast (Edinburgh, Scotland)* **36**, 96–101 (2017).
6. Carpenter, J. S., Gilchrist, J. M., Chen, K., Gautam, S. & Freedman, R. R. Hot flashes, core body temperature, and metabolic parameters in breast cancer survivors. *Menopause (New York, N.Y.)* **11**, 375–381 (2004).
7. Martin, T. *et al.* Rest activity rhythms characteristics of breast cancer women following endocrine therapy. *Sleep* zsab248 (2021) doi:10.1093/sleep/zsab248.
8. Ancoli-Israel, S. *et al.* Sleep, fatigue, depression, and circadian activity rhythms in women with breast cancer before and after treatment: a 1-year longitudinal study. *Supportive Care in Cancer: Official Journal of the Multinational Association of Supportive Care in Cancer* **22**, 2535–2545 (2014).

9. Payne, J., Piper, B., Rabinowitz, I. & Zimmerman, B. Biomarkers, fatigue, sleep, and depressive symptoms in women with breast cancer: a pilot study. *Oncology Nursing Forum* **33**, 775–783 (2006).

10. Liu, L. *et al.* Decreased Health-Related Quality of Life in Women With Breast Cancer Is Associated With Poor Sleep. *Behavioral Sleep Medicine* **11**, 189–206 (2013).

11. Liu, L. *et al.* Fatigue and sleep quality are associated with changes in inflammatory markers in breast cancer patients undergoing chemotherapy. *Brain Behavior and Immunity* **26**, 706–713 (2012).

12. Liu, L. *et al.* The longitudinal relationship between fatigue and sleep in breast cancer patients undergoing chemotherapy. *Sleep* **35**, 237–245 (2012).

13. Li, W. *et al.* Disruption of sleep, sleep-wake activity rhythm, and nocturnal melatonin production in breast cancer patients undergoing adjuvant chemotherapy: prospective cohort study. *Sleep Medicine* **55**, 14–21 (2019).

14. Beck, S. L. *et al.* Sleep quality after initial chemotherapy for breast cancer. *Supportive Care in Cancer: Official Journal of the Multinational Association of Supportive Care in Cancer* **18**, 679–689 (2010).

15. Kuo, H.-H., Chiu, M.-J., Liao, W.-C. & Hwang, S.-L. Quality of sleep and related factors during chemotherapy in patients with stage I/II breast cancer. *Journal of the Formosan Medical Association = Taiwan Yi Zhi* **105**, 64–69 (2006).

16. Madsen, M. T., Huang, C. & Gögenur, I. Actigraphy for measurements of sleep in relation to oncological treatment of patients with cancer: a systematic review. *Sleep Medicine Reviews* **20**, 73–83 (2015).

17. Parker, K. P. *et al.* Sleep/Wake patterns of individuals with advanced cancer measured by ambulatory polysomnography. *Journal of Clinical Oncology: Official Journal of the American Society of Clinical Oncology* **26**, 2464–2472 (2008).

18. Williams, R. L., Karacan, I. & Hursch, C. J. *Electroencephalography (EEG) of human sleep: clinical applications*. (John Wiley & Sons, 1974).

19. Roscoe, J. A. *et al.* Few changes observed in polysomnographic-assessed sleep before and after completion of chemotherapy. *Journal of Psychosomatic Research* **71**, 423–428 (2011).

20. Diekelmann, S. & Born, J. The memory function of sleep. *Nature Reviews. Neuroscience* **11**, 114–126 (2010).

21. Wilckens, K. A., Woo, S. G., Kirk, A. R., Erickson, K. I. & Wheeler, M. E. The role of sleep continuity and total sleep time in executive function across the adult lifespan. *Psychology and aging* **29**, 658–665 (2014).

22. van Dalfsen, J. H. & Markus, C. R. The influence of sleep on human hypothalamic-pituitary-adrenal (HPA) axis reactivity: A systematic review. *Sleep Medicine Reviews* **39**, 187–194 (2018).

23. Kreutz, C., Schmidt, M. E. & Steindorf, K. Effects of physical and mind-body exercise on sleep problems during and after breast cancer treatment: a systematic review and meta-analysis. *Breast Cancer Research and Treatment* **176**, 1–15 (2019).

24. Garland, S. N. *et al.* Sleeping well with cancer: a systematic review of cognitive behavioral therapy for insomnia in cancer patients. *Neuropsychiatric Disease and Treatment* **10**, 1113–1124 (2014).

25. Besnard, S. *et al.* The balance of sleep: Role of the vestibular sensory system. *Sleep Medicine Reviews* **42**, 220–228 (2018).

26. Martin, T. *et al.* Vestibular loss disrupts daily rhythm in rats. *Journal of Applied Physiology (Bethesda, Md.: 1985)* **118**, 310–318 (2015).

27. Martin, T. *et al.* Exploration of Circadian Rhythms in Patients with Bilateral Vestibular Loss. *PLOS ONE* **11**, e0155067 (2016).

28. Pasquier, F. *et al.* Effect of vestibular stimulation using a rotatory chair in human rest/activity rhythm. *Chronobiology International* **37**, 1244–1251 (2020).

29. Utz, K. S., Dimova, V., Oppenländer, K. & Kerkhoff, G. Electrified minds: transcranial direct current stimulation (tDCS) and galvanic vestibular stimulation (GVS) as methods of non-invasive brain stimulation in neuropsychology–a review of current data and future implications. *Neuropsychologia* **48**, 2789–2810 (2010).

30. Wilkinson, D., Nicholls, S., Pattenden, C., Kilduff, P. & Milberg, W. Galvanic vestibular stimulation speeds visual memory recall. *Experimental Brain Research* **189**, 243–248 (2008).

31. Wilkinson, D., Ferguson, H. J. & Worley, A. Galvanic vestibular stimulation modulates the electrophysiological response during face processing. *Visual Neuroscience* **29**, 255–262 (2012).

32. Hilliard, D. *et al.* Noisy galvanic vestibular stimulation modulates spatial memory in young healthy adults. *Scientific Reports* **9**, 1–11 (2019).

33. Pasquier, F., Denise, P., Gauthier, A., Bessot, N. & Quarck, G. Impact of Galvanic Vestibular Stimulation on Anxiety Level in Young Adults. *Frontiers in Systems Neuroscience* **13**, 14 (2019).

34. Voros, J. L. *et al.* Galvanic Vestibular Stimulation Produces Cross-Modal Improvements in Visual Thresholds. *Front Neurosci* **15**, 640984 (2021).

35. Putman, E. J., Galvan-Garza, R. C. & Clark, T. K. The Effect of Noisy Galvanic Vestibular Stimulation on Learning of Functional Mobility and Manual Control Nulling Sensorimotor Tasks. *Front Hum Neurosci* **15**, 756674 (2021).

36. Horne, J. A. & Ostberg, O. A self-assessment questionnaire to determine morningness-eveningness in human circadian rhythms. *International Journal of Chronobiology* **4**, 97–110 (1976).

37. Morin, C. M. *Insomnia: Psychological assessment and management.* (Guilford press, 1993).

38. Buysse, D. J. *et al.* The Pittsburgh Sleep Quality Index: a new instrument for psychiatric practice and research. *Psychiatry res* **28**, 193–213 (1989).

39. Doeller, C. F., King, J. A. & Burgess, N. Parallel striatal and hippocampal systems for landmarks and boundaries in spatial memory. *Proceedings of the National Academy of Sciences* **105**, 5915–5920 (2007).

40. Fan, J., McCandliss, B. D., Sommer, T., Raz, A. & Posner, M. I. Testing the Efficiency and Independence of Attentionnal Networks. **14**, 340–347 (2002).

41. Fan, J., McCandliss, B. D., Fossella, J., Flombaum, J. I. & Posner, M. I. The activation of attentional networks. *NeuroImage* **26**, 471–479 (2005).

42. Posner, M. I. & Petersen, S. E. The Attention System of the Human Brain. *Annals of Neuroscience* **13**, 25–42 (1990).

43. Rieu, D., Bachoud-Lévi, A.-C., Laurent, A., Jurion, E. & Dalla Barba, G. Adaptation française du « Hopkins verbal learning test ». *Revue Neurologique* **162**, 721–728 (2006).

44. Wechsler III, D. MEM-III. *Échelle clinique de mémoire de Wechsler: manuel. les Éd. du Centre de psychologie appliquée* (2001).

45. Godefroy, O. & le GREFEX. *Fonctions exécutives et pathologies neurologiques et psychiatriques. Marseille, Solal* (2008).

46. Brickenkamp, R., Schmidt-Atzert, L. & Liepmann, D. *D2-R: test d'attention concentrée*. (Éditions Hogrefe France, 2015).

47. Gevins, A. S. & Cutillo, B. C. Neuroelectric evidence for distributed processing in human working memory. *Electroencephalography and Clinical Neurophysiology* **87**, 128–143 (1993).

48. Cardebat, D., Doyon, B., Puel, M., Goulet, P. & Joanette, Y. Evocation lexicale formelle et sémantique chez des sujets normaux. Performances et dynamiques de production en fonction du sexe, de l'âge et du niveau d'étude. *Acta Neurologica Belgica* **90**, 207–217 (1990).

49. Cella, D. F. *et al.* The Functional Assessment of Cancer Therapy scale: development and validation of the general measure. *Journal of Clinical Oncology: Official Journal of the American Society of Clinical Oncology* **11**, 570–579 (1993).

50. Cleeland, C. The Brief Pain Inventory. *Pain Research Group* 143–147 (1991).

51. Webster, K., Cella, D. & Yost, K. The Functional Assessment of Chronic Illness Therapy (FACIT) Measurement System: properties, applications, and interpretation. *Health and Quality of Life Outcomes* **1**, 79 (2003).

52. Gentile, S., Delarozière, J. C., Favre, F., Sambuc, R. & San Marco, J. L. Validation of the French 'multidimensional fatigue inventory' (MFI 20). *European Journal of Cancer Care* **12**, 58–64 (2003).

53. Joly, F. *et al.* French version of the Functional Assessment of Cancer Therapy-Cognitive Function (FACT-Cog) version 3. *Supportive Care in Cancer* **20**, 3297–3305 (2012).

54. Craig, C. L. *et al.* International physical activity questionnaire: 12-country reliability and validity. *Medicine & science in sports & exercise* **35**, 1381–1395 (2003).

55. Spielberger, C. D. Manual for the State-Trait Anxiety Inventory STAI (form Y)(' self-evaluation questionnaire'). (1983).

56. Beck, A. T., Ward, C. H., Mendelson, M., Mock, J. & Erbaugh, J. An Inventory for Measuring Depression, Archives of General Psychiatry, 4. 561–571 (1961).

57. Cohen, S., Kamarck, T. & Mermelstein, R. Perceived stress scale. *Measuring stress: A guide for health and social scientists* **10**, 1–2 (1994).

58. Weathers, F. W. *et al.* The PTSD Checklist for DSM-5 (PCL-5). *Scale available from the National Center for PTSD at www. ptsd. va. gov* (2013).

59. Tedeschi, R. G. & Calhoun, L. G. The Posttraumatic Growth Inventory: Measuring the positive legacy of trauma. *Journal of traumatic stress* **9**, 455–471 (1996).

60. Carver, C. S. You want to measure coping but your protocol's too long: Consider the brief COPE. *International Journal of Behavioral Medicine* vol. 4 92–100 (1997).

61. Muller, L. & Spitz, E. Évaluation multidimensionnelle du coping: Validation du Brief COPE sur une population française. *Encephale* **29**, 507–518 (2003).

62. Baumstarck, K. *et al.* Assessment of coping: A new french four-factor structure of the brief COPE inventory. *Health and Quality of Life Outcomes* **15**, 1–9 (2017).

63. Neuhauser HK. [Epidemiology of dizziness and vertigo]. Der Nervenarzt. 2009 Aug;80(8):887-894.

64. Medina HN, Liu Q, Cao C, Yang L. Balance and vestibular function and survival in US cancer survivors. Cancer. 2021 Nov 1;127(21):4022-4029.

65. Monfort SM, Pan X, Patrick R, Singaravelu J, Loprinzi CL, Lustberg MB, Chaudhari AMW. Natural history of postural instability in breast cancer patients treated with taxane-based chemotherapy: A pilot study. Gait Posture. 2016 Jul;48:237-242.

66. Wampler MA, Topp KS, Miaskowski C, Byl NN, Rugo HS, Hamel K. Quantitative and clinical description of postural instability in women with breast cancer treated with taxane chemotherapy. Arch Phys Med Rehabil. 2007 Aug;88(8):1002-8.

67. Winters-Stone KM, Torgrimson B, Horak F, Eisner A, Nail L, Leo MC, Chui S, Luoh SW. Identifying factors associated with falls in postmenopausal breast cancer survivors: a multi-disciplinary approach. Arch Phys Med Rehabil. 2011 Apr;92(4):646-52.

68. Curthoys, I. S. & MacDougall, H. G. What galvanic vestibular stimulation actually activates. *Frontiers in neurology* **3**, 117 (2012).

69. Graybiel, A., Wood, C. D. & Miller II, E. F. *Diagnostic criteria for grading the severity of acute motion sickness*. vol. 1030 (Naval Aerospace Medical Institute, Naval Aerospace Medical Center, 1968).

18 ANNEXES

Annexe 1 : Note d'information et formulaire de consentement destinés aux patientes

Annexe 2 : Note d'information et formulaire de consentement destinés aux sujets volontaires sains

Annexe 3 : Trajet entre Cyceron et le Pôle des Formations et de Recherche en Santé (PFRS) à T1 et T2

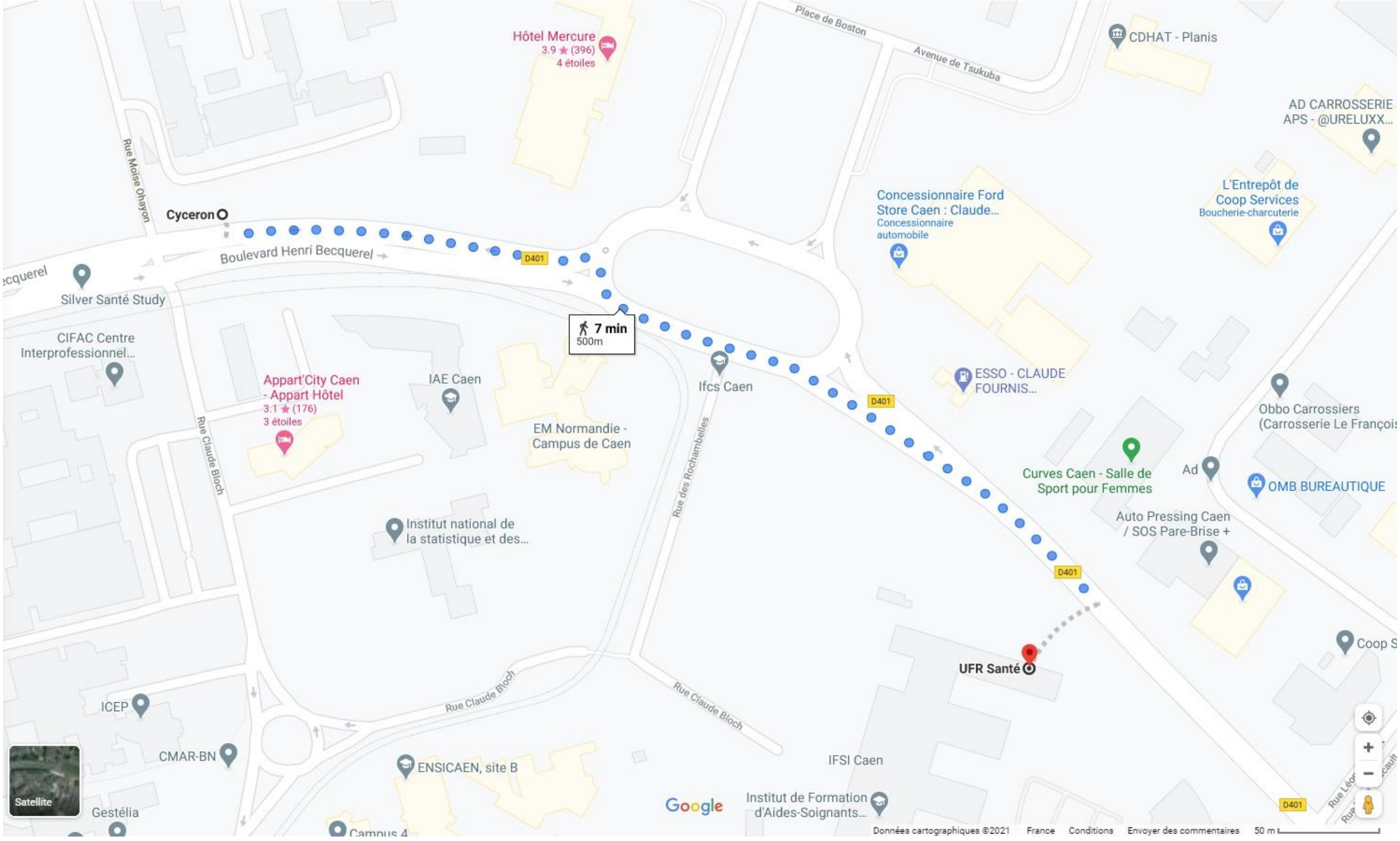

Annexe 4 : Mode d'emploi à destination des participantes pour les mesures salivaires

Mode d’emploi : recueil salivaire

Pourquoi ?

Les recueils réalisés à différents moments de la journée vont nous permettre d’avoir des informations sur une hormone contenue dans la salive, le cortisol qui est en lien avec les rythmes circadiens et le stress.

Quand ?

Vous réaliserez plusieurs recueils avec l’équipe de recherche mais aussi en autonomie à votre domicile aux moments suivants :

1. Au coucher
2. Au réveil
3. 30 minutes après le réveil
4. 45 minutes après le réveil

Nous vous enverrons des rappels par SMS

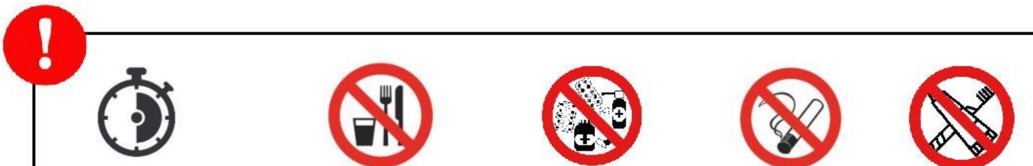

30 minutes avant, **NE PAS** : **boire et manger** **prendre un médicament** **fumer** **se laver les dents**

Comment ?

1. Enlever le bouchon et sortir le coton
2. Mâcher le coton pendant 2-3 minutes, jusqu’à ce que vous ne puissiez plus éviter d’avaler la salive produite
3. Remettre le tampon imbibé de salive dans son support puis dans le récipient de centrifugation et refermer avec le bouchon
4. Remplir les informations nécessaires **(date et heure du prélèvement)** sur l’étiquette, et la coller, ainsi que sur le tableau de suivi
5. Placer les Salivette® au congélateur pour les conserver (le soir) ou dans la sacoche isolante avec les pains de glace pour ramener les échantillons (le matin)

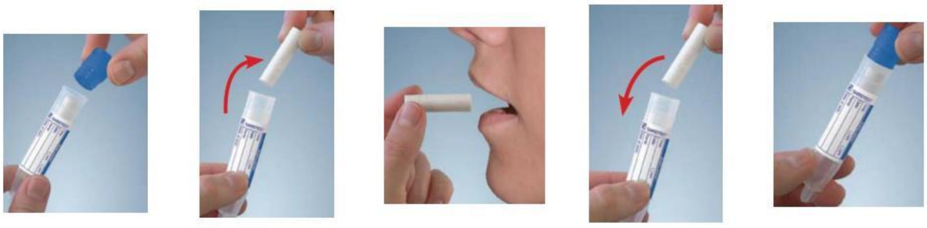

Schéma du système Salivette®

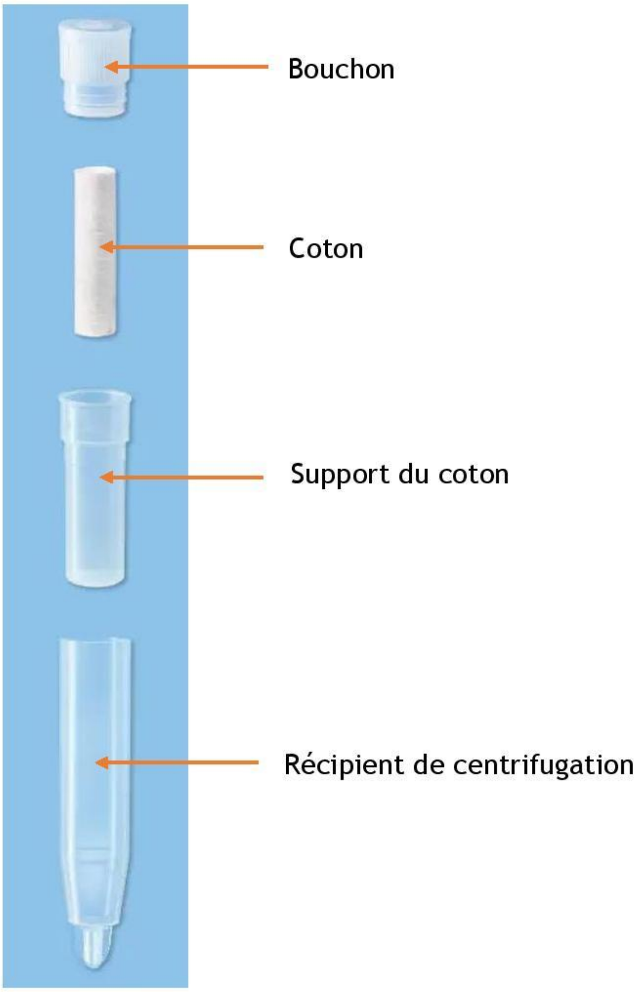

Supplement: S2 File — (PDF) [file pone.0306462.s004.pdf]
